# Synthesis, properties, and chemoselective reactions of an AlH–BH functional group

**DOI:** 10.1039/d5sc05133a

**Published:** 2025-08-15

**Authors:** Wenbang Yang, Andrew J. P. White, Mark R. Crimmin

**Affiliations:** a Department of Chemistry, Molecular Sciences Research Hub 82 Wood Lane, Shepherds Bush London W12 0BZ UK m.crimmin@imperial.ac.uk

## Abstract

A new alumaborane compound containing a {AlH–BH} functional group has been prepared by reaction of the aluminium(I) complex [{(ArCMeN)_2_CH}Al] (Ar = 2,6-*i*-Pr_2_C_6_H_3_) with a boron dihydride species supported by an anionic chelating κ^2^-*N*,*N* ligand. Spectroscopic analysis of the product suggests that it exists as a mixture of isomers in solution with the ligand on boron coordinating in both a κ^1^ and κ ^2^ fashion. The dialumane analogue, containing a {AlH–AlH} functional group, was also prepared. Comparison of the structure and bonding of these species by DFT calculations suggests that reactivity of the AlH–BH moiety should be governed by the nucleophilicity of the Al–H and Al–B bonds along with the Lewis acidity imparted by the partially available 2p of boron. Chemoselective reactions are observed with CO_2_, CNXyl (Xyl = 2,6-Me_2_C_6_H_3_), and PhCN. While CO_2_ inserts selectively into the Al–H bond to generate a formate, the more Lewis basic substrates CNXyl and PhCN react through initial coordination at boron and insertion into the Al–B bond. In the case of PhCN, an unusual pathway to generate 1,3,2-diazaborole compound is reported. Our work delivers the first insight into the reactivity of the {AlH–BH} functional group and provides a rational framework for further developments.

## Abstract 2

A new alumaborane compound containing a {AlH–BH} functional group has been prepared by reaction of the aluminium(I) complex [{(ArCMeN)_2_CH}Al] (Ar = 2,6-*i*-Pr_2_C_6_H_3_) with a boron dihydride species supported by an anionic chelating κ^2^-*N*,*N* ligand.

## Graphical abstract

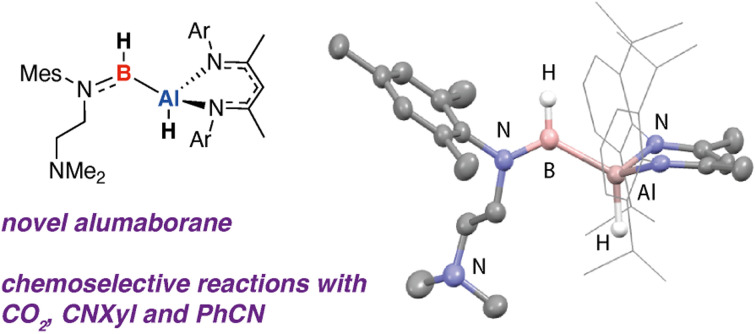

## Introduction

**Fig. 1:**
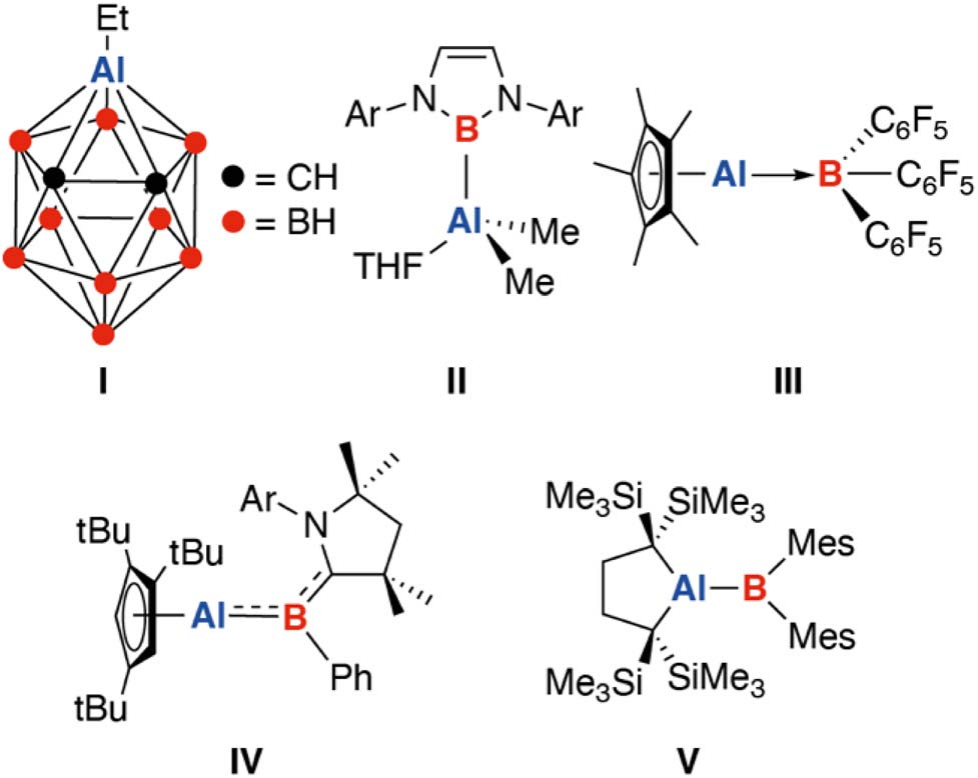
Known classes of molecules containing Al–B σ-bonds.

Compounds containing direct heteronuclear bonds between boron and aluminium are fundamentally interesting but vastly unexplored. To date only a few structurally characterised compounds containing direct Al–B bonds have been reported. These can be broadly classified as boron hydride clusters in which a skeletal position is occupied by an aluminium atom (I),^1–5^ borylalumanes constructed from reaction of a boryl anion and aluminium(III) fragment (II),^6–12^ Lewis-acid/Lewis base adducts between either an aluminylene moiety and trivalent boron site or borylene and trivalent aluminium site (III),^13–17^ compounds formed by combination of two low-oxidation state group 13 fragments (IV),^18,19^ or alumaboranes (V) (Fig. 1).^17,20–24^

Arguably the least well understood of these compound types are alumaboranes. These contain a X_2_B–AlX_2_ functional group (X = monoanionic ligand). Given that both group 13 elements are electropositive and typically employed in Lewis acidic reagents, fundamental questions arise as to the polarisation of the Al–B bond and the chemoselectivity of onwards reactions. To date there are only a handful of structurally characterised examples of compounds containing the X_2_B–AlX_2_ functional group and very little description of reactivity has been reported. The alumaboranes [{(ArCMeN)_2_CH}Al(H)Bpin] (Ar = 2,6-*i*-Pr_2_C_6_H_3_, Bpin = O_2_C_2_Me_4_) and [Cp′Al(Cl)–B(Cl)Ar] (Cp′ = 1,3,5-*t*-Bu_3_C_5_H_2_; Ar = 2,6-(2,4,6-*i*-Pr_3_C_6_H_2_)_2_C_6_H_3_) have been reported by the Nikonov and Braunschweig groups respectively, but reactivity is yet to be described.^17,19^ Hill and coworkers documented an alumaborane from reaction of an aluminyl anion with MeOBpin.^22^ Yamashita and coworkers have isolated a tetraorgano alumaborane of the form Mes_2_B–AlR_2_ (*R*_2_ = –{C(SiMe_3_)_2_}_2_CH_2_CH_2_–) and shown that it reacts to deoxygenate dimethyl sulfoxide or CO through initial coordination of the substrate to either the aluminium and boron centres.^21^ A strained alumaborane supported by a 1,8-disubstituted naphthalene ligand has also been reported and effects the scission of carbon–heteroatom multiple bonds of benzophenone and an isocyanide.^24^

In this paper, we report the isolation and structural characterisation of compound containing a unique heteronuclear {AlH–BH} functional group. We compare the bonding and solution dynamics of this species to a heavier analogue in which the boron site is replaced by a second aluminium atom. The {AlH–BH} motif shows a diverse range with reactions determined by the nucleophilic behaviour of either Al^*δ*+^–H^*δ*−^ or Al^*δ*+^–B^*δ*−^ bonds, and in certain cases initiated through substrate coordination to boron.

## Results and discussion

**Scheme 1:**
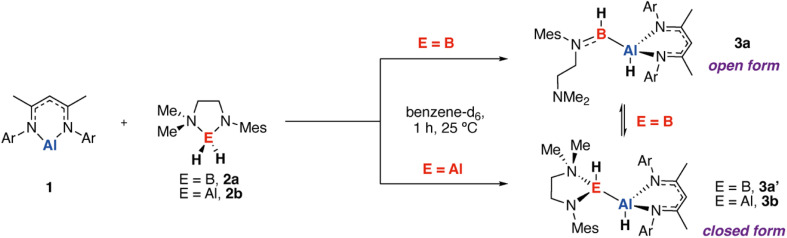
Synthetic route to alumaborane 3a/3a′ along with analogous dialumane 3b.

Reaction of the aluminium(I) complex 1 (ref. 25) with a 1 equiv. of boron dihydride 2a in benzene-d_6_ led to consumption of the starting materials and production of the corresponding alumaborane after 1 h at 25 °C (Scheme 1). We have previously studied the solution dynamics of 2a along with its reactions with a series of electrophiles (carbonyls, imines, aryl fluorides).^26^ These studies strongly suggest that 2a can convert between open and closed forms through reversible coordination of the dimethylamino group. As such its reactivity is characterised by both the hydridic nature of the B–H bond and Lewis acidity due to the partially available p-orbital at boron. Reaction with 1 appears to occur through selective insertion of the aluminium(I) reagent into a B–H bond of 2a, generating an 85 : 15 equilibrium mixture of alumaboranes 3a (open form) and 3a′ (closed form) as measured by ^1^H NMR spectroscopy at 298 K in C_6_D_6_. A closely related reaction is known to occur between 1 and HBpin (pin = pinacolato).^20^3a was assigned as the open-chain form based on the ^11^B{^1^H} NMR chemical shift of *δ* = 56.8 ppm which is consistent with a three-coordinate boron environment. This resonance is shifted upfield significantly from the parent dihydrido borane found at *δ* = −0.5 ppm and is agreement the literature value for [{(ArCMeN)_2_CH}Al(H)Bpin] of *δ* = 34.9 ppm.^20^ Further support for 3a being the more stable species in solution was provided by DFT calculations which suggest that 3a is +6.0 kcal mol^−1^ more stable than the closed form 3a′.

**Fig. 2:**
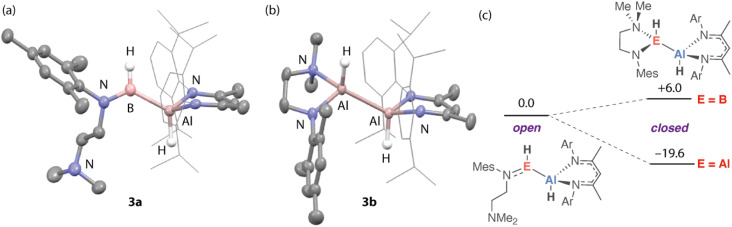
Crystal structures of (a) 3a and (b) 3b, H-atoms with the exception of hydrides omitted for clarity. (c) A comparison of the relative energies of open and closed isomeric structures for these compounds based on DFT calculations. G09: B3PW91-D3/6-311+G**/PCM (benzene)//B3PW91-D3/6-31G**/6-311+G*/SDDAll (Al). Gibbs free energy kcal mol^−1^.

In the solid-state, the product crystallises as the isomer 3a (Fig. 2). The aluminium centre demonstrates tetrahedral symmetry, while boron is three-coordinate and trigonal planar. The Al–B bond length is 2.1349(18) Å and matches well with the range of 2.123(2) to 2.191(2) Å established for the handful of known alumaboranes (V).^17,20–24^ The alumaborane functional group adopts a geometry with the two hydride ligands demonstrating a near perfect anti-periplanar relation across the Al–B bond. The B–N bond of 3a is 1.394(2) Å, shortened from that of 1.512(3) Å in 2a likely due to increased B=N π-bonding to alleviate unsaturation at the three-coordinate boron centre.

As a point of comparison, we sought to prepare the dialumane analogue of 3a. Reaction of 1 with the aluminium(III) dihydride 2b^27^ in benzene-d_6_ gave exclusive formation of 3b after 1 h at 25 °C (Scheme 1). Unlike 3a/3a′ the unsymmetrical dialumane compound 3b showed no evidence of existing in both open and closed forms in solution, rather a single isomer assigned as the closed form was observed. In the solid-state, 3b demonstrates two tetrahedral aluminium sites, with the hydride ligands of the dialumane functional group again aligned in an anti-periplanar fashion (Fig. 2). The Al–Al bond length of 3b of 2.6203(6) Å is within the sum of the covalent radii and in close alignment with structurally related dialumane compounds.^28,29^ DFT calculations again support the assignment, with the closed form now predicted to be −19.6 kcal mol^−1^ more stable than the hypothetical open form. These differences between the alumaborane {AlH–BH} and dialumane {AlH–AlH} likely derive from the larger size of the Al atom and its ability to expand its coordination sphere more easily than B, along with the more favourable Al–N binding interaction.

**Fig. 3:**
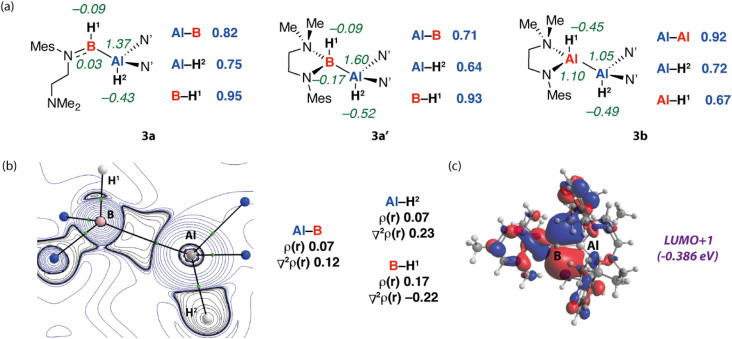
(a) NBO calculations on 3a, 3a′ and 3b showing NPA charges and WBI for key atoms and bonds. (b) QTAIM contour plot showing the Laplacian of *ρ*(*r*) for 3a. (c) Kohn–Sham molecular orbitals of 3a with the HOMO delocalised across B–H, Al–B and Al–H sigma bonding interactions and the LUMO+1 showing a significant contribution from the B 2p lone-pair.

Further calculations were used to better understand the electronic structure of 3a, 3a′, and 3b, with a specific emphasis on how reversible ligand coordination and exchanging the B atom for Al impacts the bonding and charge distribution. NBO calculations suggest that the Al^*δ*+^–B^*δ*−^ bond of 3a is covalent as evidenced by the high Wiberg Bond Index (WBIs) but polarised with electron-density shifted toward the more electronegative boron atom based on the NPA charges (Fig. 3a). Both hydrides of 3a have a negative charge but that connected to boron is less negative than that connected to aluminium and shows higher covalent bonding character. For comparison, 3a′ demonstrates a near identical electronic structure, albeit with slightly lower WBIs and increased charge separation brought on by the increase of coordination number at boron. As might be expected, despite the unsymmetrical ligand environments, the electron distribution in 3b appears more symmetrical than 3a/3a′. The aluminium atoms bear similar NPA charges to one another as do the hydrides of 3b. The Al–Al bond has a WBI very close to one as would be expected for an apolar covalent bond. QTAIM calculations further support these bonding models. Specifically, for compound 3a, comparison of the electron density and Laplacian of electron density at the bond critical points between B and H^1^ an Al and H^2^ suggest that the former bond is more covalent with less charge separation based on the more positive value for *ρ*(*r*) and negative ∇^2^*ρ*(*r*) (Fig. 3b). Comparison of the frontier molecular orbitals for 3a and 3a′ suggests that the former possesses a vacant low-lying orbital at boron (LUMO+1, Fig. 3c). In combination, these data suggest that the alumaborane functional group of 3a is best considered as a covalent polar moiety, with likely reactivity driven by the Lewis acidity at boron and/or polar nature of the Al^*δ*+^–B^*δ*−^ and Al^*δ*+^–H^*δ*−^ bonds.

**Scheme 2:**
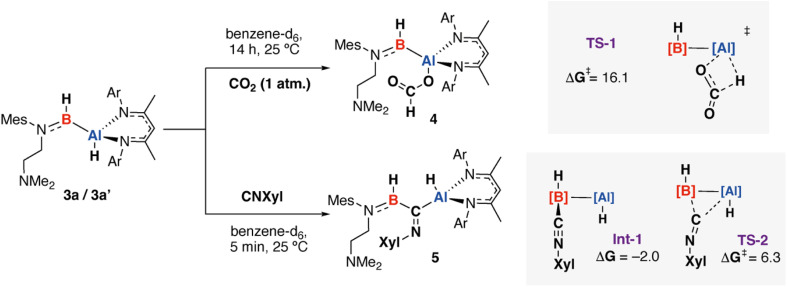
Chemoselective reactions of alumaborane 3a/3a′ with CO_2_ and CNXyl (Xyl = 2,6-dimethylphenyl), along with calculated transition states for insertion into Al–H and Al–B bonds. G09: B3PW91-D3/6-311+G**/PCM (benzene)//B3PW91-D3/6-31G**/6-311+G*/SDDAll (Al). Nitrogen-based ligands on B and Al are truncated for clarity. Gibbs free energy kcal mol^−1^.

**Fig. 4:**
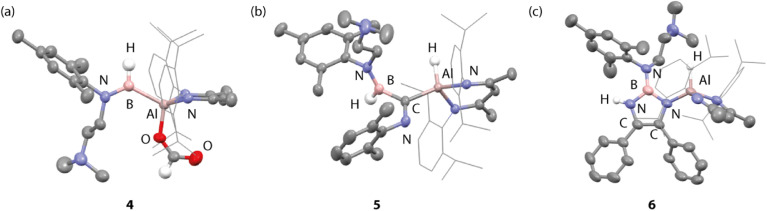
Crystal structures of (a) 4, (b) 5, and (c) 6. H-atoms, with exception of key positions, omitted for clarity.

Curious as to whether these predictions would be borne out by experiment a series of reactions of 3a/3a′ mixtures with unsaturated electrophiles were investigated. Addition of CO_2_ (1 atm.) to 3a/3a′ led to selective insertion into the Al^*δ*+^–H^*δ*−^ bond to form the formate complex 4. In contrast, reaction with 2,6-dimethylphenyl isocyanide (CNXyl) resulted in selective insertion into the Al^*δ*+^–B^*δ*−^ bond to form 5. In both reactions, 3a and 3a′ were both consumed further supporting the notion that these species can equilibrate under the reaction conditions (Scheme 2, Fig. 4a and b). Prior work has demonstrated that alumoxane dihydride complexes react with CO_2_ non-selectively to form formate ligands that bridge two aluminium centres,^30^ while dialumenes react with CO_2_ to form cycloaddition products.^31^ Similarly, dialumanes are reported to react with isocyanides to generate linear and cyclic trimerization products.^32^ Hence, the chemoselectivity observed with 3a/3a′ seems to be complementary to known reagents that contain Al–Al bonds.

The observed products are consistent with the expected charge localisation and most nucleophilic sites in 3a, in particular, the absence of the reactivity of the B–H bond is consistent with reactivity patterns of three-coordinate boron compounds where these sites show limited nucleophilicity. There is clear difference in chemoselecitivity that leads to 4 and 5. Modelling the pathway to form 4 by DFT leads to identification of an open transition state involving nucleophilic attack of the hydride onto the central carbon of CO_2_*via*TS-1 (Δ*G*^‡^ = 16.1 kcal mol^−1^). All attempts to find alternative transition states involving initial coordination of CO_2_ to the boron atom of 3a failed. In contrast, reaction of CNXyl could be modelled through a low energy pathway involving initial coordination of the isonitrile to boron to form Int-1 followed by insertion into the Al^*δ*+^–B^*δ*−^ bond *via*TS-2 (Δ*G*^‡^ = 8.3 kcal mol^−1^). Attempts to identify an open transition state involving direct attack of the aluminium hydride failed. These calculations suggest that the Lewis acidity at the boron site of 3a might be an important factor in determining selectivity. The weak Lewis-base CO_2_ does not appear to coordinate to 3a easily, leading to direct reaction at the aluminium hydride, the strong Lewis base, CNXyl, in contrast, coordinates at boron before reacting at the adjacent Al^*δ*+^–B^*δ*−^ bond. This is perhaps unsurprising given the difference in charge density at the O and N atoms within these substrates. Further NBO analysis of Int-1 suggests that the coordination event increases the polarisation of the Al^*δ*+^–B^*δ*−^ bond likely further increasing its reactivity.

**Scheme 3:**
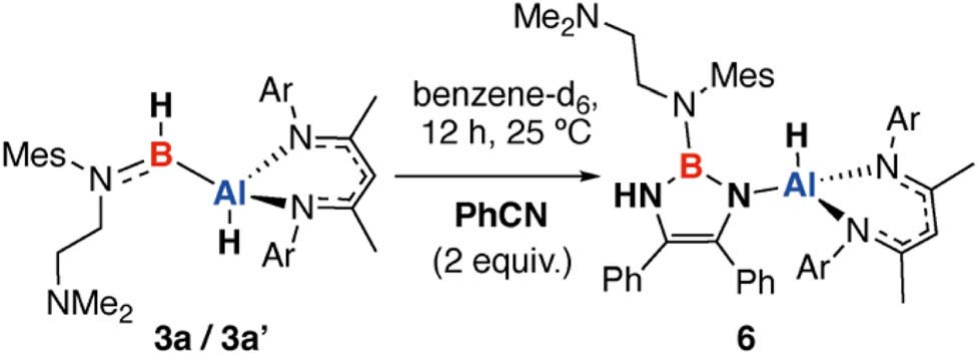
Reaction of alumaborane 3a/3a′ with PhCN to form a product containing a 1,3,2-diazaborole motif.

Mixtures of 3a/3a′ also react selectivity with benzonitrile (PhCN) in a 1 : 2 reaction stoichiometry to form a single product 6 after 12 h at 25 °C in benzene-d_6_ (Scheme 3). 6 is formed from the coupling of two benzonitrile units to form a 1,3,2-diazaborole ring and is characterised by diagnostic resonances in the ^1^H and ^11^B{^1^H} NMR spectra at *δ* = 5.32 ppm (s, 1H) and *δ* = 28.4 ppm assigned to the NH moiety and B nucleus. In the solid-state, bond lengths within the 1,3,2-diazaborole are consistent with the formulation of C–N single and C=C double bonds of 1.394(3) to 1.430(3) and 1.357(4) Å respectively (Fig. 4c). For comparison a known analogue with no substituents on nitrogen and has an alternative structure with C=N lengths varying from 1.296(6) to 1.295(6) Å and a long C–C bond of 1.513(7) Å.^33^ To the best of our knowledge this is the first example of the reductive coupling of benzonitrile to form a diazaborole with this substitution pattern. Prior work has demonstrated that 1,4,2-diazaboroles can be accessed from the three-component coupling of an isonitrile, benzonitrile, and a borane such as (2,4,6-(CF_3_)_3_C_6_H_2_)BH_2_ or Cy_2_BH.^34,35^

**Fig. 5:**
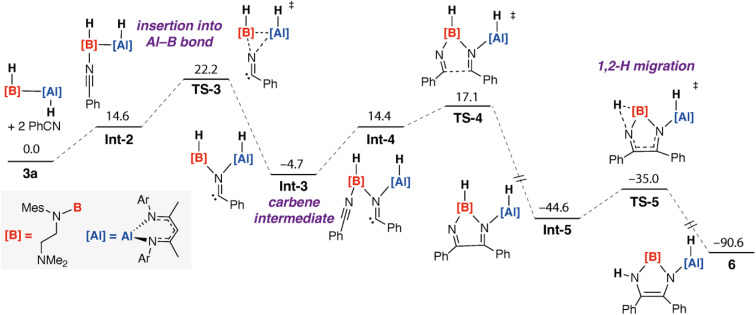
Calculated pathway for the reaction of 3a with 2 equiv. of PhCN to form 6. G09: B3PW91-D3/6-311+G**/PCM (benzene)//B3PW91-D3/6-31G**/6-311+G*/SDDAll (Al). Nitrogen-based ligands on B and Al are truncated for clarity. Gibbs free energy kcal mol^−1^.

Potential mechanisms to form 6 were investigated using DFT calculations. The lowest energy pathway identified involves coordination driven insertion of the substrate into the Al–B bond of 3a (Fig. 5).

Addition of the first equivalent PhCN to 3a occurs through an initial coordination through an associative pathway. Coordination occurs with a weakening of the Al–B as evidenced by the lowering of the WBI from 0.82 in 3a to 0.51 in Int-2. The electron-deficiency at B appears to be alleviated by the adjacent nitrogen centre and the B–N and N=C WBIs take values of 0.87 and 2.39. From Int-2, insertion into the Al–B bond occurs through TS-3 with Δ*G*^‡^_298K_ = 22.2 kcal mol^−1^ and led to the 1,1-difunctionalised insertion product Int-3. Formation of Int-3 is exergonic with respect to the starting reagents with 
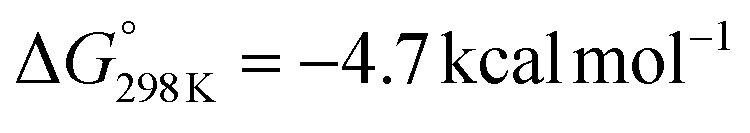
. Int-3 is a carbene intermediate. The N–C–C_aryl_ angle of the benzonitrile unit bends to 118° and NBO analysis shows charge localisation on the carbene site with NPA charge of 0.13. Carbon–carbon bond formation evolves from this intermediate through coordination of a second equivalent of benzonitrile to form Int-4 followed by nucleophilic attack of the carbene on the electrophilic carbon of the coordinated benzonitrile *via*TS-4 with Δ*G*^‡^_298K_ = 21.8 kcal mol^−1^. This barrier is very similar to that calculated for the first insertion step. Both would be expected to be accessible but slow reactions at room temperature and the similar barrier heights are consistent with the lack of observation of the putative carbene intermediate Int-3. Carbon–carbon bond formation through TS-4 establishes an α-diimine motif in the product Int-5, subsequent 1,2-migration of the hydrogen atom from boron to nitrogen through TS-5 generates the experimentally observed product 6 with the 1,3,2-diazaborole established through a shift of electron-density in the ring system that occurs simultaneously with the 1,2-hydrogen atom migration. Carbene intermediates have been invoked previously in carbon–carbon bond forming reactions from alumaboranes with isocyanides^24^ and low-valent aluminium complexes with isonitriles.^36^ Experimental evidence has also been put forward for generation of carbene species from insertion of CO into metal–boron bonds.^37,38^

## Conclusions

In summary, we report a rare example of an alumaborane compound containing a previously unknown {AlH–BH} functional group. This species is compared to an isostructural dialumane analogue through single crystal X-ray diffraction and computational approaches (NBO, AIM, MO analysis). The calculations suggest that the Al–B bond of the {AlH–BH} fragment is covalent with electron-density polarised toward boron. Of the hydride sites, the Al–H is expected to be the most nucleophilic, with the B–H bond showing little hydridic character. Despite the potential for complex reactivity of the {AlH–BH} moiety, we show that this species undergoes highly chemoselective reactions with a handful of substrates. Hence, CO_2_ selectively inserts into the Al–H bond to form the corresponding formate complex, while CNXyl and PhCN react selectively through insertion into the Al–B bond. The divergent behaviour is explained through the propensity of the substrate to coordinate to the Lewis acidic boron site of the {AlH–BH} group, with both CNXyl and PhCN predicted to bind to this fragment before inserting into the Al–B bond, while CO_2_ does not. In the case of PhCN, a 1 : 2 reaction stoichiometry is observed to generate an unusual 1,3,2-diazaborole motif.

## Author contributions

WY conducted all experimental and computational work. AJPW collected and refined single crystal data. All authors were involved in writing the manuscript.

## Conflicts of interest

The authors declare that they have no conflicts of interest.

## Supplementary Material

SC-016-D5SC05133A-s001

SC-016-D5SC05133A-s002

SC-016-D5SC05133A-s003

## Data Availability

CCDC 2363830–2363834 contain the supplementary crystallographic data for this paper.^39–43^ Synthetic procedures, kinetic experiments, NMR spectra of all compounds, crystallographic data, and computational methods (PDF). Cartesian coordinates of the DFT-optimised structures (XYZ). X-ray crystallographic data (CCDC entries 2363830–2363834) (CIF). https://www.ccdc.cam.ac.uk/data_request/cif, or by emailing data_request@ccdc.cam.ac.uk. See DOI: https://doi.org/10.1039/d5sc05133a.
