# Supplementary material for: Synthesis, properties, and chemoselective reactions of an AlH–BH functional group
Source: Chem Sci. 2025 Aug 15;16(37):17193–9. doi: 10.1039/d5sc05133a (PMC12394903; doi:10.1039/d5sc05133a)
Supplement: SC-016-D5SC05133A-s002 [file SC-016-D5SC05133A-s002.pdf]

Supplementary Information (SI) for Chemical Science.
This journal is © The Royal Society of Chemistry 2025

Supporting Information

Synthesis, Properties, and Chemoselective Reactions of an AlH–BH Functional Group

Wenbang Yang, Andrew J. P. White and Mark R. Crimmin*

m.crimmin@imperial.ac.uk

Molecular Sciences Research Hub, Imperial College London, 82 Wood Lane, Shepherds Bush, W12 0BZ, UK.

Table of Contents

1) General Experimental **S3**

2) Experimental Methods **S4**

2.1) Preparation of Compounds S4

3) Single Crystal X-ray Diffraction Data **S14**

4) DFT Studies **S21**

4.1) Computational methods S21

4.2) Calculated Reaction Pathway S22

4.3) Optimised structures S29

4.4) Other Possible Mechanism Pathway S36

4.5) NBO Analysis S38

4.6) QTAIM Analysis S46

5) NMR Spectroscopy **S49**

6) XYZ Coordinates **S55**

7) References **S91**

1) General experimental

Standard Schlenk line and glovebox techniques were used for all manipulations under an inert atmosphere of dinitrogen or argon unless otherwise stated. NMR scale reactions were performed in J. Young NMR tubes. A MBraun Labmaster glovebox was employed, operating at <0.1 ppm O_2 and <0.1 ppm H_2O.

Instruments: ^{1}H, ^{13}C NMR spectra were recorded on BRUKER 400 MHz or 500 MHz machines. All peaks are referenced against residual solvent and values are quoted in ppm. Data were processed using the MestReNova software. Where needed, chemical shifts were assigned with the assistance of 2D NMR (COSY, HSQC, HMBC, DEPTQ) spectra. The coupling constants (J) are reported in hertz (Hz). The following abbreviations are used to define multiplicities: s (singlet), d (doublet), t (triplet), q (quadruplet), hept. (heptet), dd (doublet of doublets), ddd (doublet of doublets of doublets), dt (doublet of triplets), td (triplet of doublets), m (multiplet).

Single crystal X-ray data was obtained on Agilent Diffraction Xcalibur PX Ultra A and Xcalibur 3 E diffractometers, and the structures were refined using the SHELXTL, SHELX-97, and SHELX-2013 program systems.

Infrared spectra were obtained on a Cary630 spectrometer (placed with in an MBraun glovebox) from crystalline solids or benzene thin-films on an ATR cell.

Elemental analyses were performed by Elemental Labs (https://www.elementallab.co.uk/).

Chemicals: Solvents were dried over activated alumina from a solvent purification system (SPS) based upon the Grubbs design and de-gassed before use. Glassware was dried for >6 h prior to use at 120 °C. Benzene-d_6 was de-gassed and stored over 3 Å molecular sieves before use. All reagents and chemicals were acquired from Sigma Aldrich (Merck), Fluorochem, or VWR and used without further purification unless specified. ^{Dipp}BDIAl (**1**)[S1], ^{Mes}NN-BH_2 (**2a**, ^{Mes}NN = $[MesNCH_2CH_2NMe_2]^-$) [S2] and ^{Mes}NN-AlH_2 (**2b**) [S3] were prepared by literature procedures.

2) Experimental Methods

2.1 Preparation of Compounds

Preparation of 3a/3a'

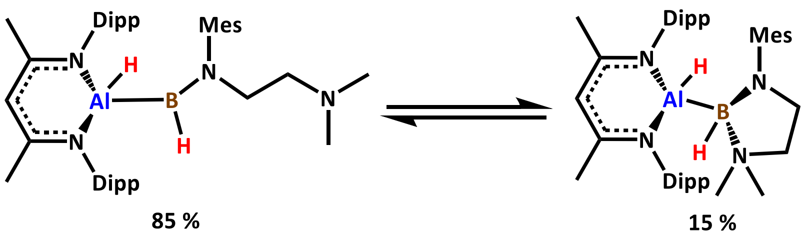

In a glovebox, **1** (40.0 mg, 0.090 mmol, 1 equiv.) and **2a** (19.6 mg, 0.090 mmol, 1 eqiuv.) were dissolved in C_6D_6 (1.0 mL) and transferred to a J. Young NMR tube. The mixture was allowed to react for 1h at 25 °C. A ^{1}H NMR spectrum was taken at this time point and showed the full conversion the starting materils to a 85:15 mixture of **3a**:**3a'**. The J. Young NMR tube was returned to the glovebox, the reaction mixture was decanted into a 20 mL scintillation vial, the solvent was then removed under vacuum and the crude product dissolved in 1 mL Et_2O/n-pentane (1:2 v: v) mixture. The solution was filtered through a glass fibre into a 4 mL vial and the solution stored in the glovebox freezer (–35 °C) for 2 days. Colourless crystals (**3a**) were successfully obtained. The filtrated crystals were washed with cold n-pentane (3 x 1mL) and then dried in vacuo. **Yield: 36 mg,** 0.054 mmol, 60%. Dissolving these crystals in C_6D_6 revealled reformation of both open and closed-forms of the product **3a** and **3a'** in a 85:15 ratio.

NMR data of **3a**:

^{1}H NMR (C_6D_6, 298 K, 400 MHz) δ: 7.16 – 7.14 (m, 2H, ArC***H***) 7.11 (dd, $^3J_{H\text{-}H}$ = 5.8, 3.6 Hz, 4H, ArC***H***), 6.62 (s, 2H, Mes-***H***), 6.01 (s, 1H, Al-***H***), 5.17 (s, 1H, B-***H***), 4.97 (s, 1H, C***H***{C(CH$_3$)}$_2$), 3.81 – 3.74 (m, 2H, MesNC***H$_2$***CH$_2$N(CH$_3$)$_2$), 3.58 (hept, $^3J_{H\text{-}H}$ = 6.8 Hz, 2H, C***H***(CH$_3$)$_2$), 3.56 (hept, $^3J_{H\text{-}H}$ = 6.8 Hz, 2H, C***H***(CH$_3$)$_2$), 2.35 – 2.30 (m, 2H, MesNCH$_2$C***H$_2$***NMe$_2$), 2.02 (s, 6H, 2x NC(C***H$_3$***)), 1.98 (s, 3H, Mes-C***H$_3$***), 1.83 (s, 6H, Mes-C***H$_3$***), 1.61 (s, 6H, N(C***H$_3$***)$_2$), 1.50 (d, $^3J_{H\text{-}H}$ = 6.7 Hz, 6H, (CH(C***H$_3$***)$_2$), 1.26 (d, $^3J_{H\text{-}H}$ = 6.9 Hz, 6H, (CH(C***H$_3$***)$_2$), 1.20 (d, $^3J_{H\text{-}H}$ = 6.8 Hz, 6H, (CH(C***H$_3$***)$_2$), 1.10 (d, $^3J_{H\text{-}H}$ = 6.9 Hz, 6H, (CH(C***H$_3$***)$_2$).

^{13}C NMR (C_6D_6, 298 K, 101 MHz) δ: 169.7 (2x CH{***C***(CH$_3$)}$_2$), 145.6 (Ar-***C***), 143.5 (Ar-***C***), 141.4 (Ar-***C***), 132.5 (Ar-***C***), 129.2 (Ar-***C***H), 126.8 (Ar-***C***H), 124.6 (Ar-***C***H), 124.1 (Ar-***C***H), 97.1 (2x ***C***H{C(CH$_3$)}$_2$), 59.7 (MesN***C***H$_2$CH$_2$N(CH$_3$)$_2$), 57.7 (MesNCH$_2$CH$_2$N(***C***H$_3$)$_2$), 45.9 (MesNCH$_2$***C***H$_2$N(CH$_3$)$_2$), 29.1 (2x ***C***H(CH$_3$)$_2$), 28.2 (2x ***C***H(CH$_3$)$_2$), 26.8 (2x CH(***C***H$_3$)$_2$), 24.9 (2x CH(***C***H$_3$)$_2$), 24.4 (2x CH(***C***H$_3$)$_2$), 24.1 (2x CH(***C***H$_3$)$_2$), 23.0 (2x NC(***C***H$_3$)), 20.7 (Mes-***C***H$_3$), 18.6 (Mes-***C***H$_3$). Some Ar***C*** resonances are overlapping and cannot be observed.

^{11}B NMR (C_6D_6, 298 K, 128 MHz) δ: 56.8 (bs, ***B***H)

Selected NMR data of **3a'**:

^{1}H NMR (C_6D_6, 298 K, 400 MHz) δ: 7.16 – 7.14 (m, 2H, ArC***H***), 7.11 – 7.09 (m, 4H, ArC***H***), 6.66 (s, 2H, Mes-***H***), 6.01 (s, 1H, Al-***H***), 4.85 (s, 1H, B-***H***), 4.82 (s, 1H, C***H***{C(CH$_3$)}$_2$), 4.01 (hept, $^3J_{H-H}$ = 6.8 Hz, 1H, C***H***(CH$_3$)$_2$), 3.49 (hept, $^3J_{H-H}$ = 6.8 Hz, 1H, C***H***(CH$_3$)$_2$), 3.45 – 3.41 (m, 2H, MesNC***H$_2$***CH$_2$N(CH$_3$)$_2$), 3.25 (hept, $^3J_{H-H}$ = 6.8 Hz, 1H, C***H***(CH$_3$)$_2$), 3.25 (hept, $^3J_{H-H}$ = 6.8 Hz, 1H, C***H***(CH$_3$)$_2$), 3.24 (hept, $^3J_{H-H}$ = 6.8 Hz, 1H, C***H***(CH$_3$)$_2$), 3.24 (hept, $^3J_{H-H}$ = 6.8 Hz, 1H, C***H***(CH$_3$)$_2$), 2.90 (hept, $^3J_{H-H}$ = 6.8 Hz, 1H, C***H***(CH$_3$)$_2$), 2.75 (hept, $^3J_{H-H}$ = 6.8 Hz, 1H, C***H***(CH$_3$)$_2$), 2.30 – 2.26 (m, 2H, MesNCH$_2$C***H$_2$***NMe$_2$), 2.10 (s, 3H, 2x NC(C***H$_3$***)), 1.98 (s, 3H, 2x NC(C***H$_3$***)), 1.97 (s, 6H, Mes-C***H$_3$***), 1.93 (s, 3H, Mes-C***H$_3$***), 1.60 (s, 3H, N(C***H$_3$***)(CH$_3$)), 1.55 (s, 3H, N(CH$_3$)(C***H$_3$***)), 1.53 (d, $^3J_{H-H}$ = 6.7 Hz, 6H, (CH(C***H$_3$***)$_2$), 1.15 (d, $^3J_{H-H}$ = 6.8 Hz, 6H, (CH(C***H$_3$***)$_2$), 1.15 (d, $^3J_{H-H}$ = 6.8 Hz, 6H, (CH(C***H$_3$***)$_2$), 1.11 (d, $^3J_{H-H}$ = 6.8 Hz, 6H, (CH(C***H$_3$***)$_2$), 1.09 (d, $^3J_{H-H}$ = 6.9 Hz, 6H, (CH(C***H$_3$***)$_2$), 1.08 (d, $^3J_{H-H}$ = 6.9 Hz, 6H, (CH(C***H$_3$***)$_2$). Some C***H*** resonances are overlapping and cannot be observed.

^{13}C NMR (C_6D_6, 298 K, 101 MHz) δ: 169.2 (2x CH{***C***(CH$_3$)}$_2$), 148.7 (Ar-***C***), 145.7 (Ar-***C***), 141.1 (Ar-***C***), 141.4 (Ar-***C***), 134.1 (Ar-***C***), 133.6 (Ar-***C***H), 129.1 (Ar-***C***H), 127.0 (Ar-***C***H), 125.0 (Ar-***C***H), 95.7 (2x ***C***H{C(CH$_3$)}$_2$), 60.1 (MesN***C***H$_2$CH$_2$N(CH$_3$)$_2$), 60.4 (MesNCH$_2$CH$_2$N(***C***H$_3$)$_2$), 45.5 (MesNCH$_2$***C***H$_2$N(CH$_3$)$_2$), 28.8 (2x ***C***H(CH$_3$)$_2$), 27.9 (2x CH(***C***H$_3$)$_2$), 26.0 (2x CH(***C***H$_3$)$_2$), 25.2 (2x CH(***C***H$_3$)$_2$), 24.7 (2x CH(***C***H$_3$)$_2$), 24.5 (2x CH(***C***H$_3$)$_2$), 23.1 (2x NC(***C***H$_3$)), 20.9 (Mes-***C***H$_3$), 18.3 (Mes-***C***H$_3$). Some Ar***C*** resonances are overlapping and cannot be observed.

Anal. Calc. ($C_{42}H_{64}AlBN_4$): C, 76.11; H, 9.73; N, 8.45. Found: C, 76.66; H, 9.78; N, 8.16.

Preparation of 3b

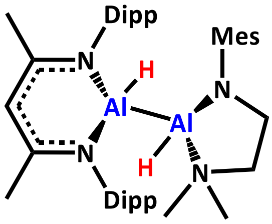

In a glovebox, **1** (40.0 mg, 0.090 mmol, 1 equiv.) and **2b** (21.1 mg, 0.090 mmol, 1 equiv.) were dissolved in C_6D_6 (1.5 mL) and transferred to a J. Young NMR tube. The mixture was allowed to react for 1h at 25 °C. A ^{1}H NMR spectrum was taken at this time point and showed the full conversion of starting materials to **3b**. The J. Young NMR tube was returned to the glovebox, the reaction mixture was decanted into a 20 mL scintillation vial and the solvent removed under vacuum, the crude product was then dissolved in 2 mL toluene. The solution was filtered through a glass fibre into a 4 mL vial and then stored in the glovebox freezer (–35 °C) for 4 days. Yellow crystals (**3b**) were successfully obtained. The filtrated crystals were washed with cold n-pentane (3 x 1mL) and then dried in vacuo. **Yield: 39 mg,** 0.058 mmol, 65%.

^{1}H NMR (C_6D_6, 298 K, 400 MHz): δ = 7.14 – 7.11 (m, 2H, ArC***H***), 7.09 – 7.05 (m, 3H, ArC***H***), 6.92 (m, 1H, ArC***H***), 6.82 (s, 2H, ArC***H***), 4.93 (s, 1H, C***H***{C(CH$_3$)}$_2$), 4.77 (bs, 1H, Al-***H***), 4.46 (bs, 1H, Al-***H***), 3.65 (hept, $^3J_{H-H}$ = 6.8 Hz, 1H, C***H***(CH$_3$)$_2$), 3.55 (hept, $^3J_{H-H}$ = 6.8 Hz, 1H, C***H***(CH$_3$)$_2$), 3.51 (hept, $^3J_{H-H}$ = 6.8 Hz, 1H, C***H***(CH$_3$)$_2$), 3.14 (hept, $^3J_{H-H}$ = 6.8 Hz, 1H, C***H***(CH$_3$)$_2$), 3.16 – 3.11 (m, 1H, MesNC***H₂***CH$_2$N(CH$_3$)$_2$), 2.74 – 2.66 (m, 1H, MesNC***H₂***CH$_2$N(CH$_3$)$_2$), 2.62 – 2.57 (m, 1H, MesNCH$_2$C***H₂***NMe$_2$), 2.33 (s, 3H, Mes-C***H₃***), 2.23 (s, 6H, Mes-C***H₃***), 1.82 (s, 3H, N(C***H₃***)(CH$_3$)), 1.68 – 1.64 (m, 1H, MesNCH$_2$C***H₂***NMe$_2$), 1.63 (s, 3H, N(CH$_3$)(C***H₃***)), 1.62 (d, $^3J_{H-H}$ = 6.8 Hz, 3H, (CH(C***H₃***)(CH$_3$)), 1.57 (d, $^3J_{H-H}$ = 6.8 Hz, 3H, (CH(C***H₃***)(CH$_3$)), 1.55 (s, 3H, NC(C***H₃***)), 1.53 (s, 3H, NC(C***H₃***)), 1.35 (d, $^3J_{H-H}$ = 6.7 Hz, 3H, (CH(C***H₃***)(CH$_3$)), 1.17 (d, $^3J_{H-H}$ = 6.9 Hz, 3H, (CH(C***H₃***)(CH$_3$)), 1.15 (d, $^3J_{H-H}$ = 6.9 Hz, 3H, (CH(C***H₃***)(CH$_3$)), 1.13 (d, $^3J_{H-H}$ = 6.9 Hz, 3H, (CH(C***H₃***)(CH$_3$)), 1.12 (d, $^3J_{H-H}$ = 6.9 Hz, 3H, (CH(C***H₃***)(CH$_3$)), 0.83 (d, $^3J_{H-H}$ = 6.7 Hz, 3H, (CH(C***H₃***)(CH$_3$)).

^{13}C NMR (C_6D_6, 298 K, 101 MHz) δ: 169.9 (CH{***C***(CH$_3$)}$_2$), 168.5 (CH{***C***(CH$_3$)}$_2$), 147.9 (Ar-***C***), 145.8 (Ar-***C***), 144.9 (Ar-***C***), 143.7 (Ar-***C***), 143.4 (Ar-***C***), 141.5 (Ar-***C***), 141.3 (Ar-***C***), 136.5 (Ar-***C***H), 130.1 (Ar-CH), 129.1 (Ar-***C***H), 126.5 (Ar-***C***H), 126.0 (Ar-***C***H), 124.4 (Ar-***C***H), 124.3 (Ar-***C***H), 124.1 (Ar-***C***H), 123.5 (Ar-***C***H), 96.7 (***C***H{C(CH$_3$)), 61.3 (MesN***C***H$_2$CH$_2$N(CH$_3$)(CH$_3$)), 47.9 (MesNCH$_2$***C***H$_2$N(CH$_3$)(CH$_3$)), 45.8 (MesNCH$_2$CH$_2$N(***C***H$_3$)(CH$_3$)), 43.8 (MesNCH$_2$CH$_2$N(CH$_3$)(***C***H$_3$)), 29.5 (***C***H(CH$_3$)(CH$_3$)) , 28.8 (***C***H(CH$_3$)(CH$_3$)), 27.7 (***C***H(CH$_3$)(CH$_3$)), 27.3 (***C***H(CH$_3$)(CH$_3$)), 25.9 (CH(***C***H$_3$)(CH$_3$)), 25.6 (CH(***C***H$_3$)(CH$_3$)), 24.6 (CH(***C***H$_3$)(CH$_3$)), 24.5 (CH(***C***H$_3$)(CH$_3$)), 24.4 (CH(***C***H$_3$)(CH$_3$)), 24.3 (CH(***C***H$_3$)(CH$_3$)), 23.9 (CH(***C***H$_3$)(CH$_3$)), 23.7 (CH(***C***H$_3$)(CH$_3$)), 23.1 (NC(***C***H$_3$)), 23.0 (NC(***C***H$_3$)), 20.8 (Mes-***C***H$_3$), 19.6 (Mes-***C***H$_3$).

Anal. Calc. ($C_{42}H_{64}Al_2N_4$): C, 74.30; H, 9.50; N, 8.25. Found: C, 74.67; H, 7.62; N, 8.07.

Preparation of 4

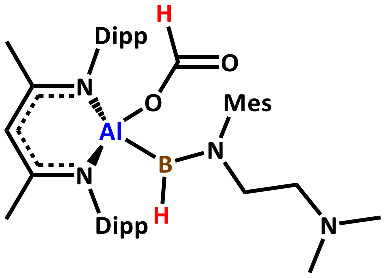

Method One: In a glovebox, **3a/3a'** (20.0 mg, 0.030 mmol, 1 equiv.) was dissolved in C_6D_6 (0.5 mL) and transferred to a J. Young NMR tube. The reaction mixture was frozen at –78 °C and evacuated, CO_2 gas (~1.5 bar, ~0.14 mmol) was introduced into the NMR tube. The reaction mixture was stored at 25 °C for 14 hours. A ^{1}H NMR spectrum was taken at this time point and showed the full conversion of starting material to **4**. The J. Young NMR tube was returned to the glovebox, the reaction mixture was decanted into a 20 mL scintillation vial and solvent removed under vacuum and the crude was dissolved in 1 mL toluene/n-pentane (1:1 v: v) mixture. The solution was filtered through a glass fibre into a 4 mL vial and then stored in the glovebox freezer (–35 °C) for 3 days. Bright yellow crystals (**4**) were successfully obtained. The filtrated crystals were washed with cold n-pentane (3 x 1mL) and then dried in vacuo. **Yield: 13 mg,** 0.018 mmol, 60%.

Method Two: In a glovebox, **1** (20.0 mg, 0.045 mmol, 1 eq) and **2a** (9.8 mg, 0.045 mmol, 1 eq) were dissolved in C_6D_6 (0.5 mL) and transferred to a J. Young NMR tube. The NMR tube was cooled to liq. N_2 temperature and evacuated, CO_2 gas (1.0 bar, ~0.1 mmol) was introduced into the NMR tube and the reaction mixture was stored at 25 °C for 14 hours. A ^{1}H NMR spectrum was taken at this time point and showed the full conversion of starting material to **4**. The J. Young NMR tube was returned to the glovebox, the reaction mixture was decanted into a 20 mL scintillation vial and the solvent removed under vacuum. The crude product was dissolved in 1 mL Et_2O/n-pentane (1:2 v: v) mixture. The solution was filtered through a glass fibre into a 4 mL vial and stored in the glovebox freezer (–35 °C) for 3 days. Bright yellow crystals (**4**) were successfully obtained. The filtrated crystals were washed with cold n-pentane (3 x 1mL) and then dried in vacuo. **Yield: 16 mg,** 0.023 mmol, 50%.

^{1}H NMR (C_6D_6, 298 K, 400 MHz) δ: 8.65 (s, 1H, C***H***O_2), 7.15 – 7.12 (m, 2H, ArC***H***), 7.08 – 7.05 (m, 2H, ArC***H***), 7.04 – 6.97 (m, 2H, ArC***H***), 6.61 (s, 2H, ArC***H***), 5.19 (s, 1H, , C***H***{C(CH$_3$)}$_2$), 4.99 (bs, 1H, B***H***), 3.74 – 3.66 (m, 2H, MesNC***H₂***CH$_2$N(CH$_3$)$_2$), 3.42 (hept, $^3J_{H-H}$ = 7.0 Hz, 2H, C***H***(CH$_3$)$_2$), 3.39 (hept, $^3J_{H-H}$ = 6.9 Hz, 2H, C***H***(CH$_3$)$_2$), 2.36 – 2.28 (m, 2H, MesNCH$_2$C***H₂***N(CH$_3$)$_2$), 2.01 (s, 6H, 2x NC(C***H₃***)), 1.97 (s, 3H, Mes-C***H₃***)), 1.82 (s, 6H, Mes-C***H₃***), 1.62 (s, 6H, N(C***H₃***)$_2$), 1.45 (d, $^3J_{H-H}$ = 6.7 Hz, 6H, (CH(C***H₃***)$_2$), 1.23 (d, $^3J_{H-H}$ = 6.7 Hz, 6H, (CH(C***H₃***)$_2$), 1.21 (d, $^3J_{H-H}$ = 6.6 Hz, 6H, (CH(C***H₃***)$_2$), 1.02 (d, $^3J_{H-H}$ = 6.8 Hz, 6H, (CH(C***H₃***)$_2$).

^{13}C NMR (C_6D_6, 298 K, 101 MHz) δ: 170.2 (CH{***C***(CH_3)}$_2$), 162.0 (***CH***O_2), 148.7 (Ar-***C***), 145.7 (Ar-***C***), 143.6 (Ar-***C***), 140.7 (Ar-***C***), 133.8 (Ar-***C***H), 132.4 (Ar-***C***H), 129.2 (Ar-***C***H), 127.3 (Ar-***C***H), 124.8 (Ar-***C***H), 124.1 (Ar-***C***H), 100.2 (2x ***C***H{C(CH_3)}$_2$), 59.7 (MesN***C***$H_2CH_2N(CH_3)_2$), 58.1 (MesNCH_2CH_2N(***C***H_3)$_2$) , 45.9 (MesNCH_2***C***$H_2N(CH_3)_2$), 29.4 (***C***H(CH_3)$_2$), 27.3 (***C***H(CH_3)$_2$), 24.9 (CH(***C***H_3)$_2$), 24.4 (CH(***C***H_3)$_2$), 23.9 (CH(***C***H_3)$_2$), 23.3 (CH(***C***H_3)$_2$), 20.71 (Mes-***C***H_3), 18.61 (Mes-***C***H_3).

^{11}B NMR (C_6D_6, 298 K, 128 MHz) δ: 25.3 (bs, ***B***H)

Anal. Calc. ($C_{43}H_{64}AlBN_4O_4$): C, 73.07; H, 9.13; N, 7.93. Found: C, 73.24; H, 9.10; N, 7.28.

Preparation of 5

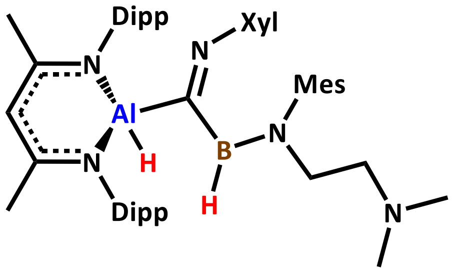

Method One: In a glovebox, **3a/3a'** (40.0 mg, 0.060 mmol, 1 equiv.) and 2-isocyano-1,3-dimethylbenzene (7.9 mg, 0.06 mmol, 1 equiv.) were dissolved in C_6D_6 (1.0 mL) and transferred to a J. Young NMR tube. The reaction mixture was stored for 5 mins at 25 °C. A ^{1}H NMR spectrum was taken at this time point and showed the full conversion of starting material to **5**. The J. Young NMR tube was returned to the glovebox, the reaction mixture was decanted to a 20 mL scintillation vial, the solvent was removed under vacuum and the crude product dissolved in 1 mL Et_2O/n-pentane (1:2 v: v) mixture. The solution was filtered through a glass fibre into a 4 mL vial and then stored in the glovebox freezer (–35 °C) for 3 days. Bright yellow crystals (**5**) were successfully obtained. The filtrated crystals were washed with cold n-pentane (3 x 1mL) and then dried in vacuo. **Yield: 34 mg,** 0.042 mmol, 70%.

Method Two: In a glovebox, **1** (40.0 mg, 0.090 mmol, 1 equiv.) and **2a** (19.6 mg, 0.090 mmol, 1 equiv.) were dissolved in C_6D_6 (0.5 mL) and transferred to a J. Young NMR tube. 2-isocyano-1,3-dimethylbenzene (7.9 mg, 0.06 mmol, 1 equiv.) was dissolved in C_6D_6 (0.5 mL) and then added to the reaction mixture. The reaction mixture was stored for 5 mins at 25 °C. A ^{1}H NMR spectrum was taken at this time point and showed the full conversion of the starting materials to **5**. The J. Young NMR tube was returned to the glovebox, the reaction mixture was decanted into a 20 mL scintillation vial and the solvent removed under vacuum. The crude product was dissolved in a 1 mL Et_2O/n-pentane (1:2 v: v) mixture. The solution was filtered through a glass fibre into a 4 mL vial and then stored in the glovebox freezer (–35 °C) for 3 days. Bright yellow crystals (**5**) were successfully obtained. The filtrated crystals were washed with cold n-pentane (3 x 1mL) and then dried in vacuo. **Yield: 36 mg,** 0.045 mmol, 50%.

^{1}H NMR (C_6D_6, 298 K, 400 MHz) δ: 7.13 – 7.09 (m, 4H, ArC***H***), 7.08 – 7.03 (m, 2H, ArC***H***), 6.88 (d, $^3J_{H-H}$ = 7.4 Hz, 2H, ArC***H***), 6.80 (dd, $^3J_{H-H}$ = 8.2, 6.6 Hz, 1H, ArC***H***), 6.67 (s, 2H, Mes-***H***), 5.02 (s, 1H, C***H***{C(CH$_3$)}$_2$), 4.96 (s, 1H, B-***H***), 4.40 (s, 1H, Al-***H***), 3.70 (hept, $^3J_{H-H}$ = 7.0 Hz, 2H, C***H***(CH$_3$)$_2$), 3.40 (hept, $^3J_{H-H}$ = 6.8 Hz, 2H, C***H***(CH$_3$)$_2$), 3.05 – 2.95 (m, 2H, MesNC***H$_2$***CH$_2$NMe$_2$), 2.04 (s, 3H, Mes-C***H$_3$***), 1.89 (s, 6H, Xyl-C***H$_3$***), 1.81 (s, 6H, Mes-C***H$_3$***), 1.74 (s, 6H, N(C***H$_3$***)$_2$), 1.70 – 1.63 (m, 2H, MesNCH$_2$C***H$_2$***NMe$_2$), 1.55 (s, 6H, N(C***H$_3$***)$_2$), 1.39 (d, $^3J_{H-H}$ = 6.7 Hz, 6H, (CH(C***H$_3$***)$_2$)), 1.36 (d, $^3J_{H-H}$ = 6.8 Hz, 6H, (CH(C***H$_3$***)$_2$)), 1.20 (d, $^3J_{H-H}$ = 6.8 Hz, 6H, (CH(C***H$_3$***)$_2$)), 1.12 (d, $^3J_{H-H}$ = 6.9 Hz, 6H, (CH(C***H$_3$***)$_2$)).

Since compound **5** is not stable for a long time even under low temperature conditions, ^{13}C NMR spectra could not be collected and CHN results do not match the expected values.

Preparation of 6

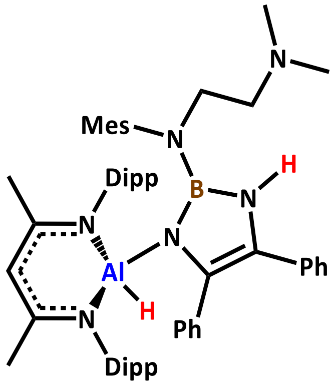

Method One: In a glovebox, **3a/3a'** (40.0 mg, 0.060 mmol, 1 equiv.) and benzonitrile (12.4 mg, 0.12 mmol, 2 equiv.) were dissolved in C_6D_6 (0.6 mL) and transferred to a J. Young NMR tube. The reaction mixture was stored for 12 hours at 25 °C. A ^{1}H NMR spectrum was taken at this time point and showed the full conversion of the starting material to **6**. The J. Young NMR tube was returned to the glovebox, the reaction mixture was decanted into a 20 mL scintillation vial and the solvent removed under vacuum and the crude product disolved in a 1 mL Et_2O/n-pentane (1:2 v: v) mixture. The solution was filtered through a glass fibre into a 4 mL vial and then stored in the glovebox freezer (–35 °C) for 3 days. Bright yellow crystals (**6**) were successfully obtained. The filtrated crystals were washed with cold n-pentane (3 x 1mL) and then dried in vacuo. **Yield: 31 mg,** 0.036 mmol, 60%.

Method Two: In a glovebox, **1** (40.0 mg, 0.090 mmol, 1 eq) and **2a** (19.6 mg, 0.090 mmol, 1 equiv.) were dissolved in C_6D_6 (0.5 mL) and transferred to a J. Young NMR tube. benzonitrile (12.4 mg, 0.12 mmol, 2 eq) was dissolved in C_6D_6 (0.5 mL) and addedto the reaction mixture. The reaction mixture was stored for 12 hours at 25 °C. A ^{1}H NMR spectrum was taken at this time point and showed the full conversion of starting material to **6**. The J. Young NMR tube was returned to the glovebox, the reaction mixture was decanted into a 20 mL scintillation vial, the solvent was removed under vacuum and the crude mixture dissolved in a 1 mL Et_2O/n-pentane (1:2 v: v) mixture. The solution was filtered through a glass fibre into a 4 mL vial and then stored in the glovebox freezer (–35 °C) for 3 days. Bright yellow crystals (**3a**) were successfully obtained. The filtrated crystals were washed with cold n-pentane (3 x 1mL) and then dried in vacuo. **Yield: 39 mg,** 0.045 mmol, 50%.

^{1}H NMR (C_6D_6, 298 K, 400 MHz) δ: 7.68 – 7.64 (m, 2H, ArC***H***), 7.21 – 7.16 (m, 2H, ArC***H***), 7.13 – 7.11 (m, 6H, ArC***H***), 7.10 – 7.05 (m, 2H, ArC***H***), 7.02 – 6.97 (m, 1H, ArC***H***), 6.92 (t, $^3J_{H\text{-}H}$ = 7.5 Hz, 2H, ArC***H***), 6.86 – 6.81 (m, 1H, ArC***H***), 6.73 (s, 2H, ArC***H***), 5.32 (bs, 1H, N***H***), 4.99 (s, 1H, C***H***{C(CH$_3$)}$_2$), 4.15 (s, 1H, Al***H***), 3.40 –3.35 (m, 2H, MesNC***H$_2$***CH$_2$N(CH$_3$)$_2$), 3.29 (hept, $^3J_{H\text{-}H}$ = 7.0 Hz, 4H, C***H***(CH$_3$)$_2$), 2.31 – 2.87 (m, 2H, MesNCH$_2$C***H$_2$***N(CH$_3$)$_2$), 2.10 (s, 3H, Mes-C***H$_3$***), 2.08 (s, 6H, 2x NC(C***H$_3$***)), 1.81 (s, 6H, Mes-C***H$_3$***), 1.46 (s, 6H, N(C***H$_3$***)$_2$), 1.43 (d, $^3J_{H\text{-}H}$ = 6.7 Hz, 6H, (CH(C***H$_3$***)$_2$), 1.22 (d, $^3J_{H\text{-}H}$ = 6.6 Hz, 6H, (CH(C***H$_3$***)$_2$), 1.15 (d d, $^3J_{H\text{-}H}$ = 6.9 Hz, 6H, (CH(C***H$_3$***)$_2$), 1.01 (d, $^3J_{H\text{-}H}$ = 6.8 Hz, 6H, (CH(C***H$_3$***)$_2$).

^{13}C NMR (C_6D_6, 298 K, 101 MHz) δ: 170.7 (CH{***C***(CH_3)}$_2$), 145.7 (Ar-***C***), 144.0 (Ar-***C***), 140.6 (Ar-***C***), 139.1 (Ar-***C***), 137.0 (Ar-***C***), 136.6 (BN***C***(Ph)C(Ph)NH), 134.4 (BNC(Ph)***C***(Ph)NH), 131.0 (Ar-***C***H), 129.5 (Ar-***C***H), 129.5 (Ar-***C***H), 128.1 (Ar-***C***H), 127.9 (Ar-***C***H), 127.4 (Ar-***C***H), 127.2 (Ar-***C***H), 124.8 (Ar-***C***H), 124.7 (Ar-***C***H), 124.4 (Ar-***C***H), 100.5 (***C***H{C(CH_3)}$_2$), 57.9 (MesN***C***H_2CH_2N(CH_3)$_2$), 51.4 (MesNCH_2CH_2N(***C***H_3)$_2$), 45.5 (MesNCH_2***C***H_2N(CH_3)$_2$), 28.5 (2x ***C***H(CH_3)$_2$), 27.9 (2x ***C***H(CH_3)$_2$), 25.3 (2x CH(***C***H_3)$_2$), 25.0 (2x CH(***C***H_3)$_2$), 24.5 (2x CH(***C***H_3)$_2$), 24.1 (2x CH(***C***H_3)$_2$), 23.6 (2x NC(***C***H_3)), 20.6 (Mes-***C***H_3), 18.53 (Mes-***C***H_3). Some Ar***C*** resonances are overlapping and cannot be observed.

^{11}B NMR (C_6D_6, 298 K, 128 MHz) δ: 28.4 (bs, ***B***H)

Anal. Calc. ($C_{56}H_{74}AlBN_6$): C, 77.40; H, 8.58; N, 9.67. Found: C, 76.57; H, 8.11; N, 9.81. The CHN results match the expected values for **6-(OEt_2)$_{0.67}$**.

3) Single Crystal X-ray Diffraction Data

Table S1. Crystal Data, Data Collection and Refinement Parameters for the structures of **3a**, **3b**, **4**, **5**.

**data**	**3a**	**3b**	**4**	**5**
**formula**	$C_{42}H_{64}AlBN_4$	$C_{42}H_{64}Al_2N_4$	$C_{43}H_{64}AlBN_4O_2$	$C_{51}H_{73}AlBN_5$
**solvent**	—	—	—	—
**formula weight**	662.76	678.93	706.77	793.93
**colour, habit**	colourless blocks	yellow tabular needles	colourless platy	yellow blocks
**temperature / K**	173	173	173	173
**crystal system**	triclinic	monoclinic	triclinic	triclinic
**space group**	*P*–1 (no. 2)	$P2_1/n$ (no. 14)	*P*–1 (no. 2)	*P*–1 (no. 2)
***a* / Å**	10.3732(5)	13.63125(14)	10.6201(8)	11.1773(8)
***b* / Å**	12.2046(6)	20.16105(19)	12.1477(11)	13.0784(8)
***c* / Å**	18.1561(7)	15.31570(14)	18.1465(11)	18.2008(12)
**α / deg**	91.465(4)	90	91.246(6)	75.079(6)
**β / deg**	106.113(4)	99.9088(9)	104.782(6)	72.532(6)
**γ / deg**	107.935(4)	90	107.331(7)	74.007(6)
**V / Å^3**	2085.43(18)	4146.28(7)	2148.7(3)	2395.0(3)
Z	2	4	2	2
**D_c / g cm^{-3}**	1.055	1.088	1.092	1.101
**radiation used**	Cu-Kα	Cu-Kα	Cu-Kα	Cu-Kα
**μ / mm^{-1}**	0.649	0.863	0.695	0.648
**no. of unique reflns**				
**measured (R_{int})**	7979 (0.0313)	8227 (0.0411)	8198 (0.0586)	9081 (0.0392)
**obs, $\|F_o\| > 4\sigma(\|F_o\|)$**	6097	6642	5145	6135
**completeness (%)** [a]	98.4	99.9	98.2	97.8
**no. of variables**	457	467	490	548
**R_1(obs), wR_2(all)** [b]	0.0446, 0.1282	0.0408, 0.1150	0.0540, 0.1398	0.0497, 0.1365
**CCDC code**	2363830	2363834	2363832	2363831

[a] Completeness to 0.84 Å resolution. [b] $R_1 = \Sigma||F_o| - |F_c||/\Sigma|F_o|$; $wR_2 = \{\Sigma[w(F_o^2 - F_c^2)^2] / \Sigma[w(F_o^2)^2]\}^{1/2}$; $w^{-1} = \sigma^2(F_o^2) + (aP)^2 + bP$. [c] The complex has crystallographic C_2 symmetry. [d] There are two crystallographically independent molecules.

Table S1. Crystal Data, Data Collection and Refinement Parameters for the structures of **6**.

**data**	**6**
**formula**	$C_{56}H_{74}AlBN_6$
**solvent**	$0.75(C_4H_{10}O)$
**formula weight**	924.59
**colour, habit**	yellow plates
**temperature / K**	173
**crystal system**	triclinic
**space group**	*P*–1 (no. 2)
a **/ Å**	11.7887(5)
b **/ Å**	12.6943(6)
c **/ Å**	19.3282(11)
**α / deg**	94.912(4)
**β / deg**	91.626(4)
**γ / deg**	103.385(4)
V **/ Å³**	2800.1(2)
Z	2
D_c **/ g cm⁻³**	1.097
**radiation used**	Cu-Kα
**μ / mm⁻¹**	0.635
**no. of unique reflns**	
**measured (**R_{int}**)**	10641 (0.0363)
**obs, |**F_o**| > 4σ(|**F_o**|)**	6169
**completeness (%)** [a]	98.0
**no. of variables**	722
R_1**(obs),** wR_2**(all)** [b]	0.0688, 0.2152
**CCDC code**	2363833

Table S1 and Table S2 provide2 a summary of the crystallographic data for the structures of **3a**, **3b**, **4**, **5** and **6**. Data were collected using an Agilent Xcalibur PX Ultra A diffractometer, and the structures were solved and refined using the OLEX2,[S4] SHELXTL[S5] and SHELX-2013[S6] program systems. The absolute structure of **3c** was determined by use of the Flack parameter [*x* = 0.070(19)]. CCDC 2363830 to 2363837.

The X-ray crystal structure of 3a

The Al1–H hydrogen atom in the structure of **3a** was located from a Δ*F* map and refined freely. The B30–H hydrogen atom was located from a Δ*F* map and refined freely subject to a B–H distance constraint of 1.12 Å.

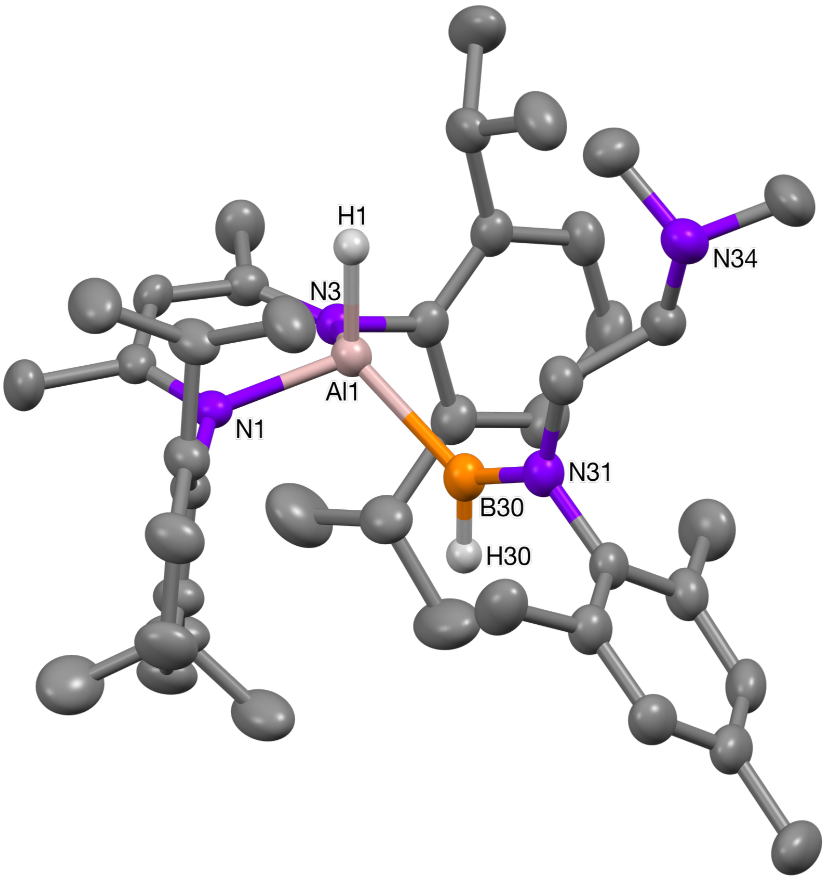

Fig. S1 The crystal structure of **3a** (50% probability ellipsoids).

The X-ray crystal structure of 3b

The C23-bound isopropyl group in the structure of **3b** was found to be disordered. Two orientations were identified of *ca*. 93 and 7% occupancy, their geometries were optimised, the thermal parameters of adjacent atoms were restrained to be similar, and only the non-hydrogen atoms of the major occupancy orientation were refined anisotropically (those of the minor occupancy orientation were refined isotropically). The Al1–H and Al2–H hydrogen atoms were both located from Δ*F* maps and refined freely.

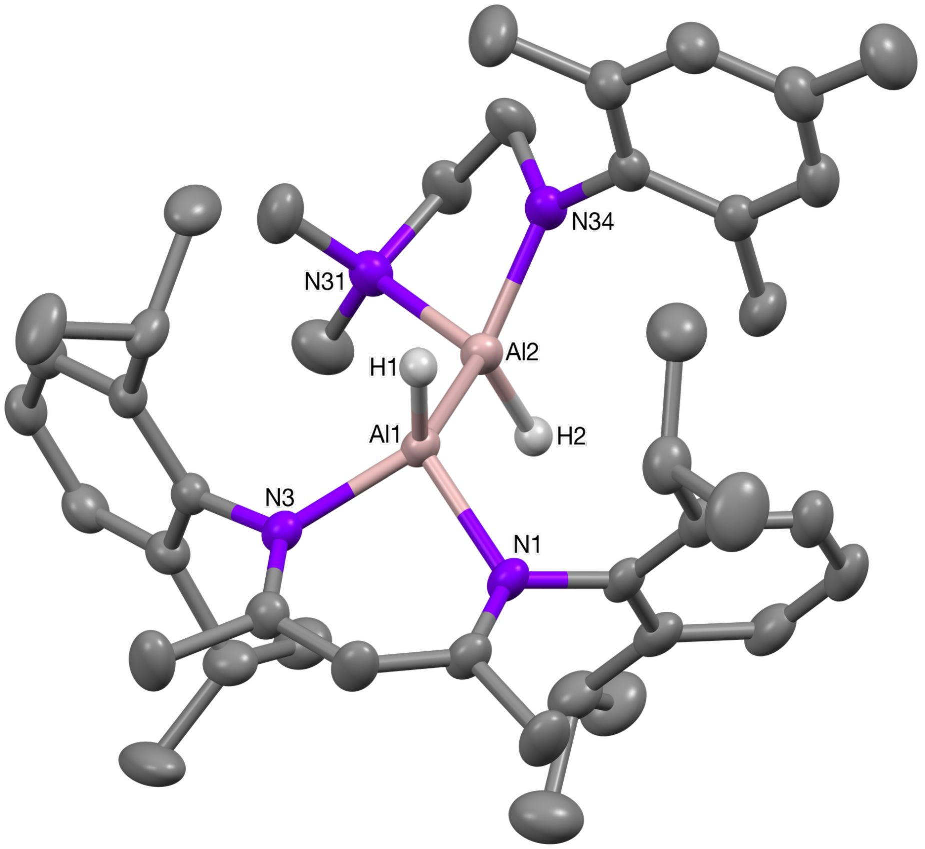

Fig. S5 The crystal structure of **3b** (50% probability ellipsoids).

The X-ray crystal structure of 4

The C7-bound isopropyl group in the structure of **4** was found to be disordered. Two orientations were identified of *ca*. 68 and 32% occupancy, their geometries were optimised, the thermal parameters of adjacent atoms were restrained to be similar, and only the non-hydrogen atoms of the major occupancy orientation were refined anisotropically (those of the minor occupancy orientation were refined isotropically). The B30–H hydrogen atom was located from a Δ*F* map and refined freely subject to a B–H distance constraint of 1.12 Å.

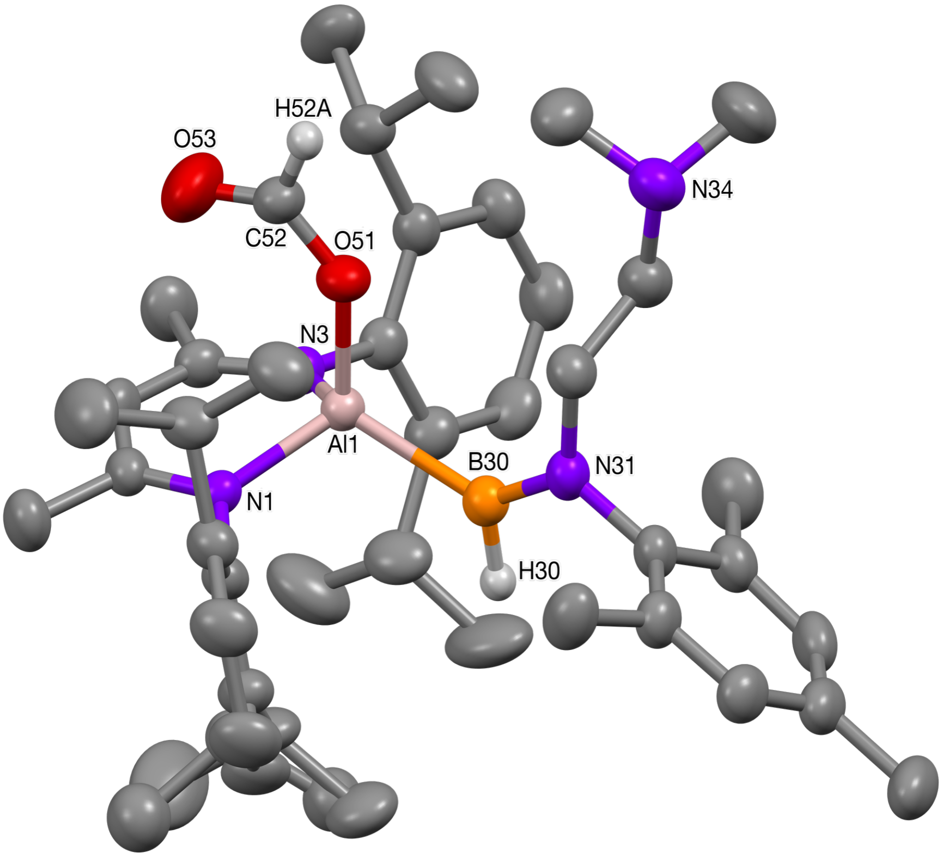

Fig. S3 The crystal structure of **4** (50% probability ellipsoids).

The X-ray crystal structure of 5

The Al1–H hydrogen atom in the structure of **5** was located from a Δ*F* map and refined freely. The B31–H hydrogen atom was located from a Δ*F* map and refined freely subject to a B–H distance constraint of 1.12 Å.

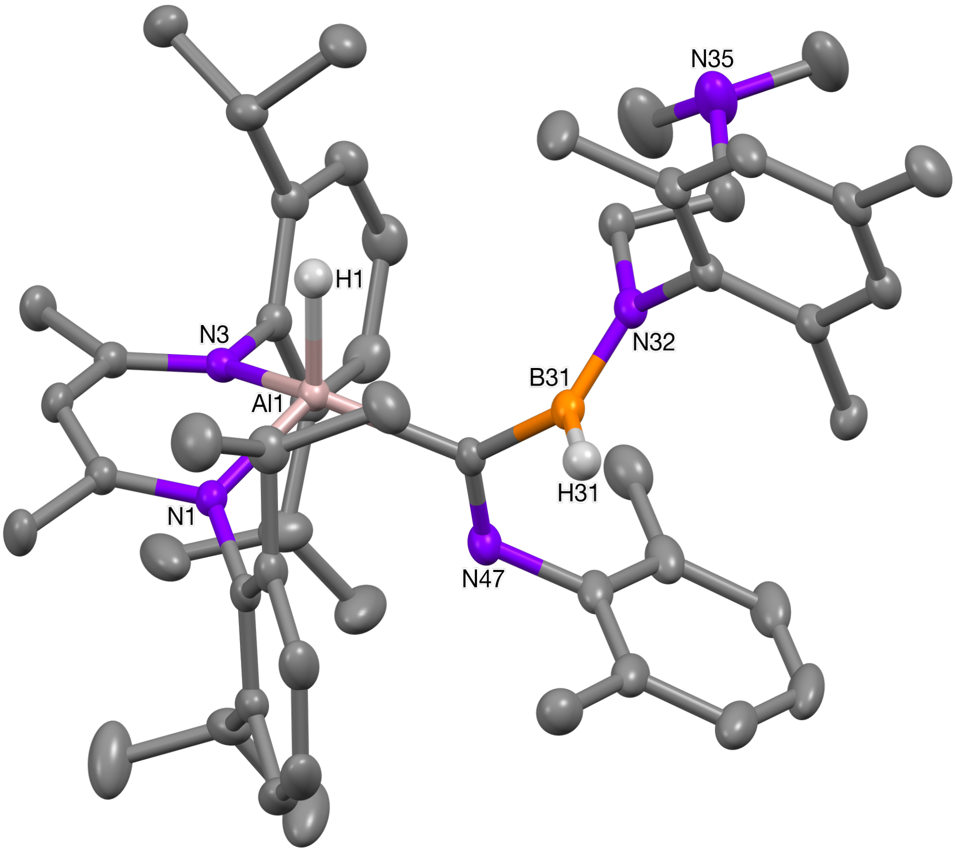

Fig. S2 The crystal structure of **5** (30% probability ellipsoids).

The X-ray crystal structure of 6

Both the AlHNacNac unit and the N47-based $N(CH_2)NMe_2$ portion of the boron-containing ligand in the structure of **6** were found to be disordered, and in each case two orientations were identified, of *ca*. 69:31 and 76:24% occupancy respectively. The geometries of each pair of orientations were optimised, the thermal parameters of adjacent atoms were restrained to be similar, and only the non-hydrogen atoms of the major occupancy orientations were refined anisotropically (those of the minor occupancy orientations were refined isotropically). The Al–H hydrogen atoms for each orientation were located from ΔF maps and refined freely, but obviously should be treated with more than the usual caution. The N33–H hydrogen atom was located from a ΔF map and refined freely subject to an N–H distance constraint of 0.90 Å. The included solvent was found to be highly disordered, and the best approach to handling this diffuse electron density was found to be the SQUEEZE routine of PLATON.[S7] This suggested a total of 56 electrons per unit cell, equivalent to 28 electrons per asymmetric unit. Before the use of SQUEEZE the solvent was unclear so the most recently used solvent (diethylether, $C_4H_{10}O$, 42 electrons) was presumed, and 0.75 diethylether molecules corresponds to 31.5 electrons so this was used as the solvent present. As a result, the atom list for the asymmetric unit is low by $0.75(C_4H_{10}O) = C_3H_{7.5}O_{0.75}$ (and that for the unit cell low by $C_6H_{15}O_{1.5}$) compared to what is actually presumed to be present.

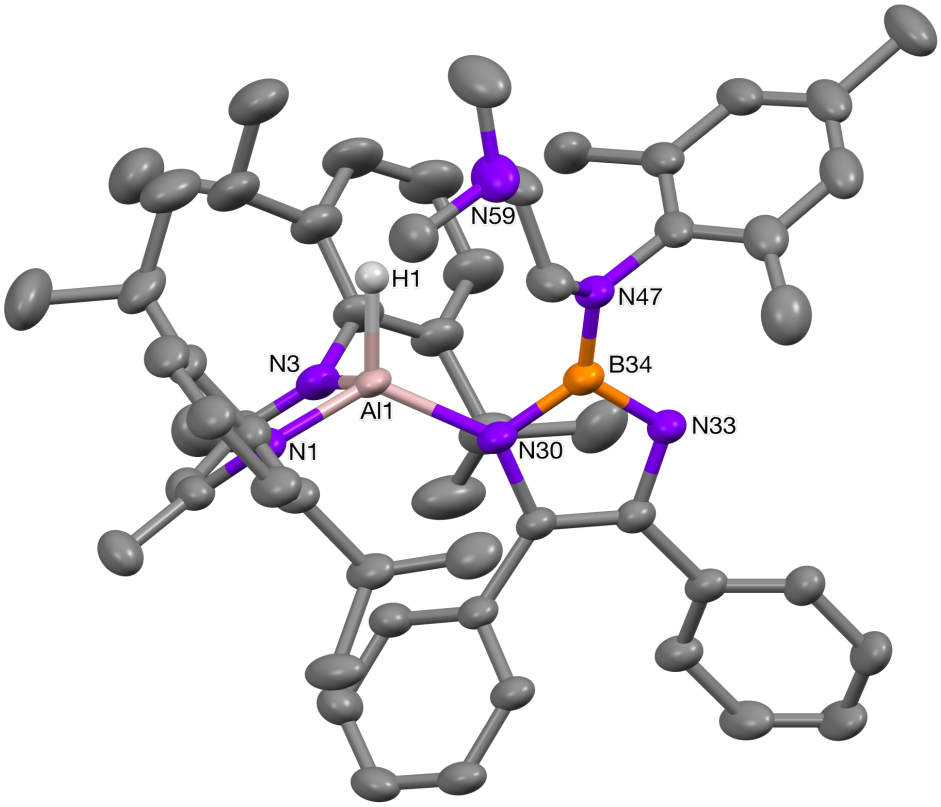

Fig. S4 The crystal structure of **6** (30% probability ellipsoids).

4) DFT Studies

4.1. Computational methods

DFT calculations were performed using Gaussian 09 (Revision D.01) using an ultrafine integration grid (int=ultrafine).[S8] Geometry optimisations and frequency calculations were performed using the B3PW91 density functionals including solvent and dispersion corrections (D3) with SDDAll (Al), 6-31G** (C, H) and 6-311+G* (B, N) basis set. Frequency analyses for all stationary points were performed using the enhanced criteria to confirm the nature of the structures as either minima (no imaginary frequency) or transition states (only one imaginary frequency). The electronic energies of the optimised geometries were calculated using the B3PW91 functional including solvent and dispersion corrections (D3) with 6-311+G** basis sets for all atoms with solvent corrections (PCM, benzene, ε = 2.2706). The Gibbs free energy correction from the frequency calculation was added to this electronic energy to generate Gibbs free energy values for the calculated stationary points.

Intrinsic reaction coordinate (IRC) calculations were used to connect transition states and minima located on the potential energy surface allowing a full energy profile (calculated at 298.15 K, 1 atm.) of the reaction to be constructed.

NBO analysis was performed at the B3PW91/6-311+G** level for all atoms with dispersion corrections (D3) and solvent corrections (PCM, benzene, ε = 2.2706) by using NBO 6.0.[S9] QTAIM analysis was conducted using the AIMAll software. [S10-S11]

4.2. Calculated Reaction Pathway

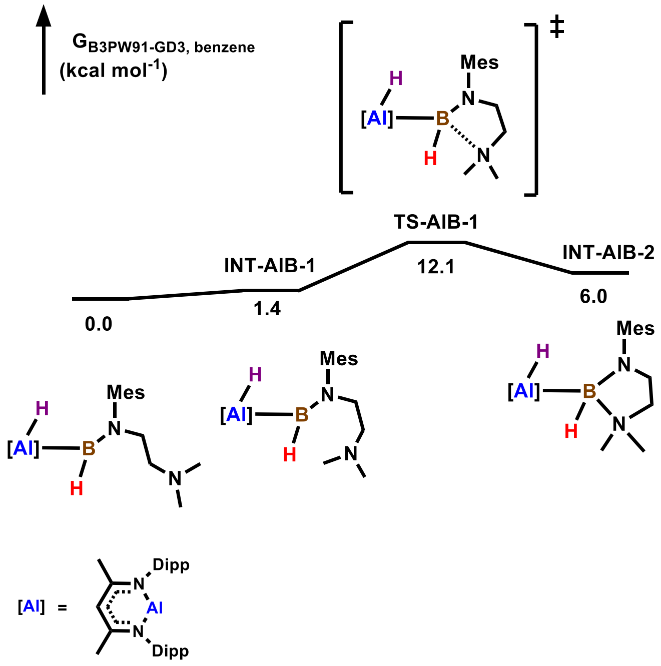

Figure S. Proposed pathway for reversible ligand coordination based on DFT calculations G09: B3PW91-D3 / 6-311+G**/ PCM (benzene) // B3PW91-D3 / 6-31G** / 6-311+G* / SDDAll (Al). Free Energy kcal mol^{-1}.

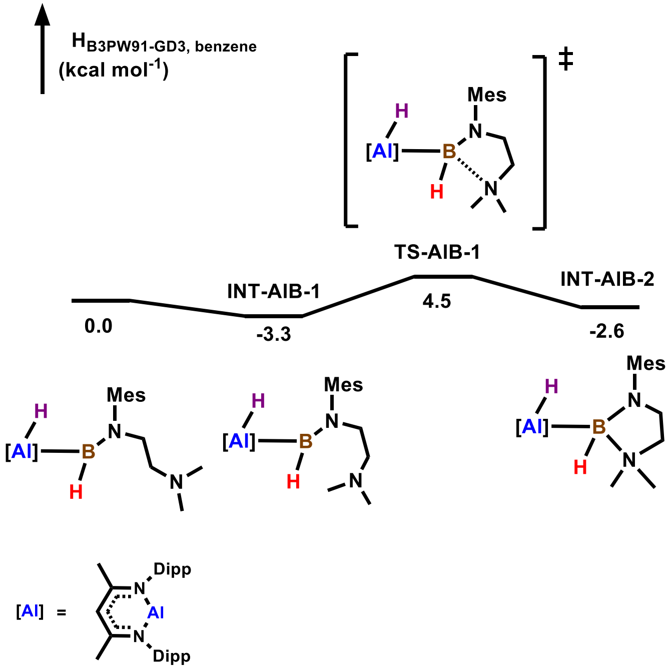

Figure S. Proposed pathway for reversible ligand coordination based on DFT calculations G09: B3PW91-D3 / 6-311+G**/ PCM (benzene) // B3PW91-D3 / 6-31G** / 6-311+G* / SDDAll (Al). Enthalpy kcal mol^{-1}.

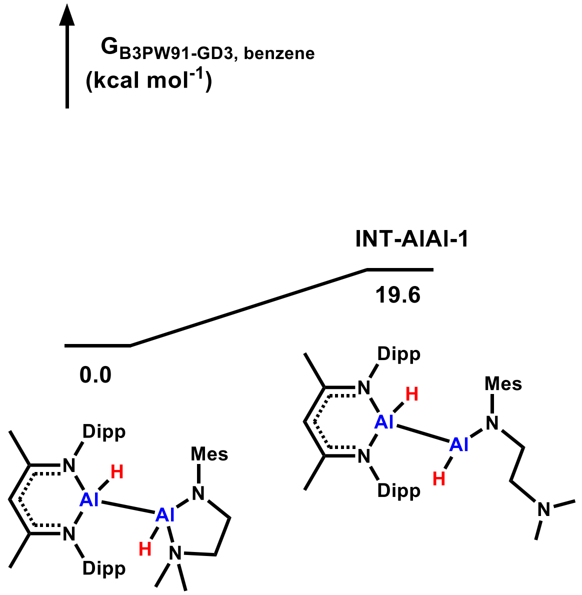

Figure S. Proposed pathway for reversible ligand coordination based on DFT calculations G09: B3PW91-D3 / 6-311+G**/ PCM (benzene) // B3PW91-D3 / 6-31G** / 6-311+G* / SDDAll (Al). Enthalpy kcal mol^{-1}.

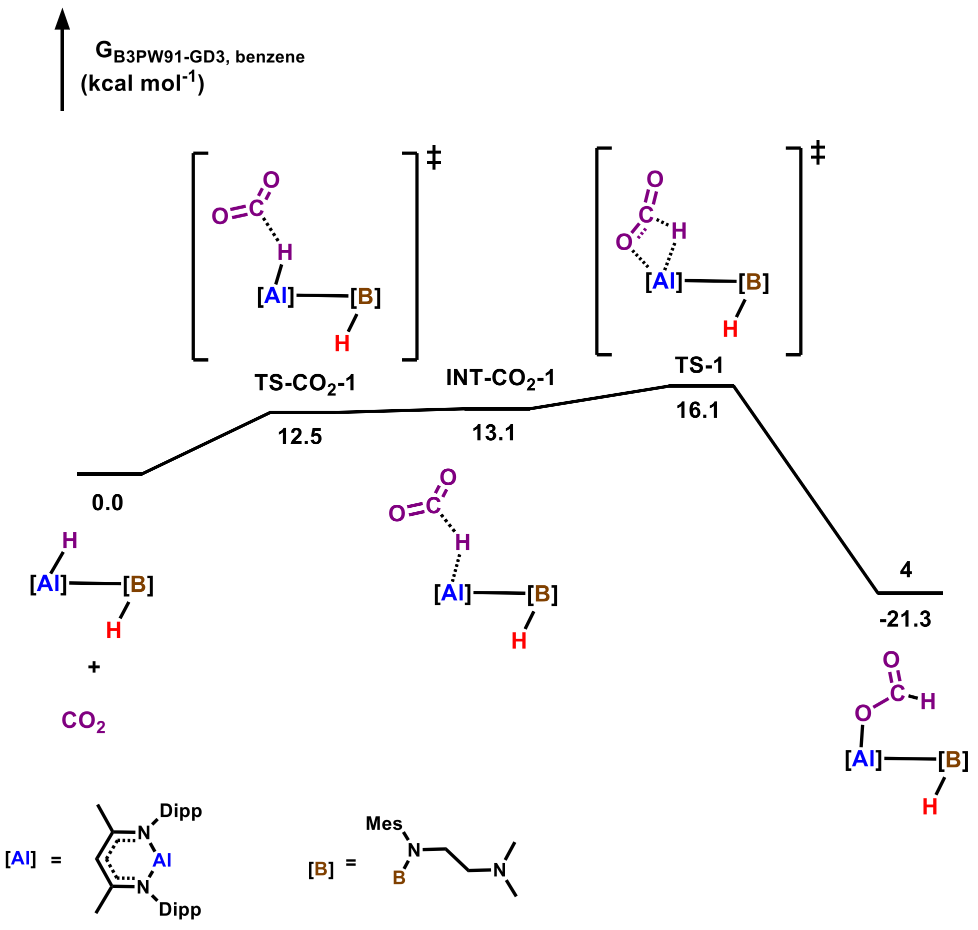

Figure S. Proposed pathway for the reaction of CO_2 reacted with **3a** based on DFT calculations G09: B3PW91-D3 / 6-311+G**/ PCM (benzene) // B3PW91-D3 / 6-31G** / 6-311+G* / SDDAll (Al). Free Energy kcal mol^{-1}.

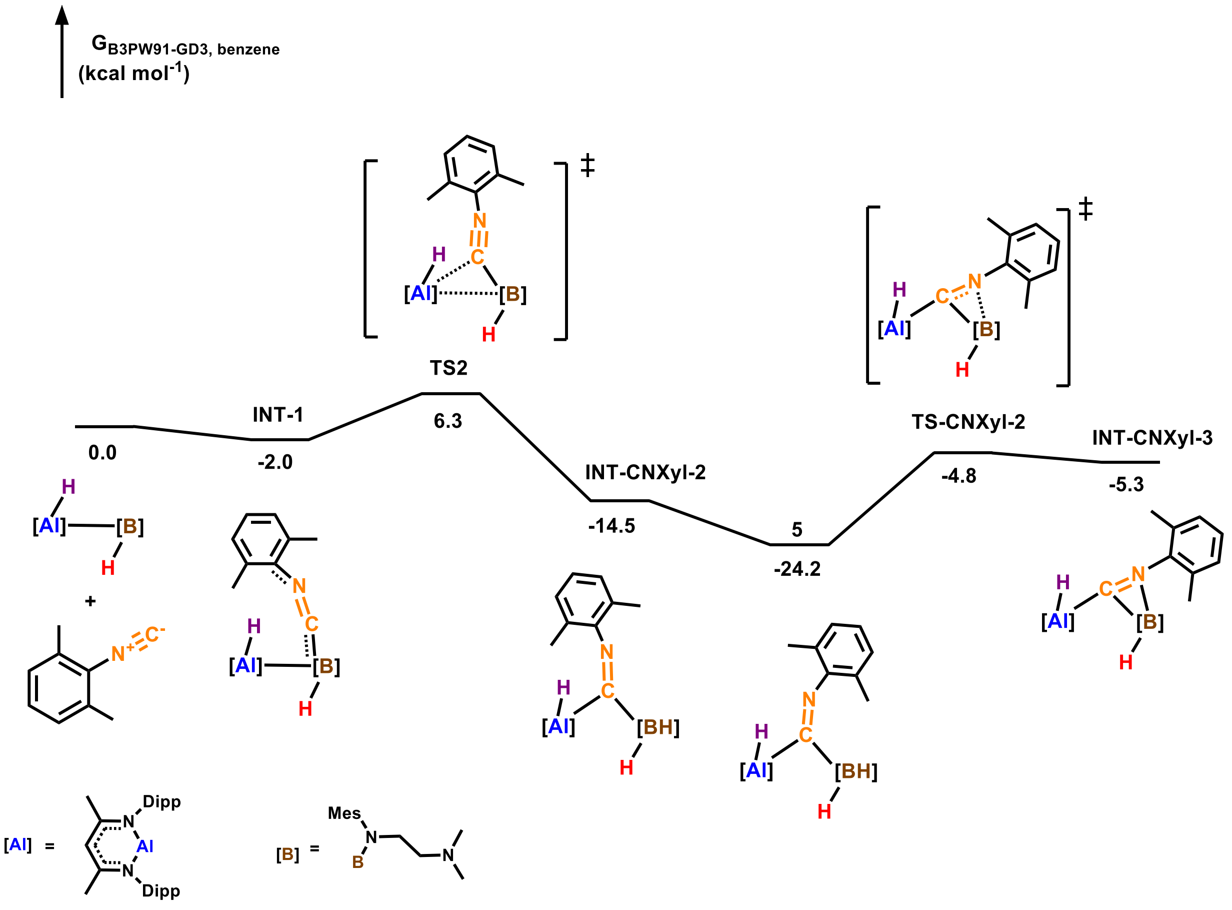

Figure S. Proposed pathway for the insertion of CNXyl to Al-B bond based on DFT calculations G09: B3PW91-D3 / 6-311+G**/ PCM (benzene) // B3PW91-D3 / 6-31G** / 6-311+G* / SDDAll (Al). Free Energy kcal mol^{-1}.

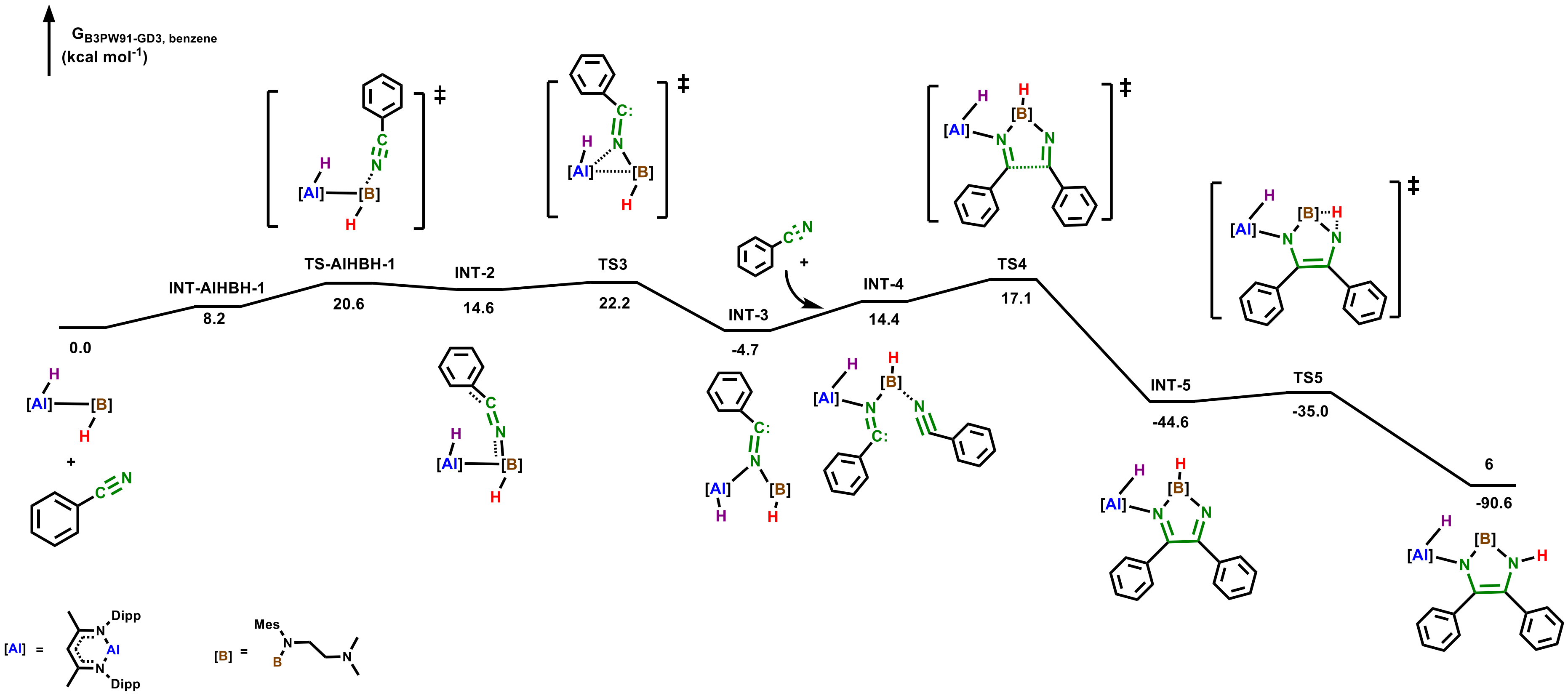

Figure S. Proposed pathway for the coupling of PhCN and hydrogen transfer based on DFT calculations G09: B3PW91-D3 / 6-311+G**/ PCM (benzene) // B3PW91-D3 / 6-31G** / 6-311+G* / SDDAll (Al). Free Energy kcal mol^{-1}.

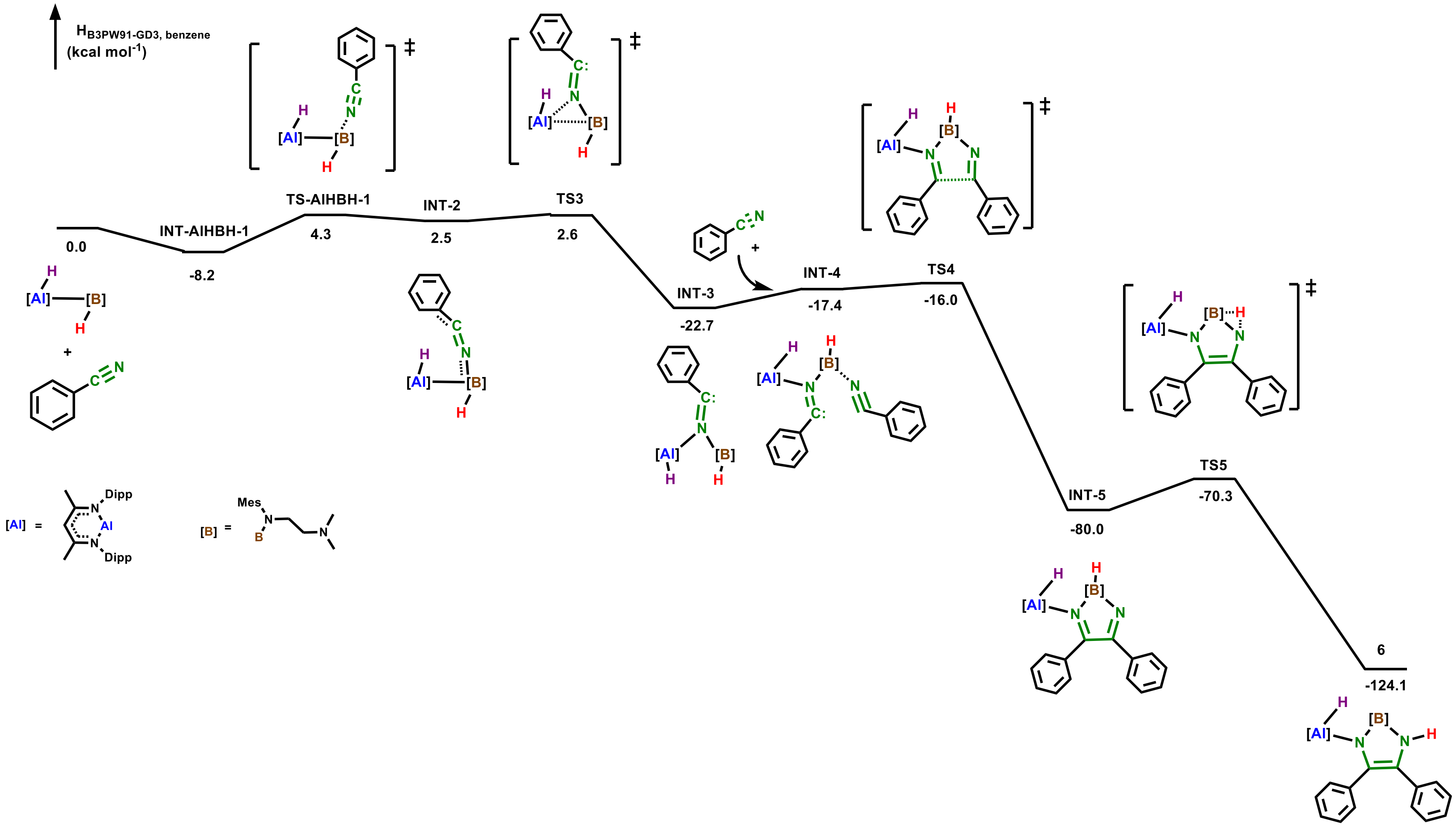

Figure S. Proposed pathway for the coupling of PhCN and hydrogen transfer based on DFT calculations G09: B3PW91-D3 / 6-311+G**/ PCM (benzene) // B3PW91-D3 / 6-31G** / 6-311+G* / SDDAll (Al). Enthalpy kcal mol^{-1}.

4.3. Optimised structures

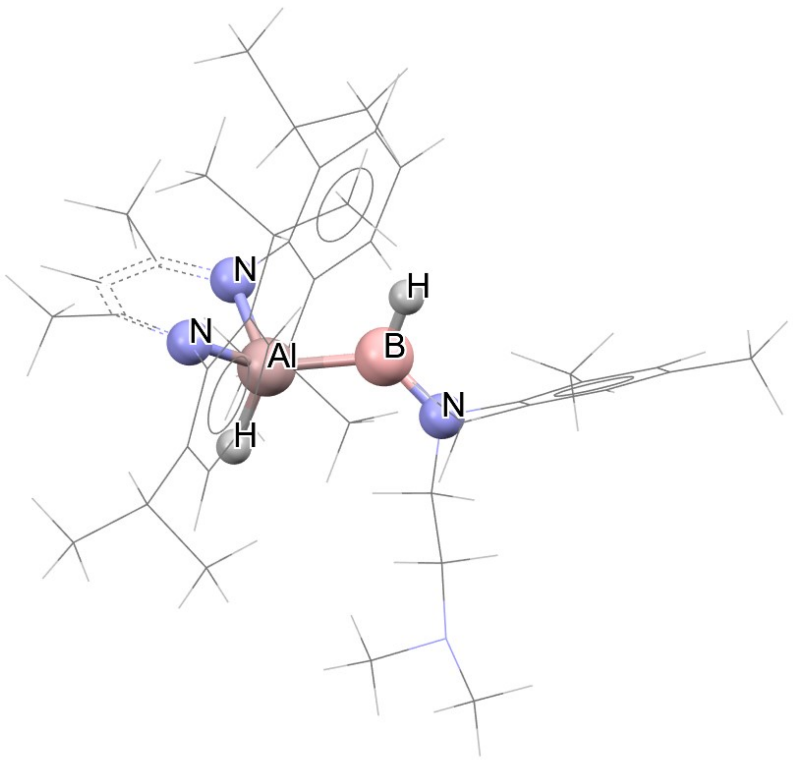

3a

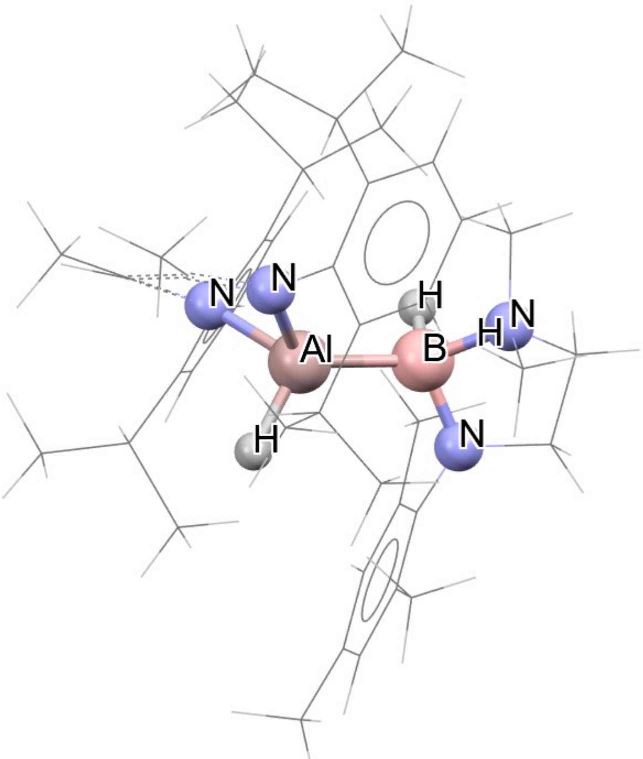

3a'

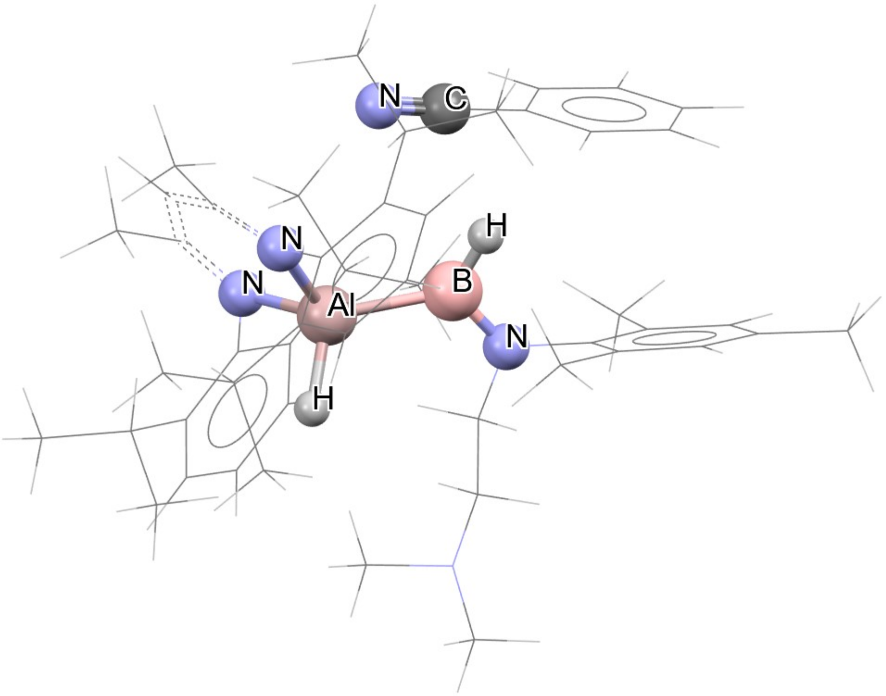

Int-AlHBH-1

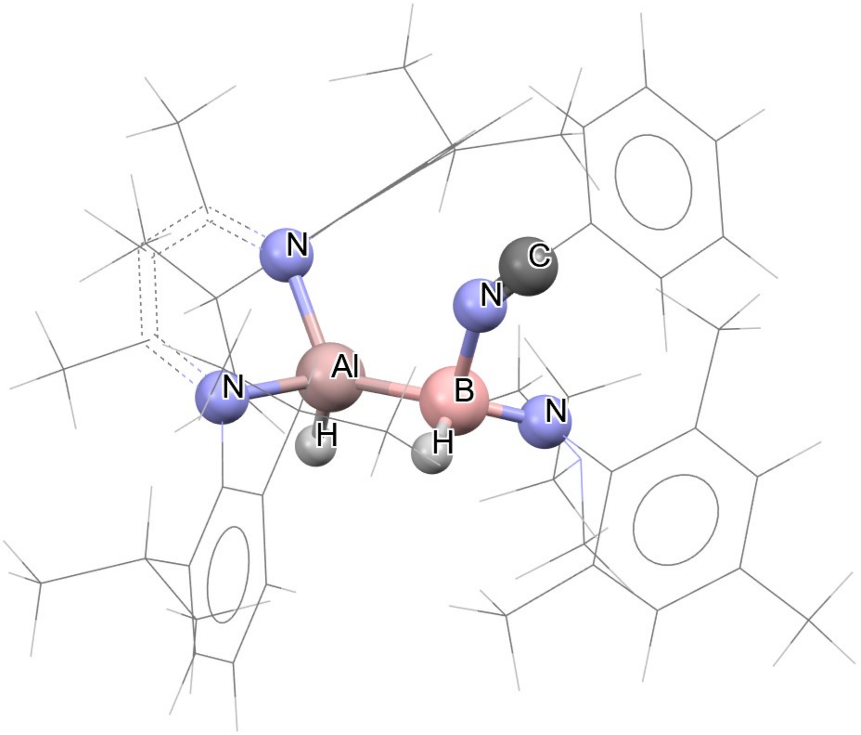

TS-AlHBH-1

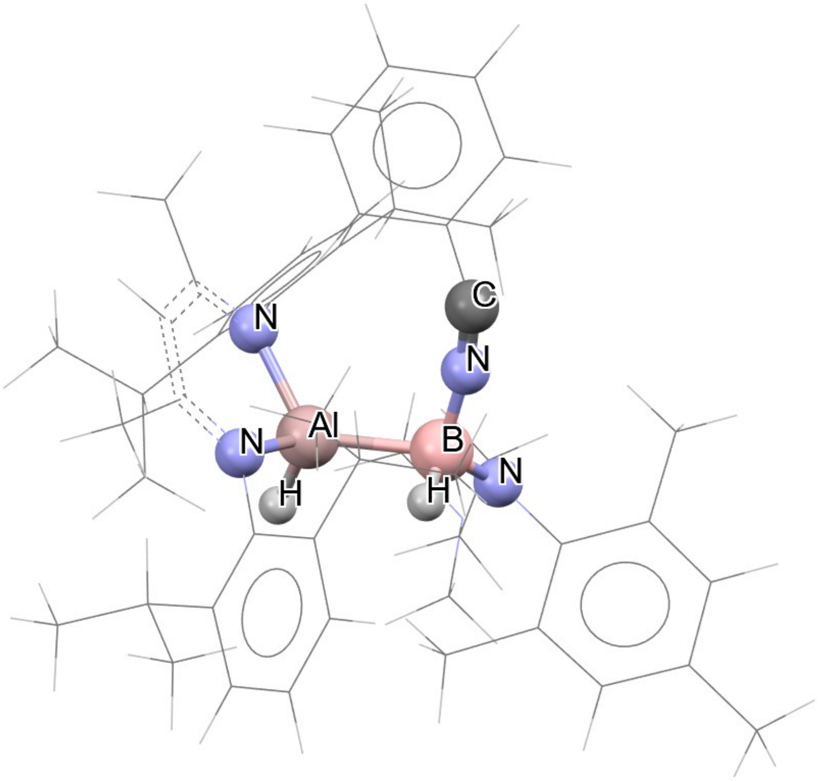

Int-2

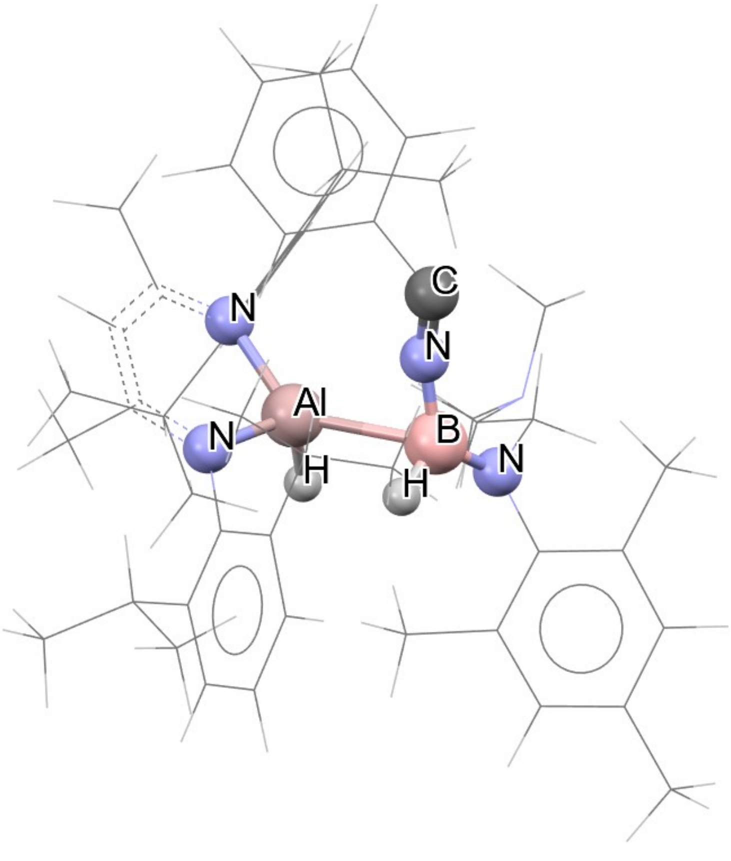

TS3

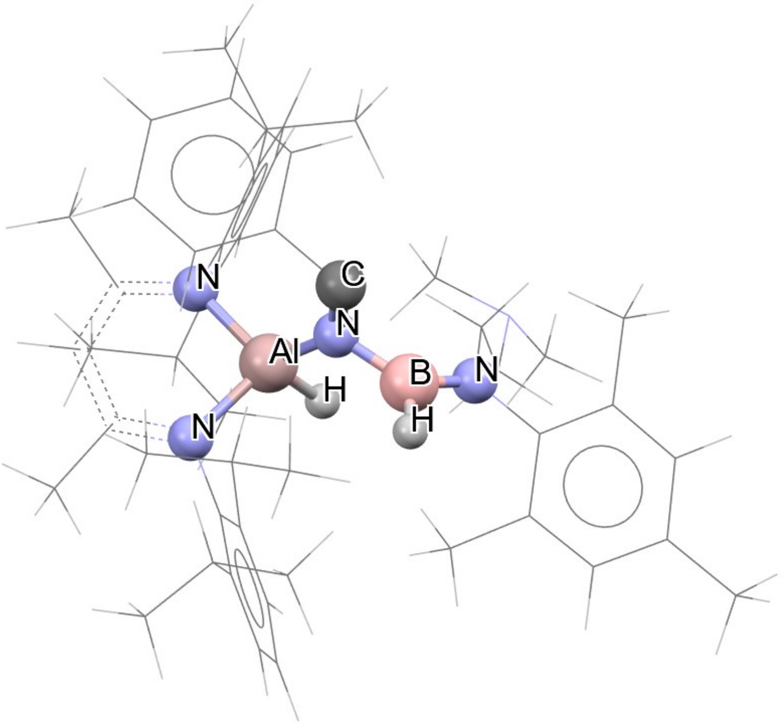

Int-3

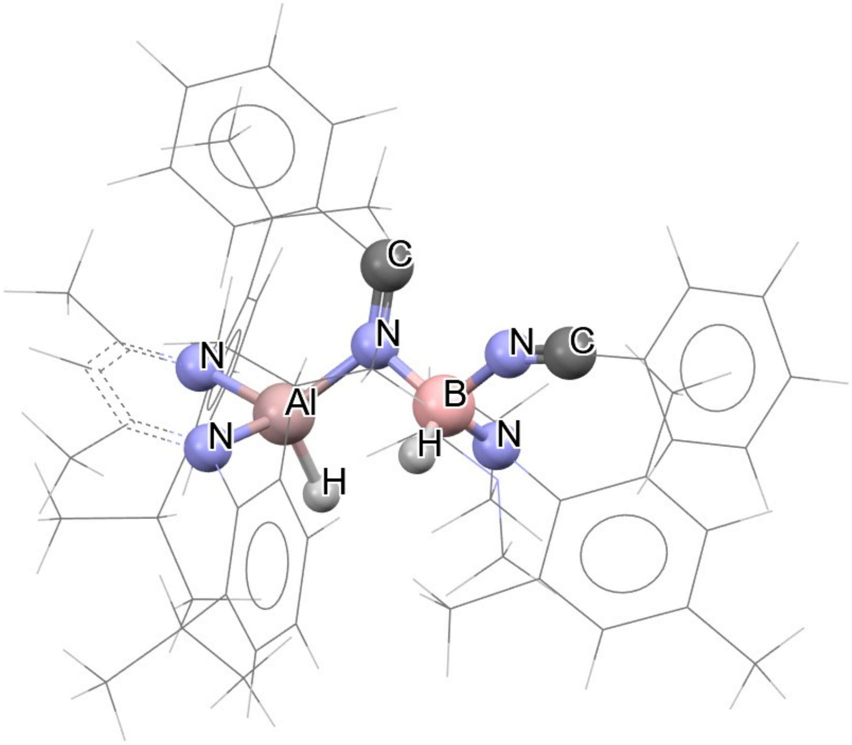

Int-4

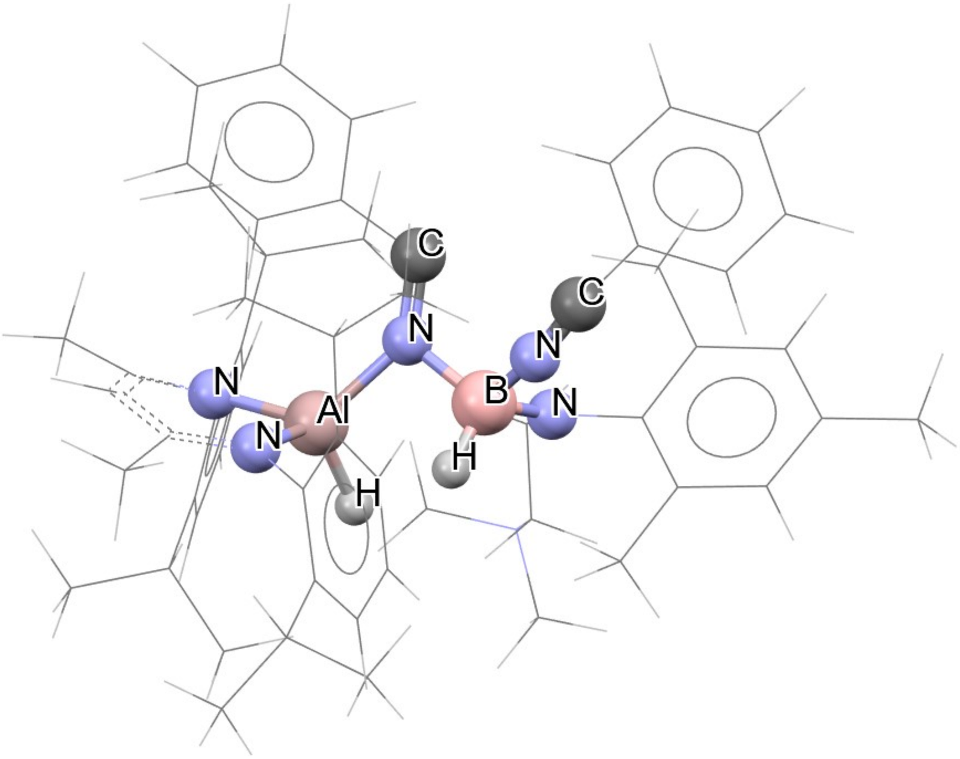

TS4

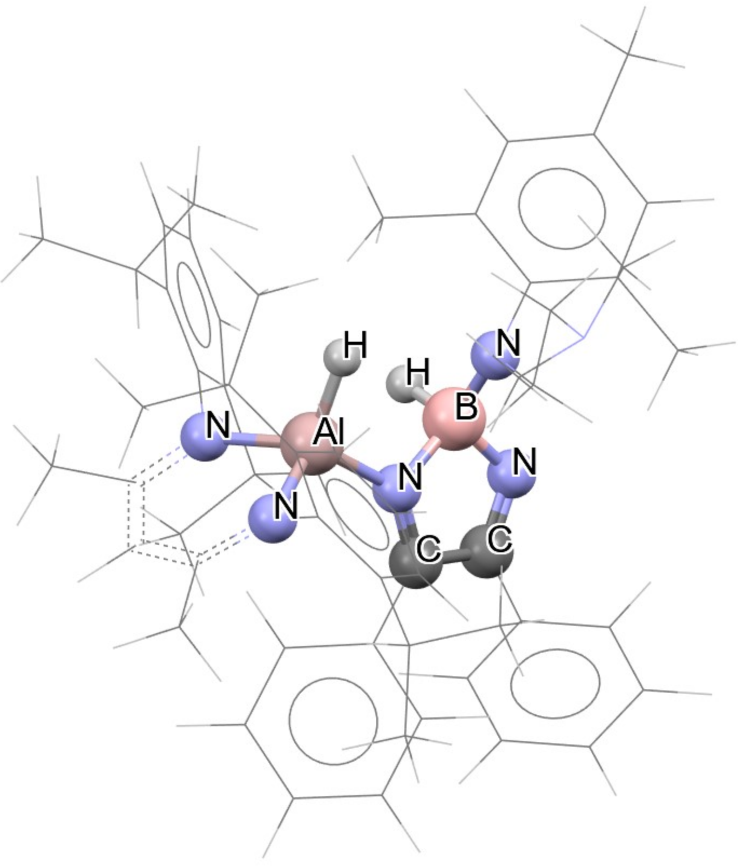

Int-5

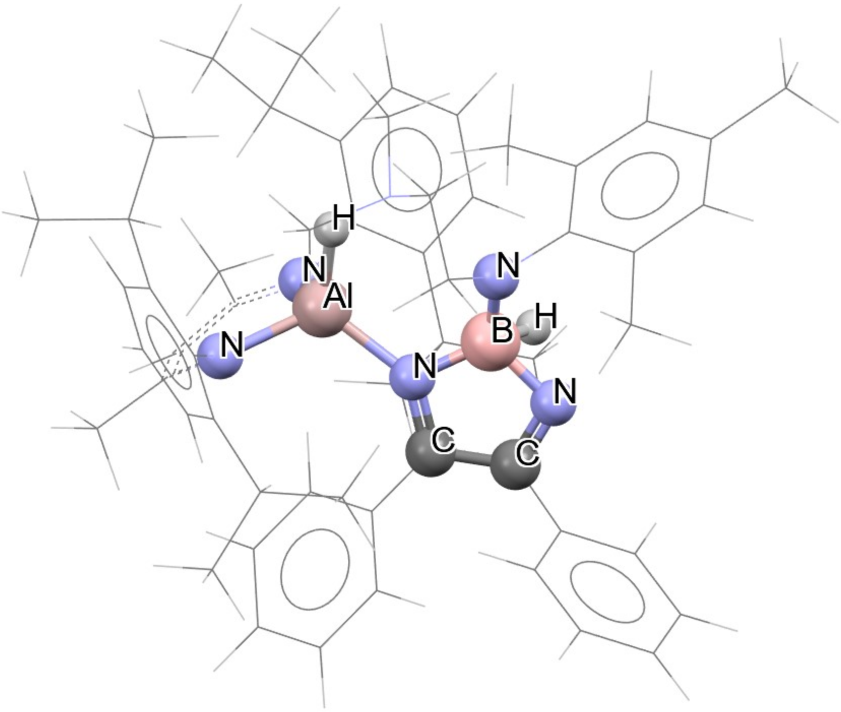

TS5

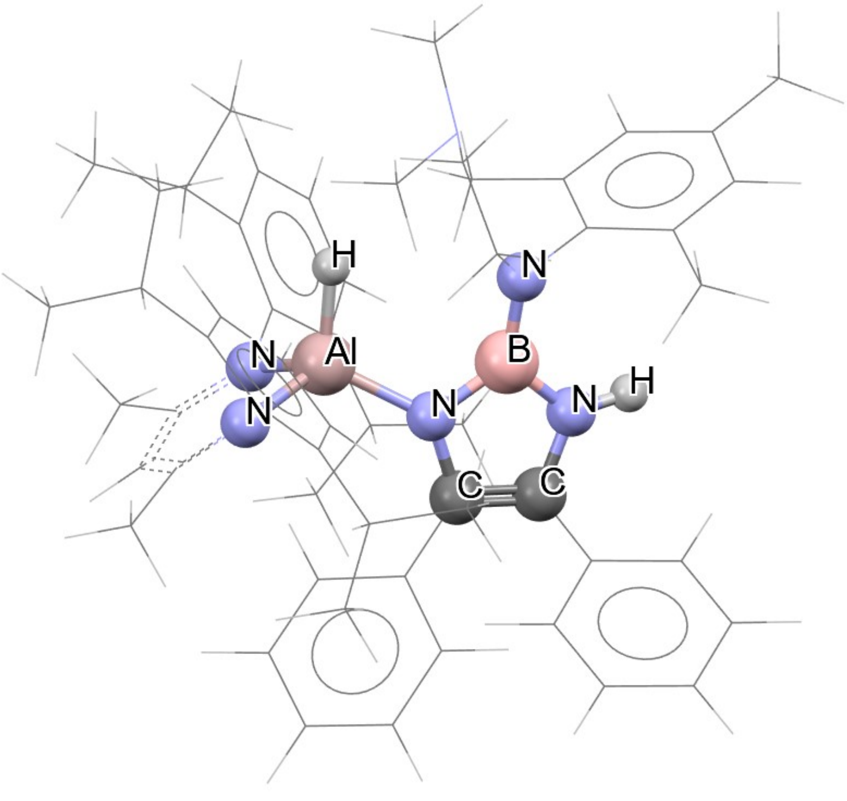

6

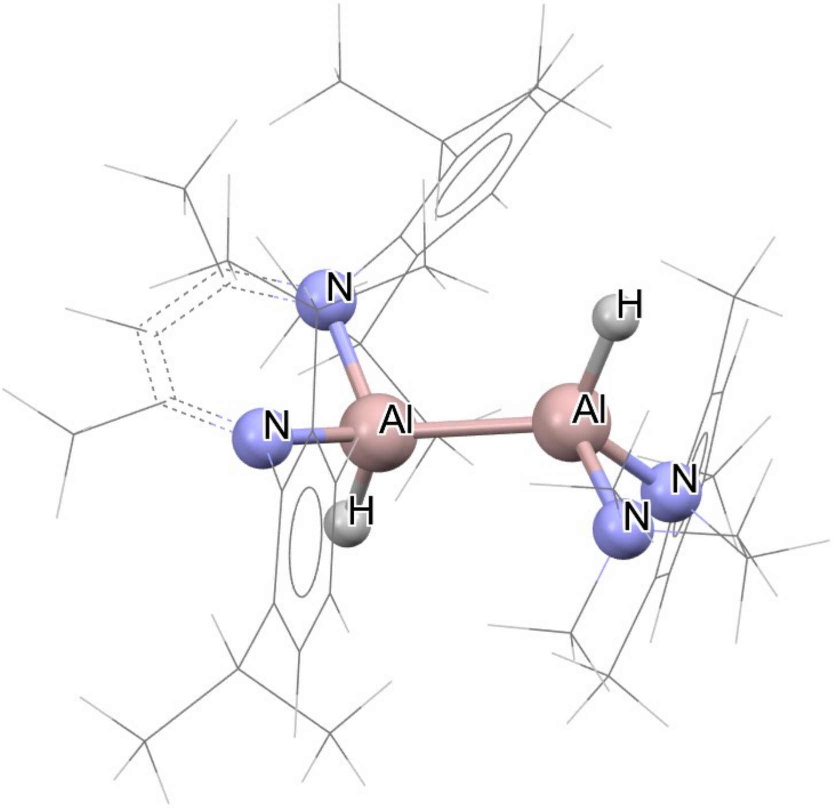

3b

4.4 Other Possible Mechanism Pathway

In addition to the reaction pathways shown in Figure S4, Figure S6 considered the reaction pathways resulting from C=N π bond. Figure S7 considered the reaction pathways resulting from hydrogen transfer from Al-B.[a]

4.4.1: Possible Mechanism Pathway One：

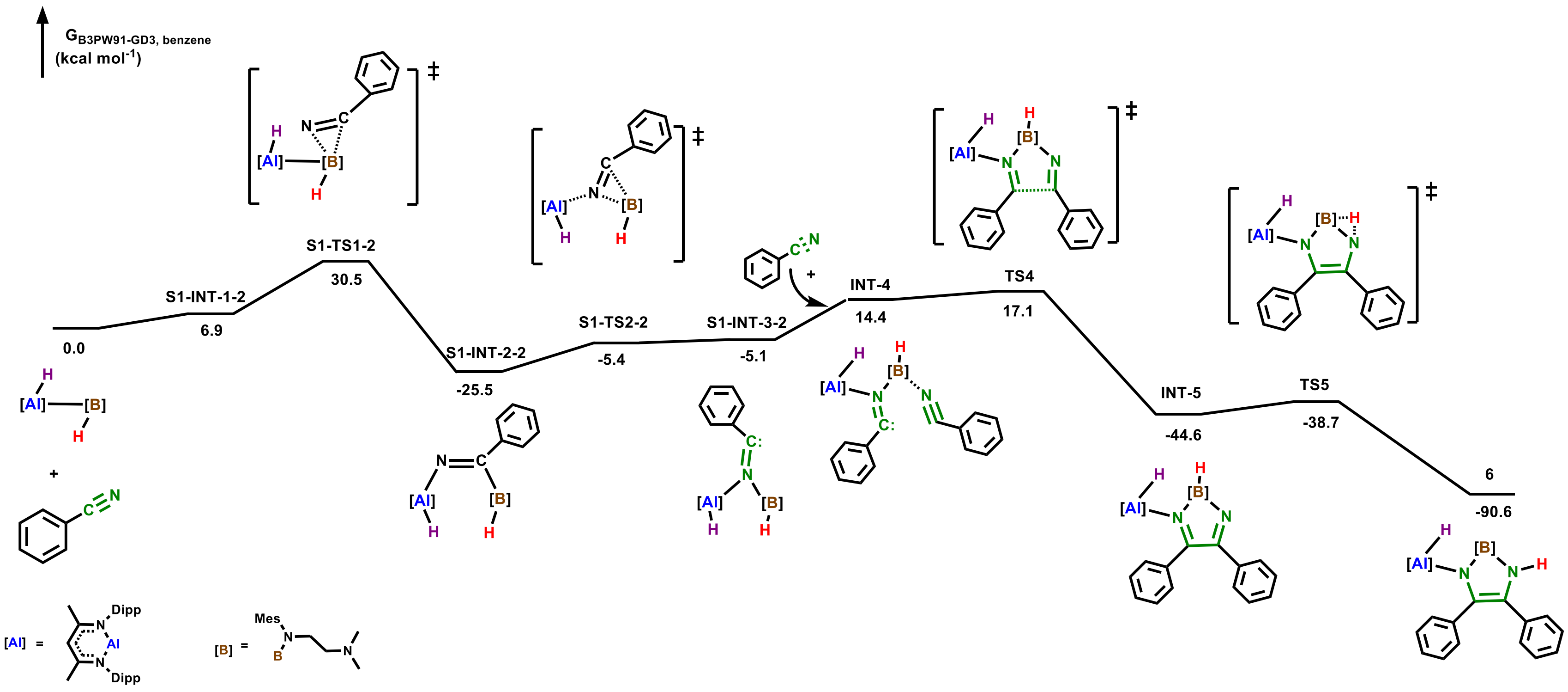

Figure S. Proposed pathway for the coupling of coupling of PhCN and hydrogen transfer based on DFT calculations G09: B3PW91-D3 / 6-311+G**/ PCM (benzene) // B3PW91-D3 / 6-31G** / 6-311+G* / SDDAll (Al)

4.4.2: Possible Mechanism Pathway Two：

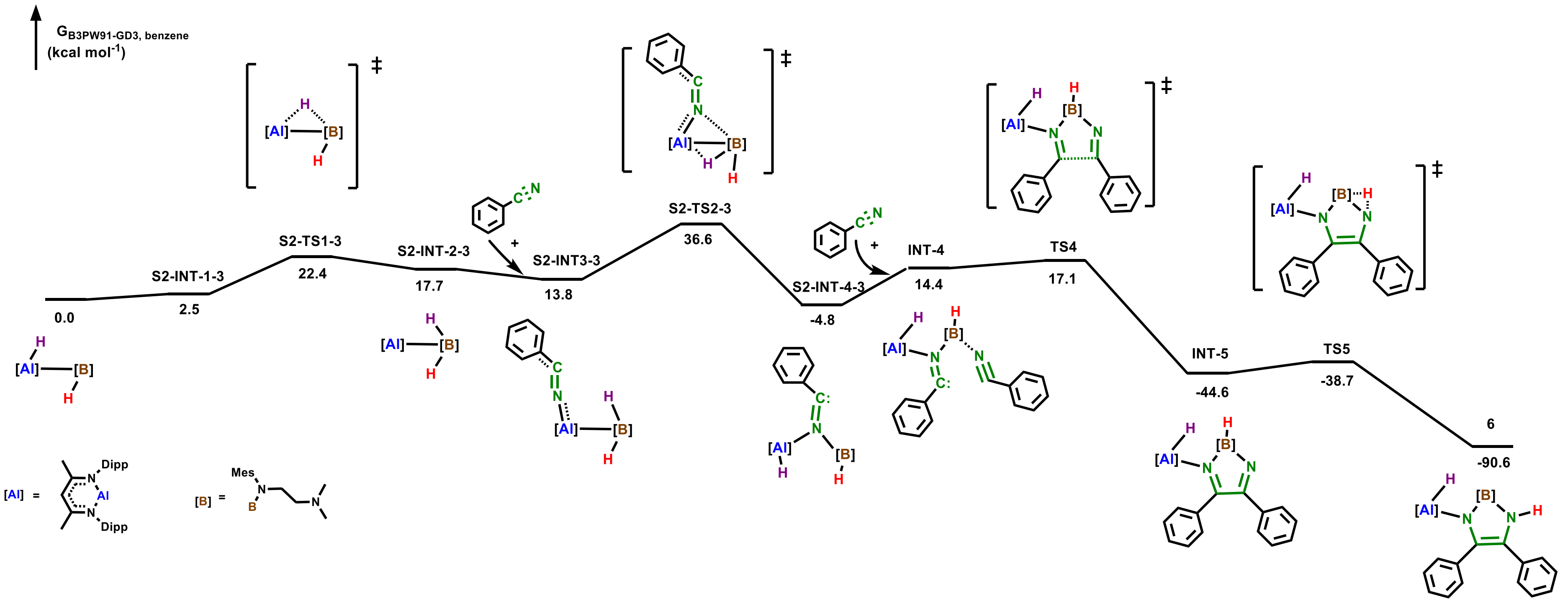

Figure S. Proposed pathway for the coupling of coupling of PhCN and hydrogen transfer based on DFT calculations G09: B3PW91-D3 / 6-311+G**/ PCM (benzene) // B3PW91-D3 / 6-31G** / 6-311+G* / SDDAll (Al)

[a] Different possible reaction mechanism from INT-1-2 to INT-4-3 were reported in Figure S7 and Figure S

4.5. NBO Analysis

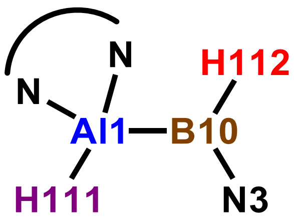

3a

Atom	No	Charge
Al1	1	1.37
B10	10	0.03
H111	111	-0.43
H112	112	-0.09

Table S. NPA change data for **3a**

Wiberg bond index matrix	
Al1 - B10	0.822
Al1 - H111	0.748
B10 - H112	0.951
B10 - N3	1.119

Table S. WBI data for **3a**

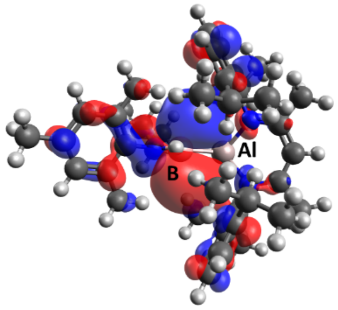

LUMO+1 (-0.386 eV)

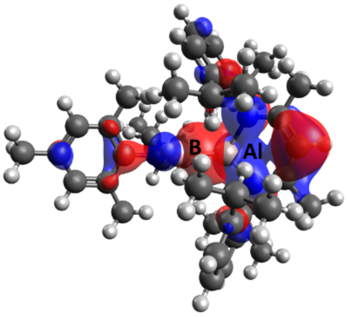

HOMO (-5.465 eV)

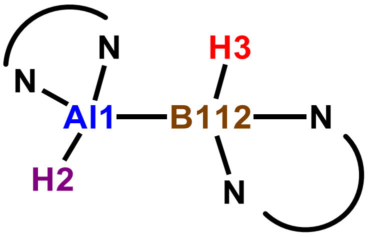

3a’

Atom	No	Charge
Al1	1	1.60
B112	10	-0.17
H2	111	-0.52
H3	112	-0.09

Table S. NPA change data for **3a’**

Wiberg bond index matrix	
Al1 - B112	0.712
Al1 - H2	0.638
B112 - H3	0.926

Table S. WBI data for **3a’**

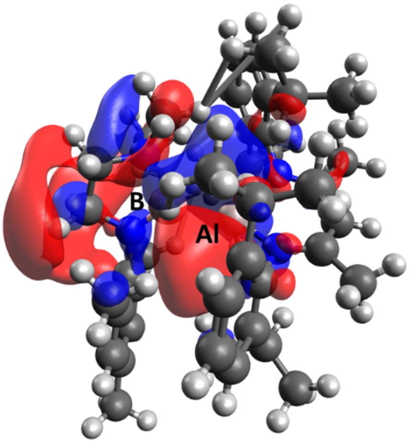

LUMO+8 (1.150 eV)

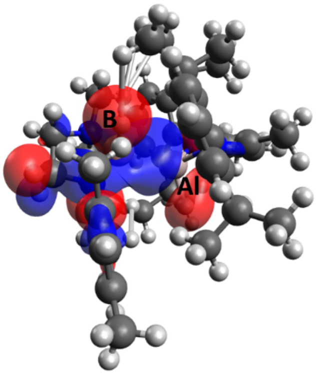

HOMO (-4.227 eV)

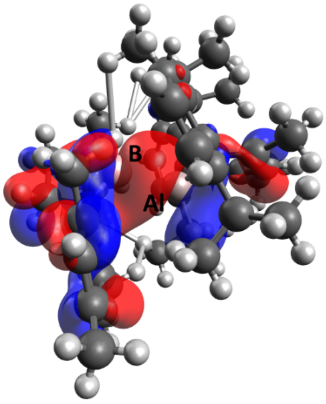

HOMO-4 (-5.938 eV)

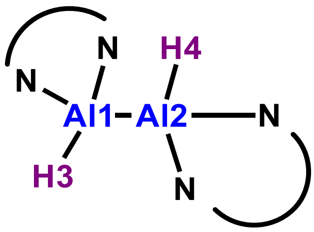

3b

Atom	No	Charge
Al1	1	1.05
Al2	2	1.10
H3	3	-0.45
H4	4	-0.49

Table S. NPA change data for **3b**

Wiberg bond index matrix	
Al1 - Al2	0.917
Al1 - H3	0.723
Al2 - H4	0.673

Table S. WBI data for **3b**

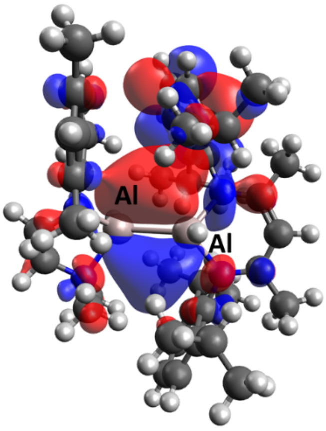

LUMO+3 (-0.002 eV)

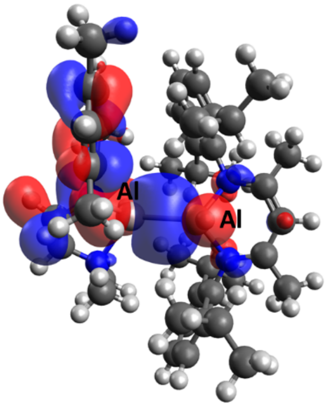

HOMO (-4.653 eV)

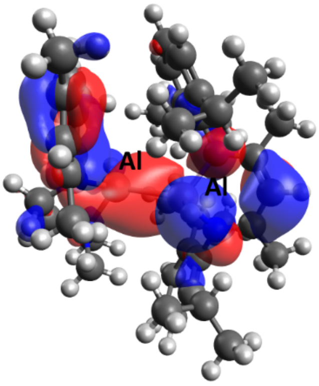

HOMO-1 (-5.333 eV)

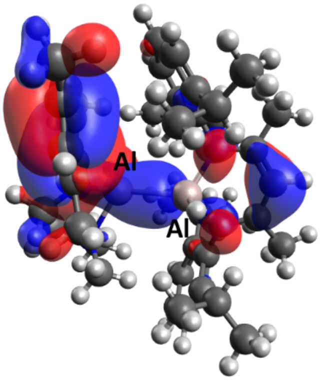

HOMO-2 (-5.722 eV)

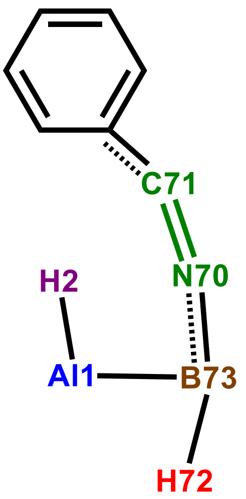

Int-2

Atom	No	Charge
Al1	1	1.68
B73	73	-0.22
H2	2	-0.51
H72	72	0.02
N70	70	-0.33
C71	71	0.39

Table S. NPA change data for **Int-2**

Wiberg bond index matrix	
Al1 - B73	0.513
Al1 - H2	0.634
B73 - N70	0.874
B73 - H72	0.875
N70 - C71	2.385

Table S. WBI data for **Int-2**

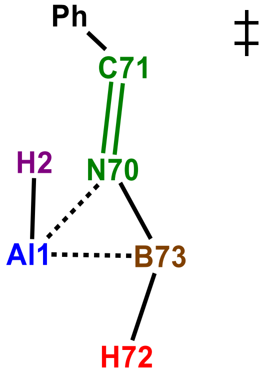

TS3

Atom	No	Charge
Al1	1	1.74
B73	73	0.33
H2	2	-0.54
H72	72	0.00
N70	70	-0.65
C71	71	0.20

Table S. NPA change data for **TS3**

Wiberg bond index matrix	
Al1 - B73	0.253
Al1 - H2	0.563
Al1 - N70	0.122
B73 - N70	1.066
B73 - H72	0.010
N70 - C71	1.888

Table S. WBI data for **TS3**

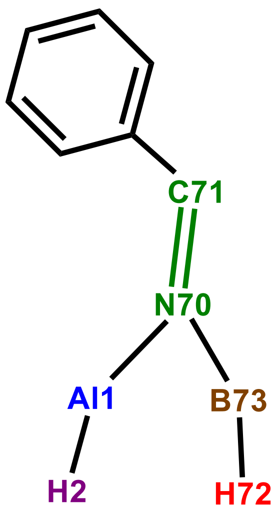

Int-3

Atom	No	Charge
Al1	1	1.94
B73	73	0.81
H2	2	-0.58
H72	72	-0.08
N70	70	-1.17
C71	71	0.13

Table S. NPA change data for **Int-3**

Wiberg bond index matrix	
Al1 - B73	0.030
Al1 - H2	0.560
Al1 - N70	0.220
B73 - N70	0.738
B73 - H72	0.936
N70 - C71	1.552

Table S. WBI data for **Int-3**

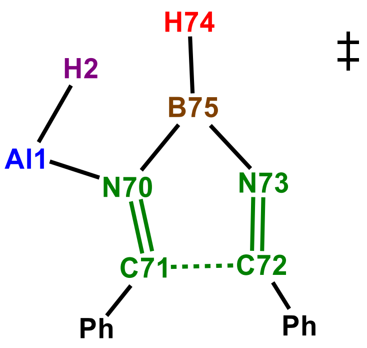

TS4

Atom	No	Charge
Al1	1	1.85
B75	75	0.63
H2	2	-0.54
H74	74	-0.07
N70	70	-1.09
C71	71	0.08
N73	73	-0.33
C72	72	0.57

Table S. NPA change data for **TS4**

Wiberg bond index matrix	
Al1 - H2	0.600
Al1 - N70	0.254
B75 - N70	0.730
B75 - N73	0.673
B75 - H74	0.877
N70 - C71	1.572
N73 - C72	2.574
C71 - C72	0.014

Table S. WBI data for **TS4**

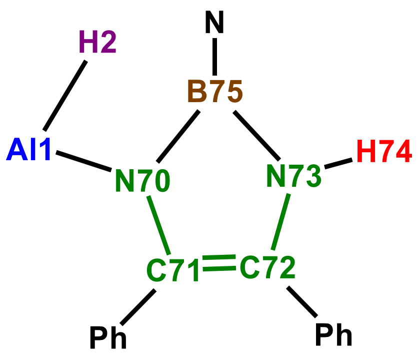

6

Atom	No	Charge
Al1	1	1.99
B75	75	1.04
H2	2	-0.58
H74	74	0.42
N70	70	-1.20
C71	71	0.11
N73	73	-0.83
C72	72	0.12

Table S. NPA change data for **6**

Wiberg bond index matrix	
Al1 - H2	0.568
Al1 - N70	0.251
B75 - N70	0.968
B75 - N73	0.944
N73 - H74	0.797
N70 - C71	1.100
N73 - C72	1.107
C71 - C72	1.520

Table S. WBI data for **6**

4.6. QTAIM Analysis

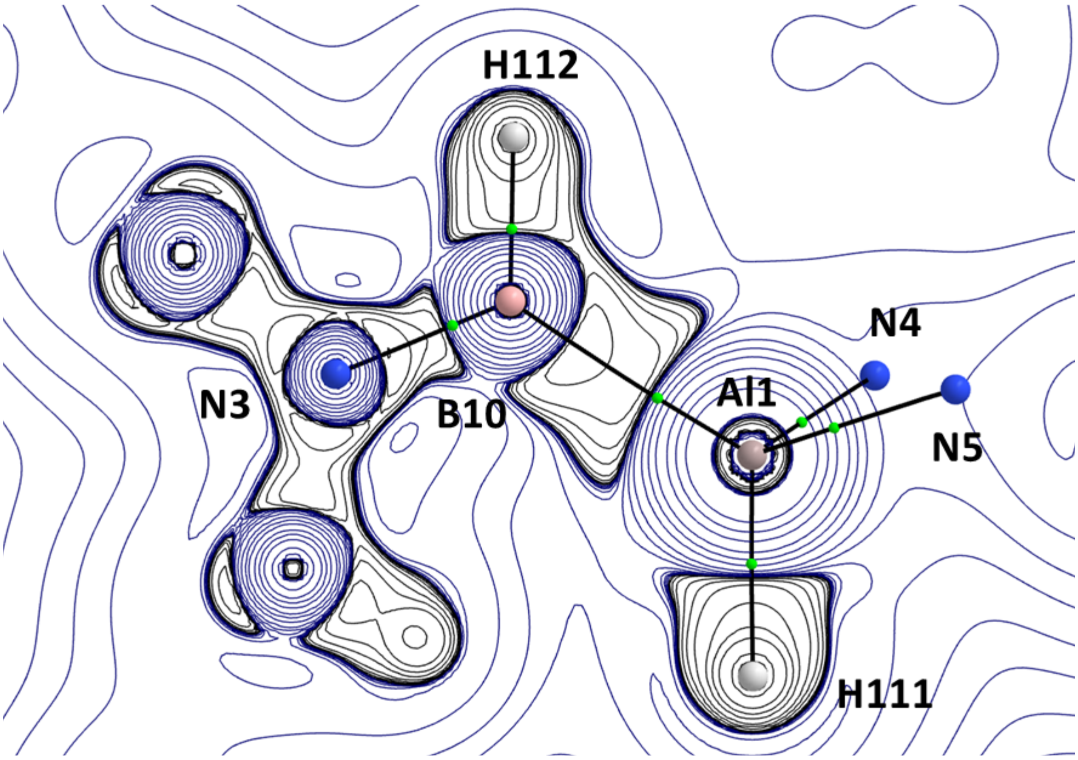

Figure S17. Plot of the Laplacian of the electron density $\nabla^2 \rho$ (r) for **3a**

Atoms	ρ (r) (e bohr^{-3})	∇^2 ρ (r) (e bohr^{-5})	Ellipticity
Al1 - N4	0.067	0.383	0.086
Al1 - N5	0.066	0.378	0.081
B10 - H112	0.171	-0.219	0.250
Al1 - H111	0.073	0.233	0.015
Al1 - B10	0.074	0.121	0.021
N3 - B10	0.200	0.645	0.084

Table S11. QTAIM data for **3a**

Atom	No	Charge
Al1	1	2.28
B10	10	0.65
H111	111	-0.75
H112	112	-0.62

Table S67. AIM change data for **3a**

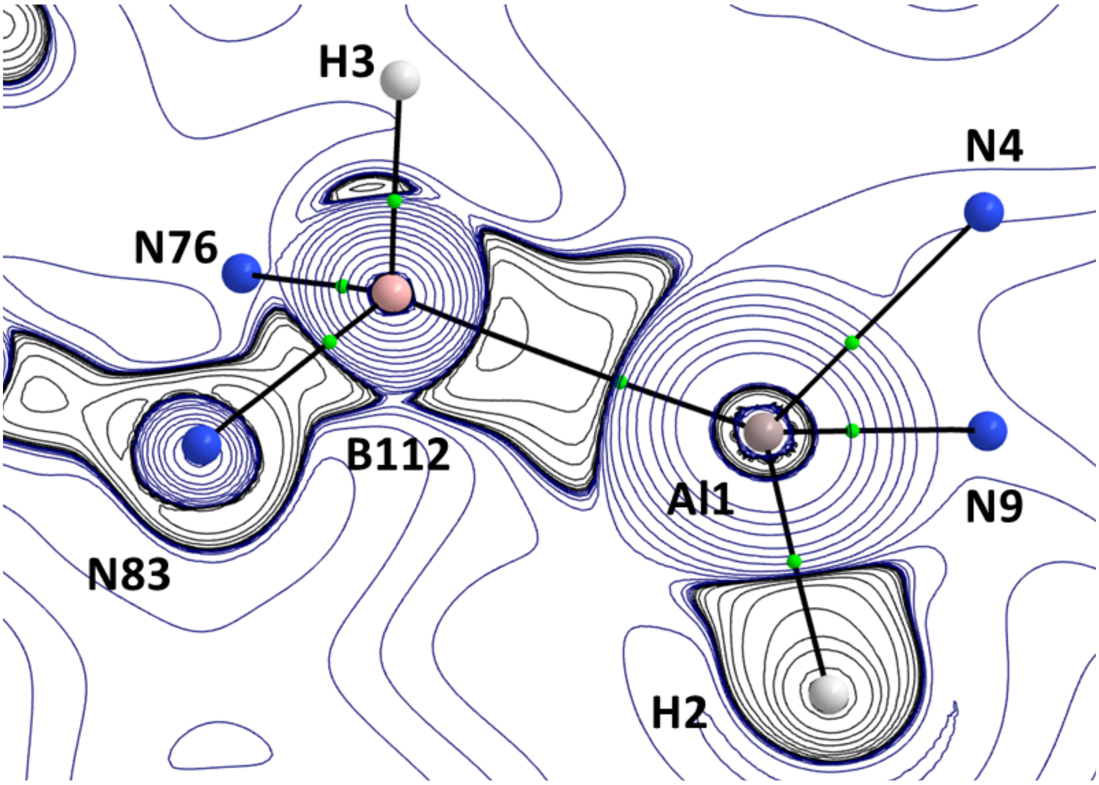

Figure S17. Plot of the Laplacian of the electron density $\nabla^2 \rho$ (r) for **3a'**

Atoms	ρ (r) (e bohr^{-3})	∇^2 ρ (r) (e bohr^{-5})	Ellipticity
Al1 - B112	0.071	0.065	0.029
Al1 - H2	0.072	0.223	0.013
Al1 - N4	0.060	0.322	0.080
Al1 - N9	0.059	0.311	0.089
H3 - B112	0.160	-0.165	0.160
N76 - B112	0.113	0.324	0.128
N83 - B112	0.155	0.322	0.211

Table S11. QTAIM data for **3a'**

Atom	No	Charge
Al1	1	2.21
H2	2	-0.75
H3	3	-0.63
B112	112	0.77

Table S67. AIM change data for **3a'**

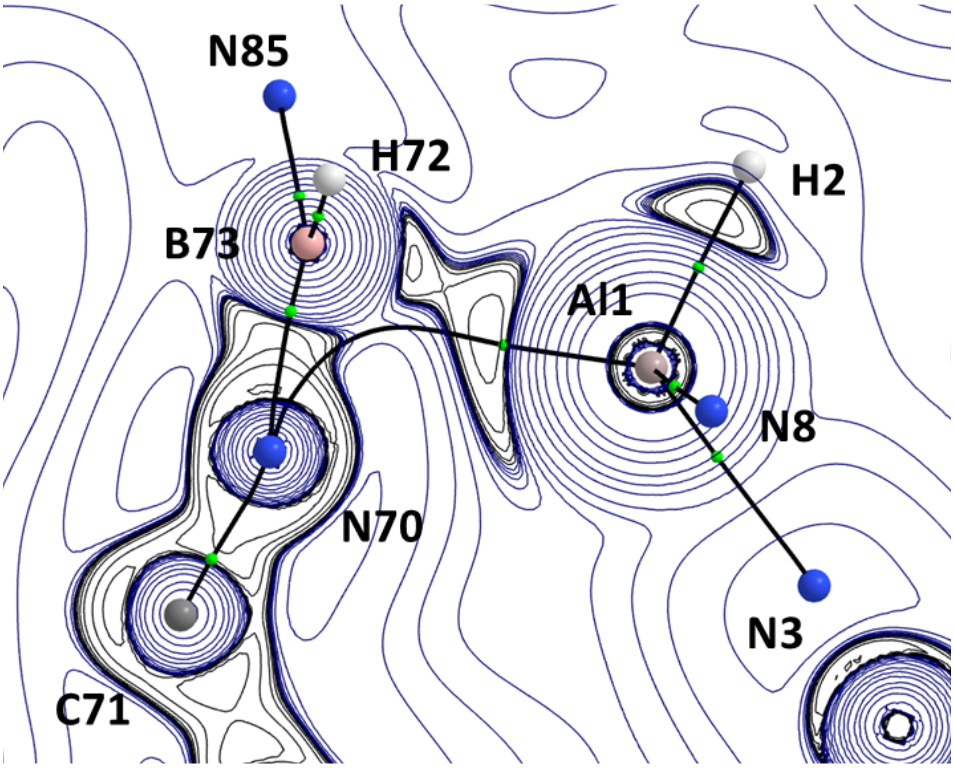

Figure S17. Plot of the Laplacian of the electron density $\nabla^2 \rho$ (r) for **TS3**

Atoms	ρ (r) (e bohr^{-3})	∇^2 ρ (r) (e bohr^{-5})	Ellipticity
N70 - C71	0.372	-0.067	0.192
N70 - B73	0.186	0.707	0.023
H72 - B73	0.172	-0.217	0.039
B73 - N85	0.188	0.407	0.119
Al1 - N8	0.069	0.383	0.105
Al1 - N70	0.040	-0.015	0.555
Al1 - N3	0.070	0.394	0.108
Al1 - H2	0.080	0.259	0.0297

Table S11. QTAIM data for **TS3**

Atom	No	Charge
1	Al1	2.18
2	H2	-0.70
70	N70	-1.75
71	C71	0.58
72	H72	-0.58
73	B73	1.82

Table S67. AIM change data for **TS3**

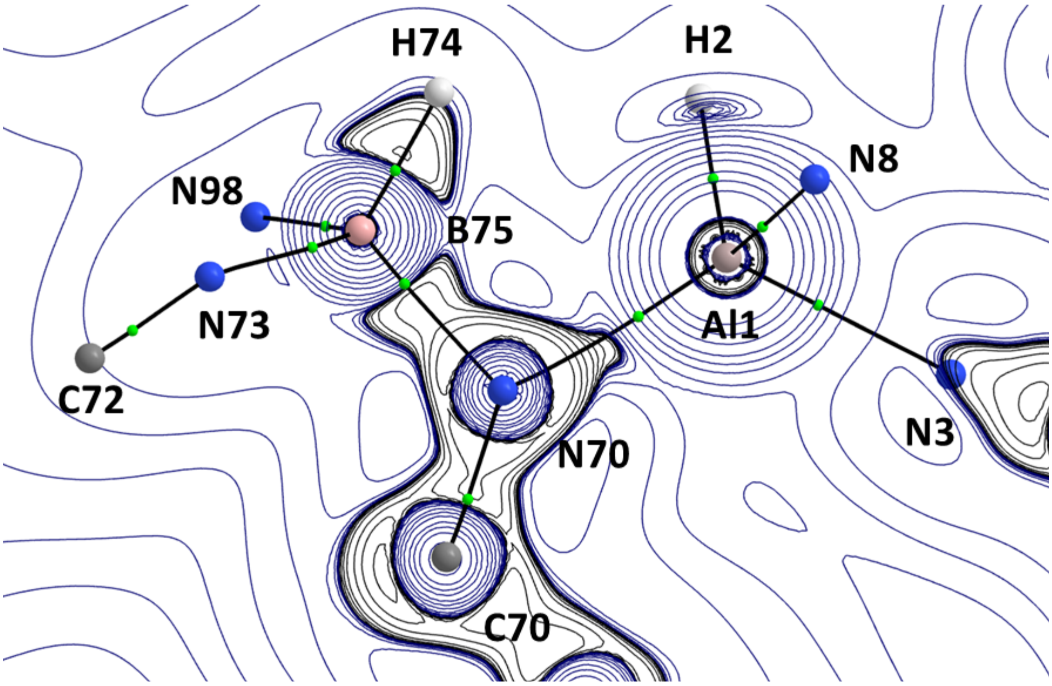

Figure S17. Plot of the Laplacian of the electron density $\nabla^2 \rho$ (r) for **TS4**

Atoms	ρ (r) (e bohr^{-3})	∇^2 ρ (r) (e bohr^{-5})	Ellipticity
Al1 - H2	0.081	0.267	0.013
Al1 - N3	0.071	0.402	0.085
Al1 - N70	0.075	0.396	0.053
Al1 - N8	0.067	0.375	0.091
B75 - N98	0.166	0.267	0.221
C72 - N73	0.467	-0.102	0.039
H74 - B75	0.167	-0.235	0.097
N70 - B75	0.155	0.175	0.162
N70 - C71	0.338	-0.651	0.170
N73 - B75	0.120	0.512	0.072

Table S11. QTAIM data for **TS4**

Atom	No	Charge
1	Al1	2.39
2	H2	-0.79
70	N70	-1.78
71	C71	0.39
72	C72	0.95
73	N73	-1.36
74	H74	-0.63
75	B75	2.08

Table S67. AIM change data for **TS4**

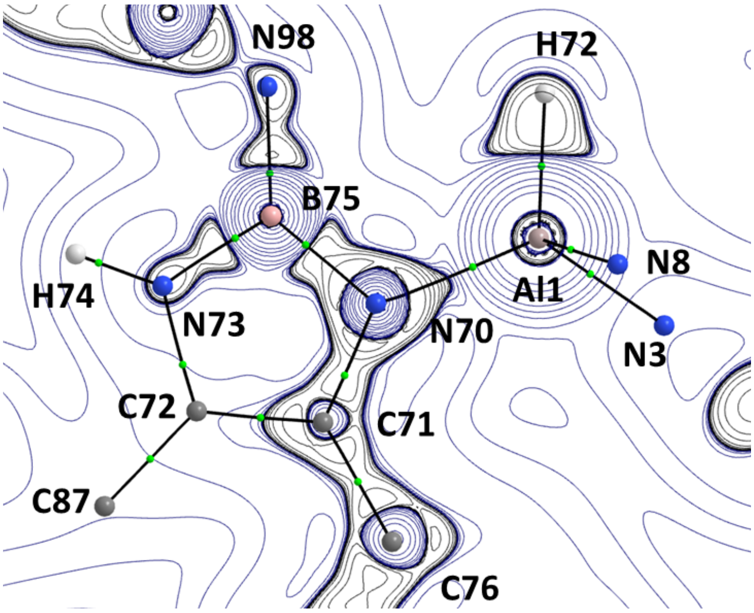

Figure S17. Plot of the Laplacian of the electron density $\nabla^2 \rho$ (r) for **6**

Atoms	ρ (r) (e bohr^{-3})	$\nabla^2 \rho$ (r) (e bohr^{-5})	Ellipticity
Al1 - H2	0.082	0.274	0.014
Al1 - N3	0.072	0.420	0.080
Al1 - N70	0.080	0.467	0.095
Al1 - N8	0.068	0.388	0.078
B75 - N98	0.194	0.376	0.106
C71 - C72	0.319	-0.876	0.395
C71 - C76	0.268	-0.677	0.099
C72 - C87	0.272	-0.693	0.115
C72 - N73	0.297	-0.790	0.137
N70 - B75	0.192	0.374	0.047
N70 - C71	0.284	-0.746	0.131
N73 - B75	0.191	0.495	0.046
N73 - H74	0.340	-1.657	0.040

Table S11. QTAIM data for **6**

Atom	No	Charge
1	Al1	2.41
2	H2	-0.73
70	N70	-1.78
71	C71	0.33
72	C72	0.36
73	N73	-1.46
74	H74	0.40
75	B75	2.14

Table S67. AIM change data for **6**

5) NMR Spectra

5.1. NMR Spectra of Isolated complexes

^{1}H NMR (C_6D_6, 400 MHz) of **3a**: ("*"is the solvent peak of **3a'**)

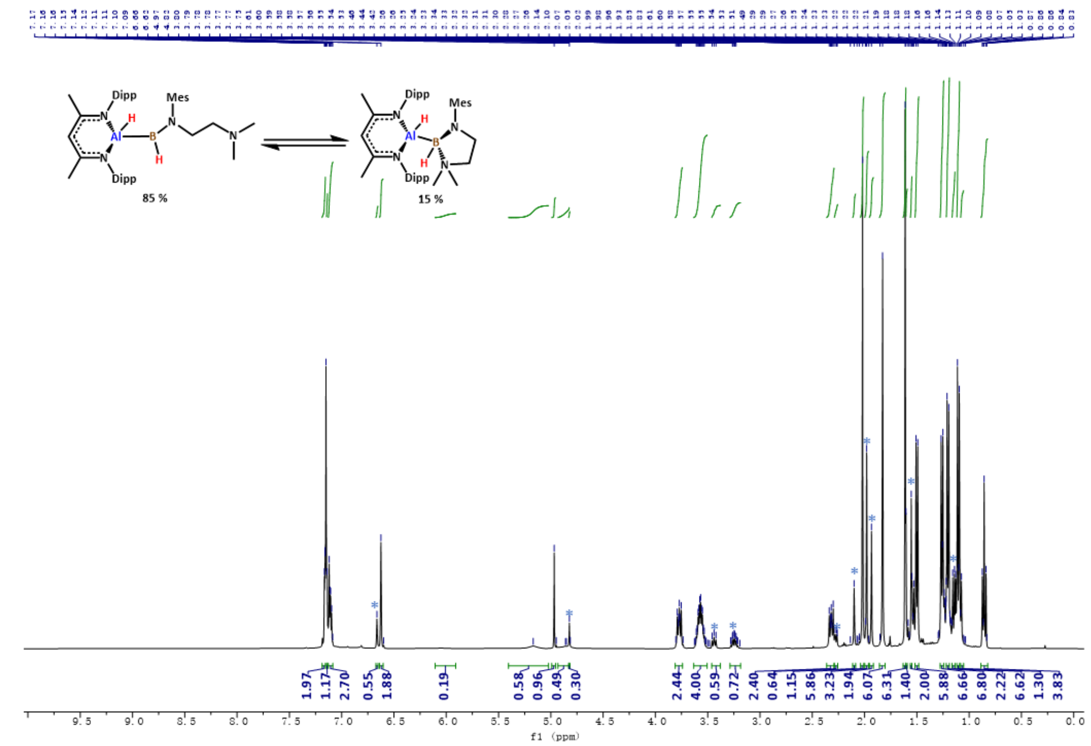

^{13}C NMR (C_6D_6, 101 MHz) of **3a**: ("*"is the solvent peak of **3a'**)

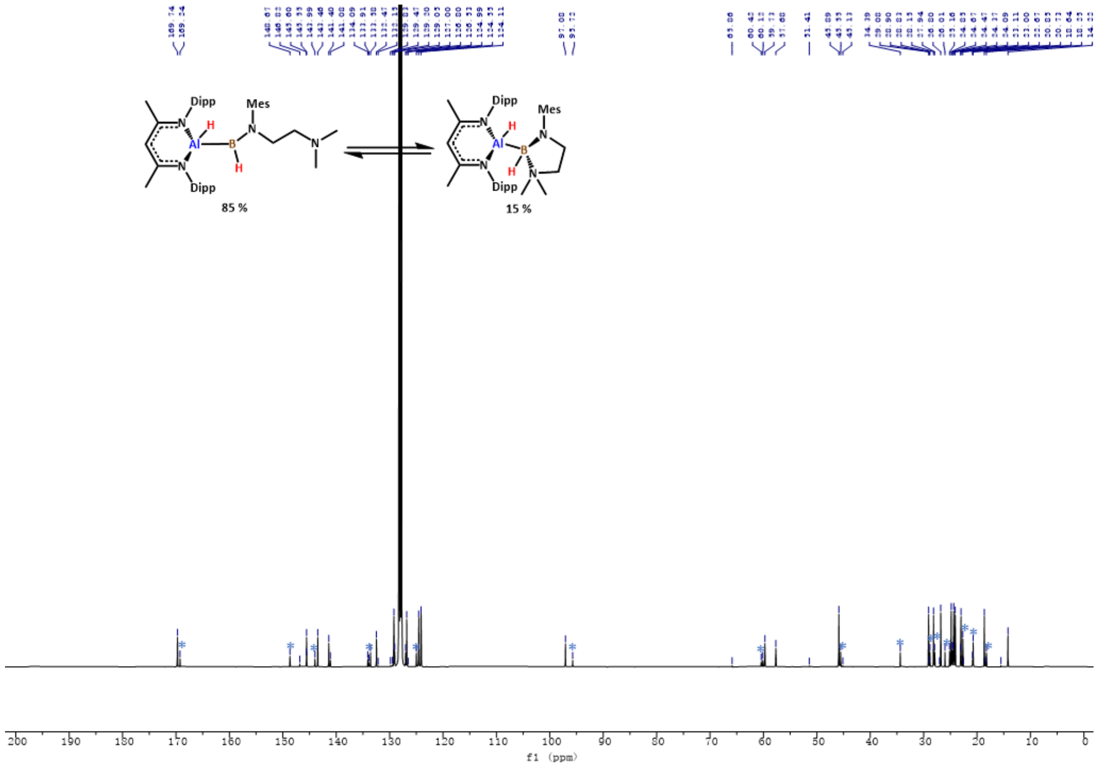

^{11}B NMR (C_6D_6, 128.0MHz) of **3a/3a'**:

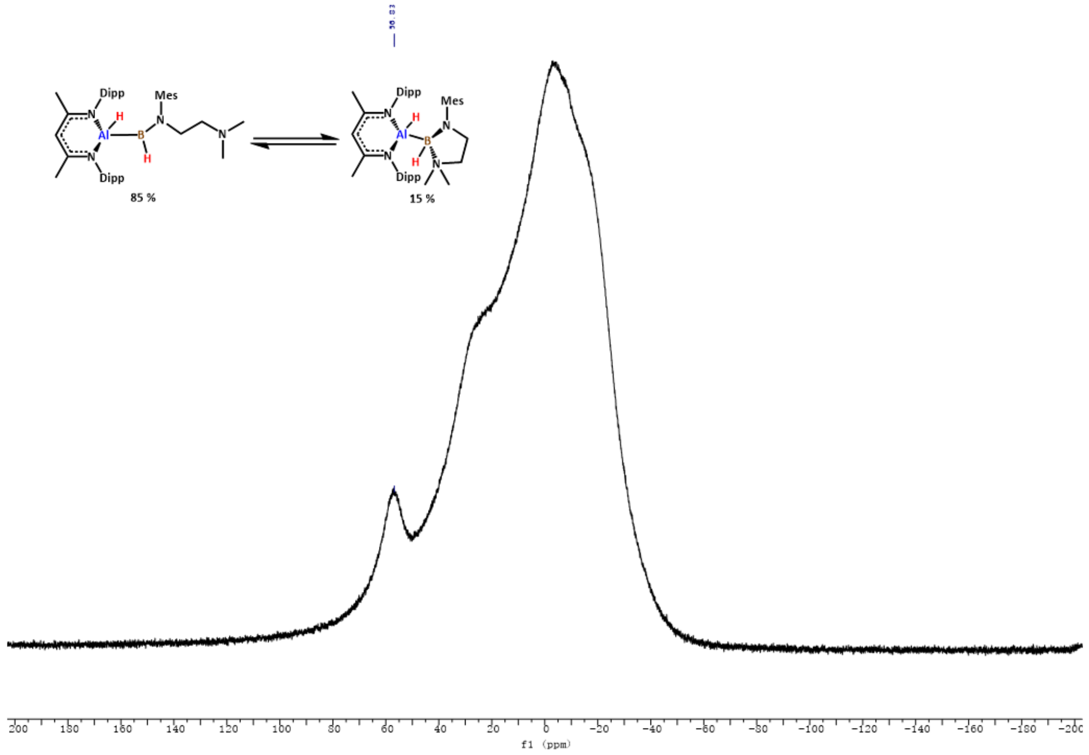

^{1}H NMR (C_6D_6, 400 MHz) of **3b**:

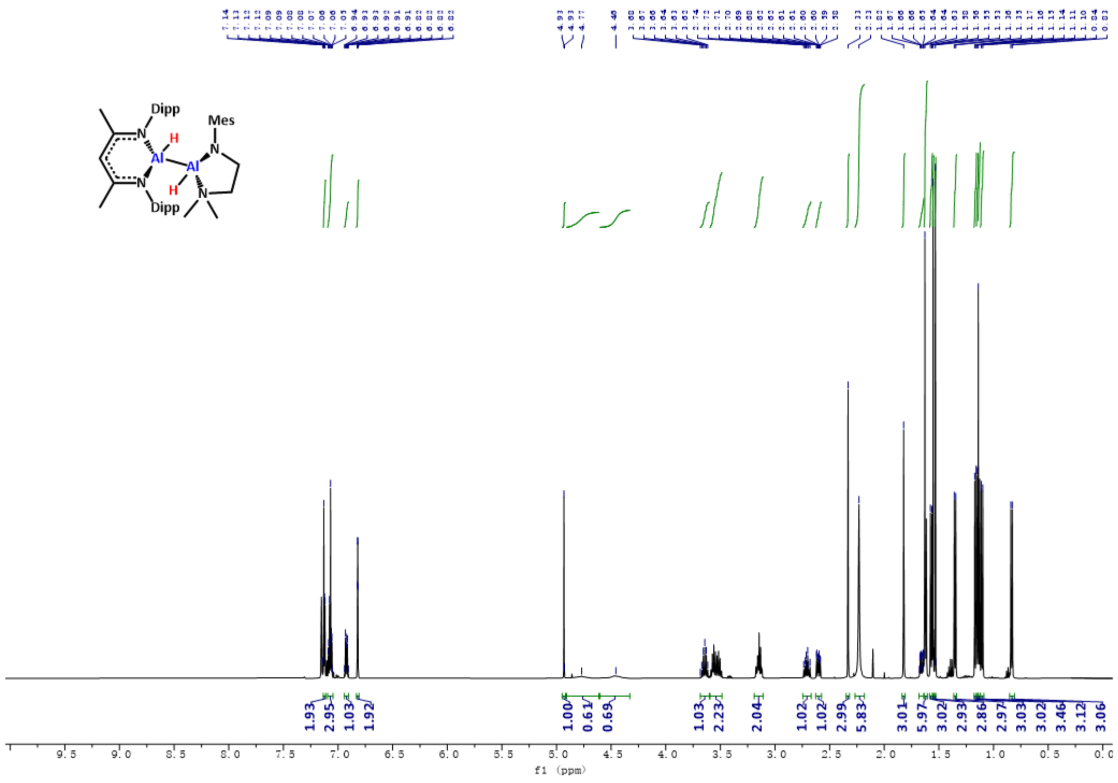

^{13}C NMR (C_6D_6, 101 MHz) of **3b**:

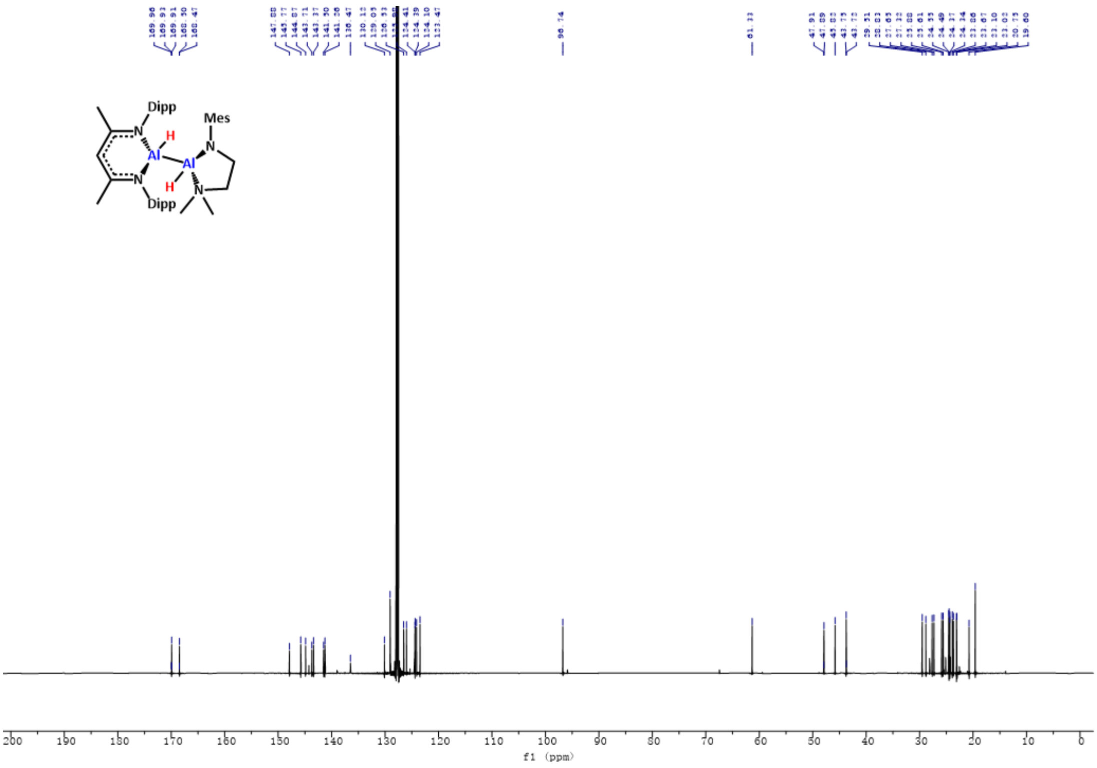

^{1}H NMR (C_6D_6, 400 MHz) of **4**:

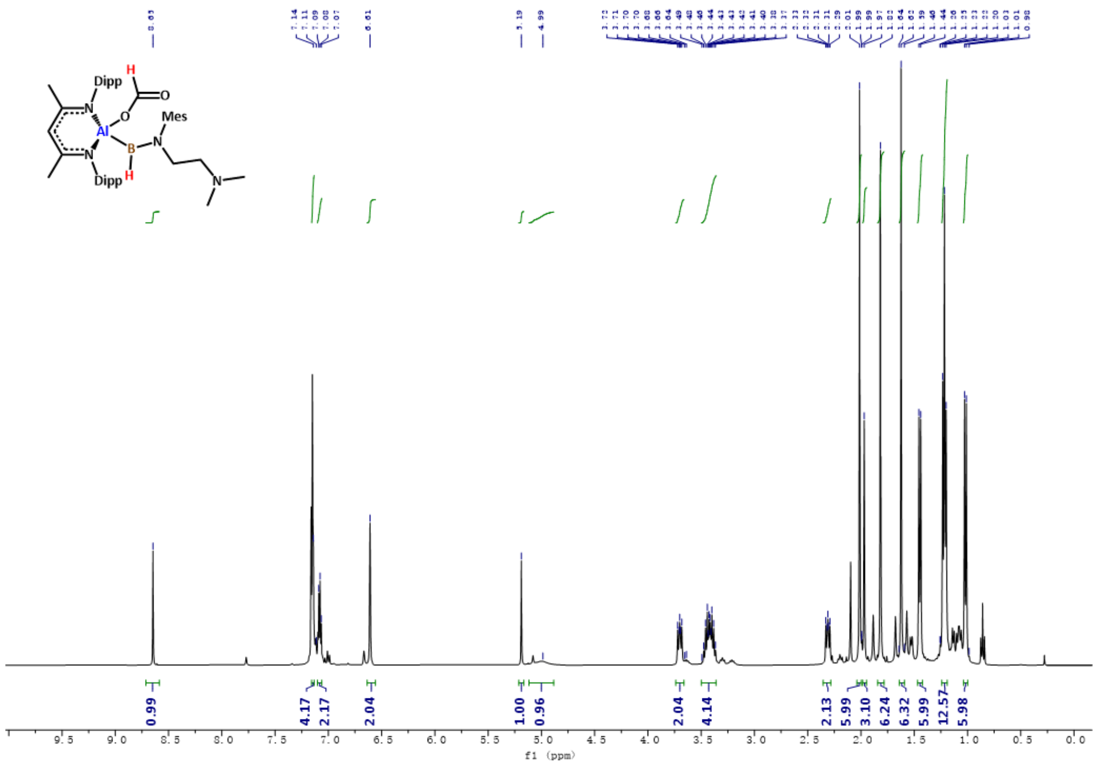

^{13}C NMR (C_6D_6, 101 MHz) of **4**:

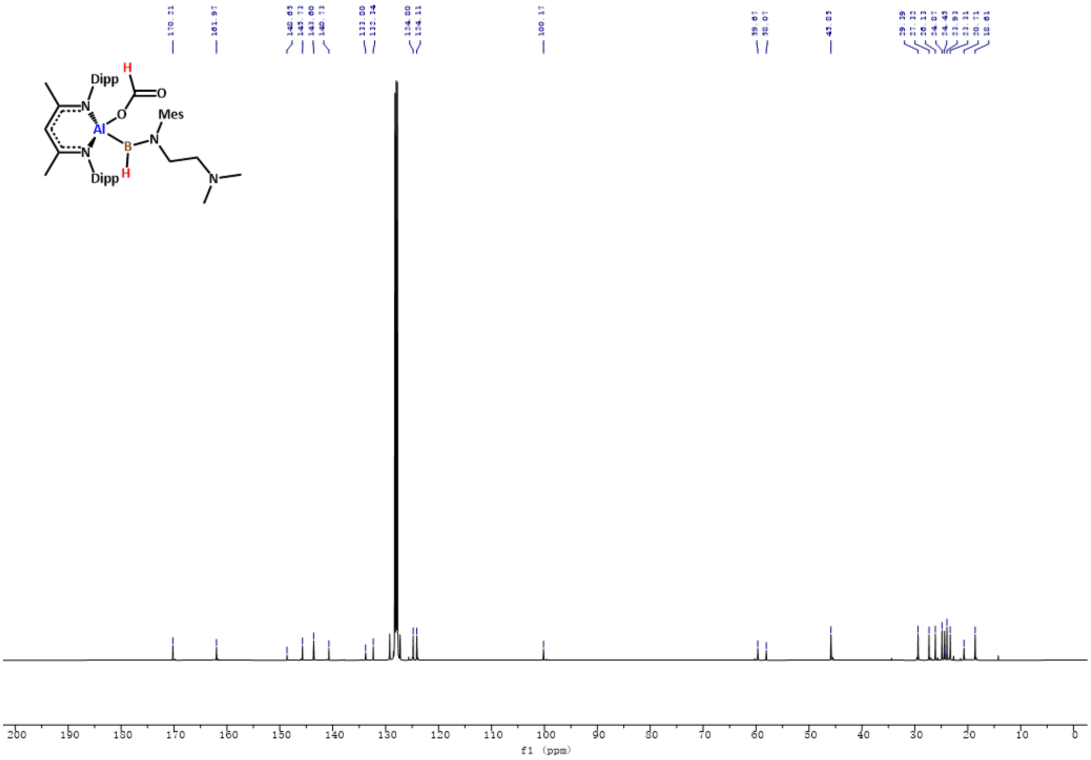

^{11}B NMR (C_6D_6, 128.0MHz) of **4**:

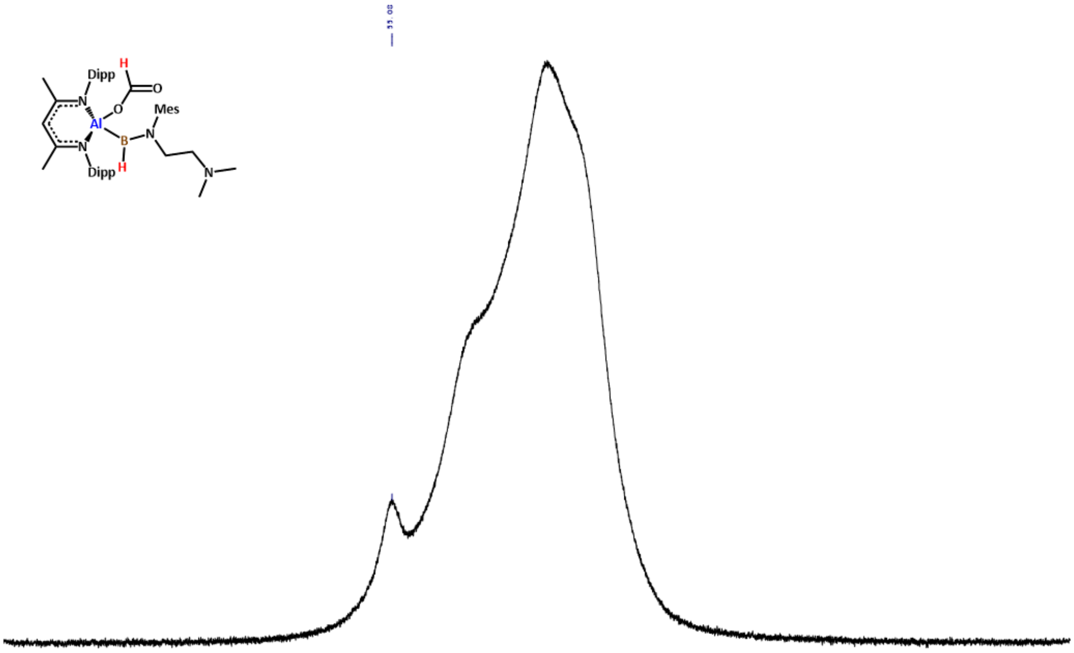

^{1}H NMR (C_6D_6, 400 MHz) of **5**: ("*"is the solvent peak of Et_2O)

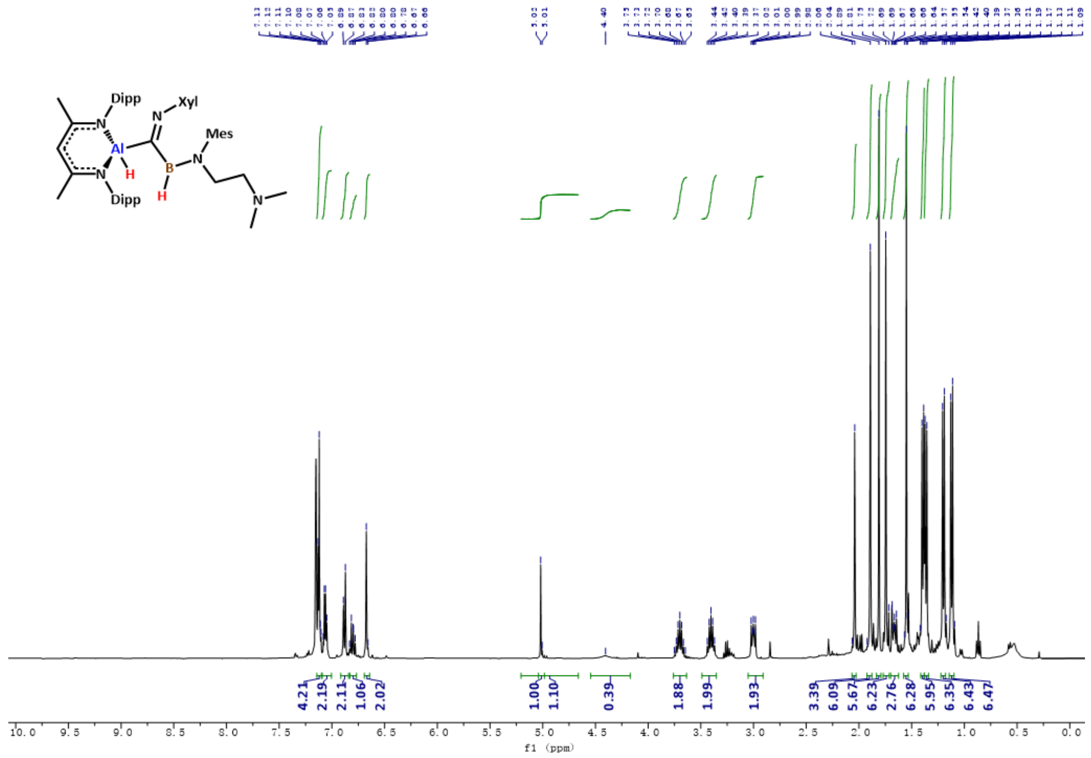

^{1}H NMR (C_6D_6, 400 MHz) of **6**:

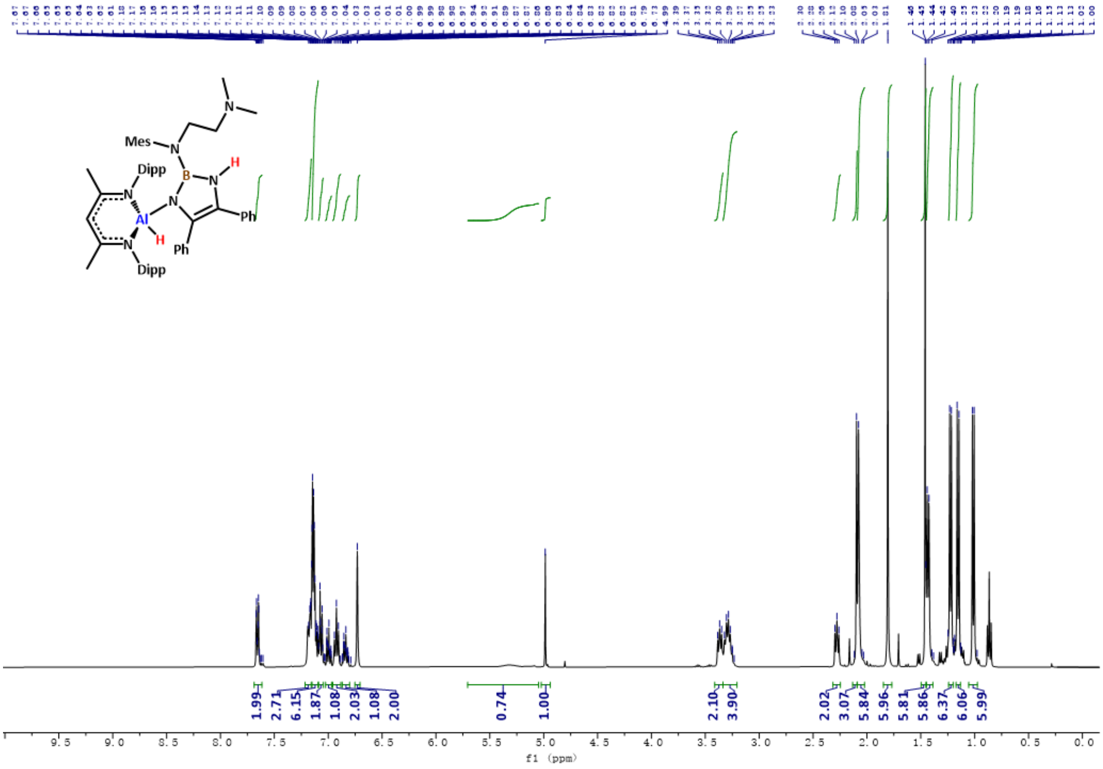

^{13}C NMR (C_6D_6, 101 MHz) of **6**:

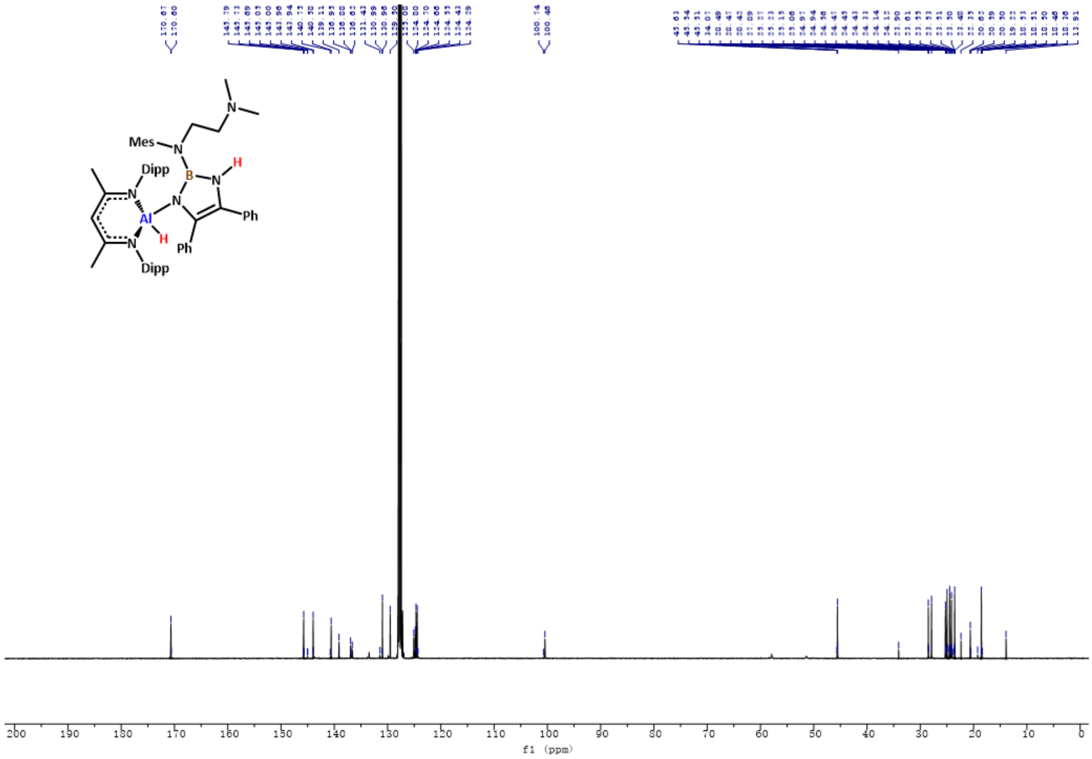

^{11}B NMR (C_6D_6, 128.0MHz) of **6**:

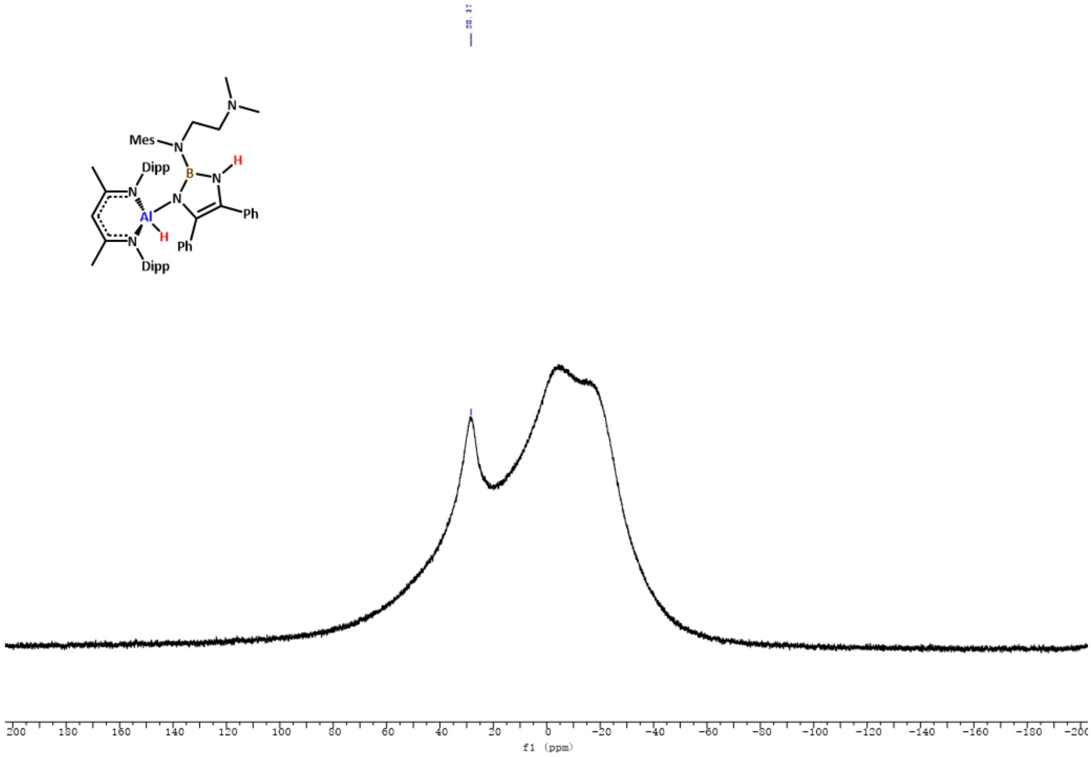

6) XYZ Coordinates

3a'.log

SCF (wB97x) = -1884.60937670

E(SCF)+ZPE(0 K)= -1883.622470

H(298 K)= -1883.568927

G(298 K)= -1883.703833

Lowest Frequency = 30.9454cm-1

Al	1.418732	4.970809	7.517075
H	0.047598	4.231696	7.021170
H	3.754349	4.223740	8.894382
N	2.504118	4.971632	5.846447
C	2.126364	5.679163	4.791812
C	1.111894	6.658114	4.847682
H	0.809428	7.083284	3.897736
C	0.619999	7.284992	5.994819
N	0.930162	6.887913	7.235843
C	2.778187	5.477006	3.444414
H	3.758936	5.007666	3.528722
H	2.878075	6.430613	2.921269
H	2.147628	4.826005	2.829446
C	-0.297930	8.467893	5.795058
H	-1.333497	8.137405	5.930568
H	-0.202473	8.882498	4.790212
H	-0.113909	9.258252	6.525382
C	3.556738	4.012912	5.725382
C	4.831653	4.340524	6.235818
C	5.853689	3.396653	6.115768
H	6.845323	3.627339	6.491789
C	5.617108	2.152253	5.536066
H	6.421048	1.424601	5.465656
C	4.349988	1.838442	5.061812
H	4.167994	0.860967	4.623818
C	3.299609	2.760638	5.132182
C	5.072840	5.701494	6.863725
H	4.152506	5.956733	7.401725
C	6.208939	5.711897	7.886776
H	7.190879	5.596152	7.413927
H	6.082896	4.909272	8.620362
H	6.219599	6.669500	8.418588
C	5.304806	6.778392	5.794741
H	5.500462	7.749168	6.263320
H	4.434911	6.895393	5.144606
H	6.168550	6.523296	5.170700
C	1.935806	2.399192	4.565615
H	1.315943	3.300271	4.568635
C	2.034071	1.896190	3.118853
H	2.597890	2.586047	2.483080
H	1.032684	1.775297	2.692089
H	2.529543	0.920419	3.067509
C	1.223218	1.373061	5.449091
H	0.228793	1.147134	5.048498
H	1.102400	1.745370	6.468361
H	1.790076	0.438888	5.503151
C	0.666301	7.768510	8.324981
C	-0.569330	7.718155	9.002073
C	-0.750790	8.540428	10.120418
H	-1.691806	8.500096	10.662711
C	0.247442	9.409119	10.543586
H	0.089143	10.040534	11.413654
C	1.448335	9.479148	9.841088
H	2.215216	10.172016	10.172776
C	1.683884	8.669335	8.726552
C	-1.699293	6.811815	8.541977
H	-1.394824	6.351561	7.596601
C	-2.991027	7.604185	8.297148
H	-3.749614	6.959690	7.840931
H	-2.822664	8.458536	7.634470
H	-3.408372	7.990744	9.233532
C	-1.947630	5.670788	9.534322
H	-2.203836	6.056588	10.528048
H	-1.063613	5.038441	9.630637
H	-2.772112	5.036670	9.192439
C	2.996476	8.755506	7.956517
H	3.225926	7.739851	7.613875
C	4.175742	9.261780	8.795065
H	4.270745	8.744587	9.753576
H	5.109135	9.116910	8.242346
H	4.089794	10.334309	9.002966
C	2.871798	9.634947	6.701434
H	3.851849	9.751527	6.226574
H	2.202745	9.198872	5.959234
H	2.500264	10.631657	6.964253
N	2.625849	5.070653	10.637317
C	3.244185	4.052045	11.538971

H	3.163867	4.364532	12.586499
H	4.299192	3.993973	11.260423
C	2.538102	2.739671	11.216723
H	3.238976	1.902309	11.366809
H	1.690699	2.556459	11.901957
N	2.092029	2.897202	9.841533
C	1.284555	5.441927	11.142434
H	0.873134	6.225789	10.508243
H	1.356303	5.808698	12.172716
H	0.641483	4.563827	11.097262
C	3.466481	6.272903	10.545075
H	4.436207	6.000037	10.128685
H	3.589725	6.730997	11.532579
H	2.971807	6.977978	9.881424
C	1.801757	1.684781	9.171765
C	2.835660	0.874415	8.654202
C	2.519069	-0.363189	8.091491
H	3.322738	-0.980548	7.692426
C	1.202084	-0.820647	8.008437
C	0.190022	-0.002824	8.512314
H	-0.844582	-0.336366	8.449956
C	0.468612	1.237780	9.093670
C	4.261694	1.347992	8.692877
H	4.940699	0.588867	8.295311
H	4.583188	1.598614	9.709789
H	4.381718	2.255973	8.097567
C	0.880513	-2.128744	7.335522
H	-0.044495	-2.566402	7.723675
H	1.684587	-2.859615	7.468658
H	0.745231	-1.986537	6.255556
C	-0.643249	2.110985	9.601322
H	-1.596219	1.573837	9.623741
H	-0.755206	2.981150	8.946549
H	-0.431278	2.487881	10.606737
B	2.570465	4.213420	9.222724

3a.log

SCF (wB97x) = -1884.60416254

E(SCF)+ZPE(0 K)= -1883.620442

H(298 K)= -1883.564831

G(298 K)= -1883.711171

Lowest Frequency = 7.8617cm-1

Al	4.505873	3.264336	5.128346
C	3.857122	5.135577	7.187830
N	3.976926	2.420673	2.137244
N	4.173236	2.203437	6.719797
N	3.724692	4.875795	5.891459
C	4.300988	2.767685	7.922711
C	3.923391	0.800190	6.605693
N	7.095642	3.453553	0.277598
C	3.131080	2.006571	1.052518
B	3.425291	2.639753	3.405413
C	2.594414	0.336244	6.710392
C	1.498748	2.531432	-0.639686
H	0.868813	3.262439	-1.143295
C	2.319907	2.958448	0.405748
C	4.099448	6.812081	4.427107
C	1.458355	1.197093	-1.053218
C	3.090961	0.652255	0.680634
C	6.410244	0.406987	6.168434
H	6.432461	1.468374	6.437391
C	3.590424	6.528285	7.697949
H	4.465034	7.158856	7.504283
H	3.398420	6.530149	8.771921
H	2.745599	6.986370	7.178590
C	0.953271	4.695264	5.122987
H	1.563457	3.794298	5.247457
C	6.410802	6.336752	3.594183
H	6.118515	5.318582	3.325547
H	7.476349	6.321038	3.847759
H	6.277498	6.974459	2.712782
C	4.985307	-0.087187	6.339901
C	5.721497	3.552303	0.751026
H	5.061376	3.374540	-0.105621
H	5.488924	4.563422	1.138163
C	4.251899	4.157113	8.123530
H	4.397744	4.501204	9.140278
C	4.497227	1.879530	9.123635
H	3.702260	1.133643	9.197569
H	4.532003	2.454131	10.049949
H	5.434636	1.323243	9.014107
C	5.577530	6.848415	4.776682
H	5.750025	6.159118	5.609347
C	2.253482	0.274232	-0.373625
H	2.214295	-0.775234	-0.660142
C	6.833014	0.295697	4.696638

H	6.881642	-0.753112	4.381910
H	7.817856	0.747479	4.538116
H	6.117068	0.803009	4.042996
C	0.590631	0.773643	-2.209267
H	-0.334307	1.357308	-2.251463
H	0.321184	-0.284703	-2.143075
H	1.110476	0.919520	-3.164332
C	8.066553	3.848512	1.287249
H	7.967643	4.909644	1.583171
H	9.078393	3.695498	0.898813
H	7.962250	3.236871	2.186865
C	2.254917	7.677606	3.106873
H	1.873712	8.391077	2.381394
C	4.696713	-1.447893	6.196601
H	5.504670	-2.143007	5.984323
C	3.224300	5.853126	4.975604
C	1.872508	5.772994	4.569519
C	-0.193191	4.342841	4.172558
H	-0.914885	5.162124	4.079776
H	-0.739934	3.476263	4.551560
H	0.176665	4.093101	3.173810
C	3.898399	-0.388920	1.410905
H	4.904199	-0.491229	0.985532
H	3.417173	-1.368489	1.343062
H	4.014248	-0.135499	2.468684
C	7.282256	4.225539	-0.939536
H	6.592467	3.874055	-1.713255
H	8.303542	4.089901	-1.310064
H	7.113469	5.310570	-0.793649
C	2.358120	-1.034986	6.577272
H	1.342370	-1.410818	6.668157
C	7.399779	-0.323634	7.083528
H	7.097394	-0.256997	8.133599
H	8.398931	0.114321	6.988035
H	7.480321	-1.385832	6.827979
C	2.314215	4.401152	0.835968
H	2.101301	4.508477	1.903276
H	1.562485	4.969625	0.282019
H	3.285444	4.878318	0.672724
C	1.430988	1.282177	6.956581
H	1.820218	2.304543	6.923019
C	3.588924	7.717575	3.490724
H	4.251386	8.459258	3.052704
C	6.043605	8.243573	5.211077

H	6.001835	8.959066	4.382708
H	7.081445	8.205403	5.558015
H	5.425982	8.642625	6.021946
C	5.404277	2.511514	1.824114
H	5.778118	1.541902	1.472775
H	5.919864	2.746380	2.758159
C	0.803191	1.066406	8.340398
H	0.394446	0.054045	8.434214
H	-0.016185	1.775358	8.502148
H	1.531312	1.208675	9.144005
C	3.396698	-1.924713	6.322533
H	3.191771	-2.986575	6.216774
C	1.409393	6.709758	3.641672
H	0.375249	6.675340	3.316204
C	0.404617	5.066754	6.507746
H	1.204101	5.181507	7.243274
H	-0.267574	4.281985	6.872288
H	-0.160004	6.004921	6.463963
C	0.367596	1.153841	5.860088
H	0.791224	1.360472	4.873318
H	-0.446851	1.862357	6.042091
H	-0.071601	0.150743	5.839469
H	6.108262	3.530812	5.110065
H	2.233656	2.469195	3.442982

3b.log

SCF (wB97x) = -1861.81288236

E(SCF)+ZPE(0 K)= -1860.832553

H(298 K)= -1860.777747

G(298 K)= -1860.916636

Lowest Frequency = 17.0029cm-1

Al	1.391723	5.124006	7.261149
Al	2.784398	4.514251	9.367188
H	0.006744	4.325226	6.965982
H	4.335853	4.980580	9.526914
N	2.400857	5.030333	5.581907
C	2.055241	5.754283	4.522240
C	1.171306	6.849447	4.591849
H	0.925724	7.326085	3.650896
C	0.721851	7.494329	5.752444
N	0.919766	7.003773	6.976689
C	2.633598	5.415877	3.171556

H	3.700342	5.191048	3.244298	C	1.749112	8.602903	8.618938
H	2.485696	6.230138	2.460851	C	-1.725062	6.964273	8.227021
H	2.146029	4.518836	2.776990	H	-1.427893	6.581183	7.245251
C	-0.008960	8.803204	5.598289	C	-2.985037	7.820761	8.042469
H	-1.061774	8.665845	5.868011	H	-3.762082	7.244554	7.529711
H	0.042277	9.174948	4.574257	H	-2.779938	8.719819	7.452599
H	0.389755	9.563245	6.274306	H	-3.397491	8.144259	9.004447
C	3.386275	4.000140	5.456664	C	-2.021004	5.745926	9.110624
C	4.715762	4.280443	5.848528	H	-2.251408	6.048282	10.138960
C	5.663931	3.262577	5.713206	H	-1.168110	5.063006	9.131348
H	6.693812	3.447032	5.998921	H	-2.878627	5.188789	8.719464
C	5.307428	1.999075	5.248958	C	3.082462	8.681180	7.892336
H	6.059275	1.218511	5.171042	H	3.062845	7.940863	7.086670
C	3.987908	1.732024	4.911311	C	4.267736	8.339955	8.800804
H	3.708746	0.735360	4.581651	H	4.210686	7.310797	9.165879
C	3.005446	2.724203	4.996063	H	5.204826	8.442708	8.244132
C	5.092864	5.640168	6.419849	H	4.326542	9.011173	9.664791
H	4.266300	5.940070	7.076553	C	3.275482	10.063066	7.251344
C	6.356479	5.596000	7.280804	H	4.214016	10.093317	6.687427
H	7.255194	5.425001	6.676748	H	2.462159	10.307049	6.562027
H	6.289263	4.816822	8.044732	H	3.315244	10.848968	8.014102
H	6.488308	6.552597	7.793775	N	2.196629	5.227714	11.257835
C	5.233268	6.719971	5.337088	C	2.698946	4.102732	12.092482
H	5.554198	7.665429	5.788824	H	2.381452	4.240340	13.136295
H	4.290506	6.910360	4.820296	H	3.792350	4.150288	12.051409
H	5.984086	6.429345	4.593572	C	2.223788	2.770685	11.521535
C	1.566819	2.394038	4.636635	H	2.831510	1.972451	11.977892
H	0.995227	3.327470	4.624581	H	1.184463	2.580622	11.843116
C	1.444683	1.744359	3.251956	N	2.380150	2.821522	10.082318
H	1.933928	2.338430	2.473113	C	0.733294	5.360581	11.368055
H	0.389888	1.628602	2.981442	H	0.402921	6.195498	10.747915
H	1.896487	0.746484	3.236307	H	0.438371	5.547430	12.409767
C	0.946460	1.494714	5.710549	H	0.253308	4.446997	11.016102
H	-0.107983	1.296926	5.488819	C	2.843280	6.487222	11.660511
H	1.003521	1.953819	6.700159	H	3.923153	6.398752	11.525449
H	1.471662	0.537138	5.768919	H	2.620343	6.715586	12.711688
C	0.686328	7.814385	8.125528	H	2.472327	7.300117	11.033808
C	-0.565720	7.781702	8.773069	C	2.191258	1.612442	9.388025
C	-0.726277	8.535131	9.941704	C	3.291596	0.987992	8.757181
H	-1.680278	8.512925	10.461684	C	3.112005	-0.231629	8.101715
C	0.309925	9.315760	10.443765	H	3.969893	-0.700137	7.621796
H	0.163795	9.899433	11.348463	C	1.871074	-0.869468	8.053695
C	1.531983	9.355085	9.777548	C	0.785982	-0.229292	8.654082
H	2.333157	9.979258	10.165007	H	-0.201134	-0.685295	8.590617

C	0.917693	1.003824	9.297318
C	4.648068	1.635772	8.798658
H	5.427937	0.943257	8.469012
H	4.883446	1.993572	9.805632
H	4.695224	2.506905	8.134738
C	1.691665	-2.166940	7.309965
H	0.918852	-2.791821	7.769025
H	2.620475	-2.745357	7.279344
H	1.385687	-1.986606	6.270903
C	-0.301575	1.715761	9.814490
H	-1.210996	1.331239	9.344239
H	-0.222845	2.784691	9.589840
H	-0.425024	1.615781	10.899953

4.log

SCF (wB97x) = -2073.22546591

E(SCF)+ZPE(0 K)= -2072.223212

H(298 K)= -2072.164746

G(298 K)= -2072.316051

Lowest Frequency = 11.6281cm-1

Al	0.333960	7.647824	6.130368
H	-0.823104	4.998737	7.434783
N	-0.635614	8.792320	4.938772
C	-1.099193	9.973479	5.342199
C	-0.724483	10.563169	6.566527
H	-1.241641	11.478033	6.830175
C	0.315800	10.168980	7.417774
N	1.028699	9.053515	7.215969
C	-2.081499	10.725562	4.484694
H	-1.981419	11.803288	4.625609
H	-3.098560	10.441364	4.775618
H	-1.962591	10.484201	3.426866
C	0.629147	11.025058	8.615503
H	0.170513	12.011980	8.541211
H	1.705233	11.135959	8.763582
H	0.236448	10.525049	9.508815
C	-0.945793	8.246248	3.649142
C	0.028567	8.340607	2.629233
C	-0.261507	7.772526	1.386549
H	0.466861	7.838711	0.585170
C	-1.464371	7.109490	1.159915
H	-1.669438	6.673188	0.186218
C	-2.392567	6.992792	2.186241
H	-3.319648	6.453391	2.012168
C	-2.153122	7.547423	3.448879
C	1.344737	9.060688	2.870293
H	1.586585	8.943733	3.931841
C	2.514969	8.468063	2.082794
H	2.627269	7.398843	2.282622
H	3.445895	8.959814	2.375429
H	2.401234	8.609906	1.002679
C	1.203190	10.565779	2.607259
H	0.928178	10.752042	1.563080
H	2.148161	11.081764	2.809100
H	0.435731	11.012824	3.245009
C	-3.164842	7.334078	4.561140
H	-2.805980	7.848020	5.457577
C	-3.265303	5.842315	4.908747
H	-3.890198	5.692196	5.793752
H	-2.276570	5.426934	5.121223
H	-3.693964	5.268163	4.079996
C	2.301031	8.870616	7.849339
C	2.411816	8.088639	9.014126
C	3.682138	7.900150	9.569633
H	3.784998	7.295996	10.466990
C	4.810109	8.472887	8.995197
H	5.787663	8.323650	9.445485
C	4.685264	9.231612	7.834885
H	5.572686	9.668250	7.386294
C	3.441077	9.433534	7.232303
C	1.205831	7.420974	9.647800
H	0.310948	7.763926	9.119130
C	1.037488	7.797822	11.124251
H	1.869924	7.429914	11.734014
H	0.115727	7.361736	11.522520
H	0.985208	8.883604	11.252832
C	1.297952	5.898299	9.473763
H	1.419640	5.630264	8.417999
H	0.396246	5.406978	9.853165
H	2.160238	5.492851	10.014547
C	3.331820	10.247077	5.953209
H	2.363400	10.011688	5.500676
C	4.419348	9.887258	4.936343
H	5.416640	10.177583	5.283488
H	4.240911	10.420002	3.996885
H	4.428076	8.813902	4.727362

C	3.348793	11.755360	6.239814
H	3.279199	12.322199	5.304954
H	4.278617	12.043121	6.743462
H	2.885722	7.148376	5.017302
B	1.871570	6.537190	5.224840
N	1.886912	5.219906	4.769314
C	3.062611	4.707087	4.122138
C	3.042984	4.453266	2.739565
C	4.208611	3.972618	2.135249
H	4.200779	3.784105	1.063169
C	5.377657	3.741046	2.860085
C	5.369946	4.022758	4.228772
H	6.277355	3.864328	4.808455
C	4.233872	4.508357	4.878964
C	1.819870	4.710064	1.897174
H	1.213940	3.802775	1.782908
H	2.107900	5.032474	0.892369
H	1.179213	5.484350	2.327167
C	6.608447	3.185083	2.193594
H	6.629773	3.423779	1.126113
H	6.643648	2.092475	2.285248
H	7.523057	3.578296	2.648069
C	4.292616	4.849344	6.345449
H	4.356066	5.933234	6.497930
H	5.168288	4.393275	6.815258
H	3.400018	4.515352	6.881130
C	0.782843	4.271366	4.919288
H	0.562893	3.785657	3.963127
H	-0.100110	4.849739	5.201591
C	1.098701	3.180853	5.943798
H	1.125768	3.627380	6.959572
H	2.107973	2.808582	5.733356
N	0.188103	2.049840	5.866133
C	0.691595	0.923389	6.635592
H	1.683114	0.637266	6.270989
H	0.770202	1.140583	7.718374
C	-1.167150	2.386070	6.273104
H	-1.573015	3.185943	5.648527
H	-1.232679	2.705933	7.330596
H	-1.814056	1.512915	6.145292
H	2.513077	12.057713	6.875540
H	0.024468	0.064633	6.510892
C	-4.547858	7.893100	4.202401
H	-4.994078	7.345548	3.364868
H	-5.226564	7.802001	5.056516
H	-4.497909	8.947428	3.915131
O	-1.031701	6.994605	7.137766
O	-2.396793	5.784124	8.418490
C	-1.435138	5.887566	7.685723

5.log

SCF (wB97x) = -2287.65730720

E(SCF)+ZPE(0 K)= -2286.513584

H(298 K)= -2286.449017

G(298 K)= -2286.610817

Lowest Frequency = 17.7788cm-1

Al	11.088766	10.604592	5.173057
H	11.146153	9.762516	6.542338
N	10.654167	12.440280	5.558974
C	11.593462	13.265362	6.024236
C	12.965229	12.970820	5.963866
H	13.631956	13.687621	6.427963
N	12.897193	10.972296	4.623585
C	13.581479	11.948276	5.217426
C	11.177830	14.593743	6.594514
H	10.867458	15.265222	5.788376
H	11.998212	15.066176	7.136511
H	10.319030	14.480132	7.260621
C	15.085930	11.973134	5.130217
H	15.494579	11.486002	6.022814
H	15.464304	12.996981	5.106773
H	15.453893	11.429669	4.258912
C	9.293973	12.866808	5.425440
C	8.912063	13.599559	4.281487
C	7.559180	13.912837	4.121048
H	7.243986	14.466183	3.240623
C	6.608815	13.509579	5.052662
H	5.560346	13.750668	4.900986
C	7.006268	12.804127	6.182942
H	6.263583	12.506730	6.918353
C	8.349247	12.480315	6.399351
C	9.913118	14.046356	3.227967
H	10.914007	13.755831	3.563622
C	9.898444	15.571588	3.049447
H	8.952589	15.910667	2.613099
H	10.702834	15.882386	2.374349

H	10.028177	16.100028	3.999103	C	9.852021	9.751443	3.831955
C	9.664797	13.351305	1.883654	B	8.727221	8.874896	4.484435
H	9.790454	12.267886	1.963366	H	7.700734	9.399803	4.804662
H	10.371726	13.723723	1.135023	N	8.913429	7.542873	4.856346
H	8.653352	13.554631	1.515409	C	10.118906	6.773670	4.551921
C	8.756911	11.778212	7.685336	H	10.533406	6.344191	5.469939
H	9.842058	11.634600	7.660754	H	10.858299	7.470932	4.148651
C	8.431884	12.646156	8.910148	C	9.859891	5.619023	3.583438
H	8.891685	13.636878	8.838477	H	8.943479	5.110396	3.904507
H	8.798629	12.167128	9.824241	H	9.672219	6.015712	2.568781
H	7.351335	12.789789	9.019625	N	10.933626	4.633051	3.611463
C	8.112529	10.393857	7.820450	C	7.889931	6.849342	5.580560
H	7.019874	10.462786	7.859893	C	8.077870	6.546668	6.940362
H	8.452638	9.902942	8.738010	C	7.061388	5.864855	7.616674
H	8.371510	9.747508	6.979325	H	7.200915	5.632354	8.670989
C	13.525390	10.023718	3.753729	C	5.877359	5.484504	6.985050
C	14.109138	8.860013	4.292684	C	5.716701	5.809811	5.635641
C	14.623708	7.909939	3.402542	H	4.798375	5.524057	5.125764
H	15.068150	6.999269	3.795171	C	6.700560	6.491474	4.916869
C	14.587121	8.120484	2.030154	C	9.328969	6.942001	7.681068
H	14.995101	7.374399	1.353856	H	9.099203	7.171093	8.725661
C	14.029196	9.290111	1.520379	H	9.807173	7.819821	7.238544
H	14.011026	9.441151	0.446241	H	10.064693	6.128176	7.682688
C	13.469885	10.254852	2.360688	C	4.784380	4.775756	7.741578
C	14.233457	8.631274	5.791156	H	5.175058	4.258949	8.623077
H	13.844178	9.517144	6.302943	H	4.276698	4.037520	7.112638
C	15.702889	8.456038	6.201484	H	4.022565	5.485129	8.087656
H	16.118946	7.524635	5.802407	C	6.468612	6.860903	3.476432
H	15.791684	8.415990	7.292304	H	6.192066	7.916717	3.382225
H	16.324903	9.278283	5.835321	H	5.659544	6.265287	3.044805
C	13.394036	7.438922	6.263155	H	7.361836	6.735106	2.859261
H	12.333933	7.608501	6.066510	C	12.204318	5.151868	3.131293
H	13.512508	7.291523	7.341996	H	12.189794	5.410003	2.057184
H	13.695983	6.512302	5.762730	H	12.985913	4.401212	3.289432
C	12.852122	11.531411	1.809475	H	12.494011	6.048759	3.682835
H	12.027599	11.795576	2.479507	C	10.558887	3.434577	2.881926
C	12.256543	11.372765	0.410968	H	9.639559	3.014214	3.301933
H	13.013637	11.116086	-0.338330	H	11.349182	2.682462	2.976408
H	11.804944	12.316588	0.093538	H	10.391220	3.619852	1.802752
H	11.472423	10.613964	0.410185	N	9.986651	9.949371	2.561684
C	13.862299	12.688439	1.823290	C	9.060556	9.303769	1.700755
H	14.732848	12.451719	1.200862	C	9.534644	8.243340	0.896013
H	14.218956	12.912385	2.830602	C	8.640737	7.583362	0.053185
H	13.399018	13.598891	1.427000	H	9.000113	6.747212	-0.542714

C	7.308107	7.983612	-0.039935
H	6.624897	7.464790	-0.706598
C	6.865633	9.054578	0.724726
H	5.829450	9.379284	0.654146
C	7.717502	9.726822	1.611352
C	10.980037	7.831614	0.939186
H	11.601464	8.519902	0.358623
H	11.112525	6.831070	0.518893
H	11.390739	7.841316	1.950544
C	7.154876	10.868336	2.418530
H	6.330100	10.524829	3.052784
H	6.756528	11.647362	1.759143
H	7.888754	11.333817	3.073559

6.log

SCF (wB97x) = -2533.60231694

E(SCF)+ZPE(0 K)= -2532.406362

H(298 K)= -2532.338980

G(298 K)= -2532.507610

Lowest Frequency = 18.1237cm-1

Al	1.161467	8.034916	5.132162
H	0.950169	6.525260	5.535674
N	-0.531509	8.839062	4.741750
C	-0.990159	9.952000	5.318379
C	-0.381392	10.537658	6.437699
H	-0.852279	11.433594	6.823938
C	0.713047	10.047772	7.174080
N	1.480812	9.037197	6.759904
C	-2.195456	10.636968	4.736040
H	-1.844362	11.275599	3.915735
H	-2.699305	11.267531	5.470336
H	-2.908564	9.925829	4.316458
C	0.974625	10.700061	8.507746
H	0.691410	11.754418	8.479783
H	2.015805	10.611984	8.818654
H	0.361061	10.214100	9.273031
C	-1.396279	8.027457	3.930581
C	-1.279973	8.026167	2.527909
C	-2.100440	7.159410	1.796815
H	-2.024646	7.149553	0.712891
C	-3.016908	6.327664	2.429022
H	-3.648362	5.665550	1.843150
C	-3.119877	6.343463	3.816374
H	-3.834873	5.689113	4.307884
C	-2.309492	7.175669	4.592353
C	-0.337759	8.965984	1.804756
H	0.224971	9.515640	2.561895
C	0.672206	8.212088	0.933427
H	1.264353	7.519627	1.536562
H	1.362936	8.919899	0.465187
H	0.175731	7.648505	0.135426
C	-1.118345	9.995375	0.977100
H	-1.667213	9.515575	0.158538
H	-0.429026	10.728183	0.546687
H	-1.843483	10.534168	1.594894
C	-2.443428	7.155486	6.106731
H	-1.664506	7.799692	6.523871
C	-2.215300	5.754228	6.680349
H	-2.283944	5.779013	7.773318
H	-1.222866	5.385518	6.408335
H	-2.960377	5.037771	6.318215
C	2.499550	8.523156	7.637503
C	2.158920	7.558409	8.607578
C	3.157138	7.127152	9.488156
H	2.910758	6.392478	10.249871
C	4.453134	7.617816	9.406623
H	5.210896	7.279071	10.107766
C	4.784257	8.523611	8.405025
H	5.807569	8.874922	8.323588
C	3.829379	8.977750	7.491008
C	0.770188	6.949422	8.719434
H	0.145901	7.372579	7.926285
C	0.107258	7.269141	10.065744
H	0.654256	6.808604	10.895714
H	-0.916859	6.881221	10.086941
H	0.066593	8.345299	10.255584
C	0.830377	5.431717	8.499292
H	1.203518	5.201731	7.498494
H	-0.164048	4.988286	8.602759
H	1.486988	4.947532	9.229558
C	4.231556	9.925729	6.374391
H	3.535937	9.766764	5.548364
C	5.626362	9.623319	5.819582
H	6.419614	9.850814	6.540091
H	5.810764	10.230695	4.930415
H	5.712088	8.573062	5.525047

C	4.119945	11.396169	6.794573
H	4.417242	12.049280	5.967256
H	4.770540	11.608413	7.650737
N	2.455025	8.246051	3.795616
C	3.060154	9.385115	3.215716
C	4.312179	9.077529	2.735977
N	4.534032	7.720298	2.937232
H	5.412925	7.281451	2.723269
B	3.363545	7.144304	3.539664
C	2.365579	10.667304	3.022742
C	1.686104	11.305418	4.065743
H	1.720095	10.881341	5.059557
C	0.975107	12.484458	3.853207
H	0.445950	12.940633	4.685518
C	0.950454	13.070046	2.588939
H	0.397238	13.989369	2.418486
C	1.649664	12.463260	1.542418
H	1.640902	12.909055	0.551342
C	2.339107	11.274031	1.753492
H	2.861762	10.793727	0.932502
C	5.320518	9.929619	2.105555
C	5.474080	11.281002	2.467069
H	4.820742	11.702318	3.224250
C	6.449016	12.072794	1.871700
H	6.547702	13.113506	2.168823
C	7.309220	11.534950	0.911495
H	8.075580	12.153667	0.453270
C	7.177560	10.194624	0.551299
H	7.837790	9.763970	-0.196898
C	6.193925	9.401482	1.138383
H	6.083351	8.366835	0.823486
N	3.172455	5.730867	3.733958
C	4.356340	4.949641	3.864255
C	4.991110	4.365507	2.751191
C	6.168786	3.636833	2.953087
H	6.661101	3.188095	2.091650
C	6.737587	3.484003	4.217921
C	6.103095	4.095396	5.303723
H	6.536270	3.997813	6.297777
C	4.924113	4.825263	5.149566
C	4.448085	4.535763	1.356214
H	3.702012	3.767945	1.118256
H	5.248872	4.451616	0.615456
H	3.965995	5.510468	1.236009

C	7.987292	2.666407	4.415243
H	7.741885	1.640801	4.718012
H	8.624840	3.090590	5.197454
H	8.576055	2.602656	3.495177
C	4.265994	5.490831	6.325110
H	4.353566	6.581316	6.267932
H	4.713075	5.175476	7.270955
H	3.193922	5.281657	6.349069
C	1.942622	5.060071	3.331634
H	2.031504	4.605555	2.333533
H	1.165724	5.825998	3.258695
C	1.519404	3.964989	4.305137
H	1.203116	4.432996	5.256505
H	2.395182	3.341917	4.523516
N	0.481648	3.100797	3.754831
C	0.227809	1.980681	4.643956
H	1.151268	1.414289	4.802571
H	-0.155885	2.294998	5.635030
C	-0.749906	3.826831	3.482463
H	-0.588002	4.619303	2.748262
H	-1.181289	4.295363	4.384050
H	-1.495140	3.141930	3.065082
H	3.096654	11.662972	7.071750
H	-0.509569	1.307737	4.193677
C	-3.798003	7.724094	6.550092
H	-4.624677	7.111702	6.173092
H	-3.866685	7.744619	7.643204
H	-3.943319	8.743967	6.181606

CNXyl.log

SCF (wB97x) = -402.983048969

E(SCF)+ZPE(0 K)= -402.828689

H(298 K)= -402.818229

G(298 K)= -402.862964

Lowest Frequency = 110.2487cm-1

C	-0.770616	0.854676	0.000117
C	0.635517	0.893056	0.000689
C	1.371997	2.091472	0.000200
C	0.650303	3.285780	-0.000962
C	-0.742796	3.280880	-0.001586
C	-1.443811	2.076975	-0.001030
H	1.190885	4.228136	-0.001384

H	-1.285822	4.221565	-0.002483
H	-2.530196	2.080350	-0.001515
C	1.914179	-1.320997	0.002774
N	1.326863	-0.304514	0.001859
C	2.872898	2.064656	0.000884
H	3.257357	1.536956	0.880749
H	3.258142	1.535836	-0.877966
H	3.283501	3.076737	0.000423
C	-1.497949	-0.458521	0.000738
H	-1.232595	-1.056512	-0.878147
H	-1.233166	-1.055350	0.880585
H	-2.579760	-0.308164	0.000292

CO2.log

SCF (wB97x) = -188.559680902
E(SCF)+ZPE(0 K)= -188.547993
H(298 K)= -188.544417
G(298 K)= -188.568692
Lowest Frequency = 655.5842cm-1

C	-1.069336	-0.175781	0.000000
O	0.091761	-0.175781	0.000000
O	-2.230433	-0.175781	0.000000

Int-1.log

SCF (wB97x) = -2287.62501085
E(SCF)+ZPE(0 K)= -2286.483799
H(298 K)= -2286.418358
G(298 K)= -2286.582990
Lowest Frequency = 11.8362cm-1

Al	0.851687	7.708876	5.689407
H	0.337540	6.333457	6.326091
N	-0.733354	8.667772	5.100933
C	-1.162553	9.828228	5.599654
C	-0.524219	10.497238	6.652633
H	-0.975237	11.432314	6.965079
C	0.571679	10.057330	7.416447
N	1.276484	8.969259	7.124454
C	-2.381315	10.499391	5.016506
H	-2.052672	11.312331	4.359024
H	-2.995370	10.942553	5.803815
H	-2.992226	9.814485	4.428515
C	0.881425	10.860117	8.656002
H	0.838446	11.930778	8.439430
H	1.853599	10.615064	9.082935
H	0.116963	10.651777	9.412071
C	-1.582679	7.884948	4.250156
C	-1.422880	7.917817	2.850086
C	-2.282874	7.138022	2.069109
H	-2.189062	7.165850	0.987205
C	-3.261065	6.339183	2.650178
H	-3.921028	5.744427	2.024922
C	-3.380674	6.291553	4.034832
H	-4.136314	5.654093	4.485625
C	-2.544337	7.048072	4.859824
C	-0.396992	8.812319	2.178795
H	0.255803	9.203140	2.963497
C	0.480429	8.050263	1.180570
H	1.048981	7.258360	1.673289
H	1.185171	8.740609	0.709090
H	-0.113107	7.595915	0.380787
C	-1.070139	10.009433	1.493489
H	-1.713855	9.675575	0.671782
H	-0.313710	10.687505	1.084765
H	-1.691879	10.573528	2.194043
C	-2.715516	6.981822	6.369329
H	-1.902320	7.553594	6.825726
C	-2.601350	5.547808	6.897326
H	-2.681204	5.542073	7.989931
H	-1.634449	5.116148	6.625334
H	-3.395077	4.902846	6.504670
C	2.350526	8.534613	7.971130
C	2.083206	7.620104	9.009546
C	3.157215	7.166233	9.782925
H	2.969429	6.464697	10.591232
C	4.454956	7.596403	9.536664
H	5.275575	7.233078	10.149123
C	4.703091	8.486781	8.496178
H	5.721260	8.809771	8.301450
C	3.667204	8.963320	7.689013
C	0.677756	7.133473	9.322173
H	0.002937	7.540961	8.562614
C	0.210194	7.645547	10.691665
H	0.824894	7.230028	11.497976
H	-0.828307	7.350827	10.877849

H	0.274040	8.735775	10.756307
C	0.577472	5.605311	9.245981
H	0.892961	5.246916	8.263011
H	-0.457326	5.285394	9.407881
H	1.198312	5.122850	10.008871
C	3.964381	9.925157	6.551031
H	3.112976	9.876960	5.865596
C	5.221774	9.546131	5.760069
H	6.134647	9.726604	6.338165
H	5.285414	10.156704	4.853283
H	5.201932	8.495045	5.460678
C	4.075110	11.372301	7.052262
H	4.321575	12.046975	6.224411
H	4.868927	11.459959	7.802555
H	3.188983	7.485482	5.546726
B	2.586126	7.281730	4.510055
C	2.744361	10.514846	2.139578
C	2.000868	11.589530	2.667967
C	1.761797	12.678435	1.828333
H	1.185036	13.516515	2.210420
C	2.235684	12.699381	0.518499
H	2.028156	13.553595	-0.119222
C	2.979570	11.628421	0.026923
H	3.355751	11.649200	-0.992231
C	3.260450	10.521753	0.827525
N	2.652314	5.898522	3.947195
C	3.914090	5.257828	3.951333
C	4.622591	5.065205	2.744108
C	5.874930	4.446941	2.771241
H	6.414463	4.308882	1.834909
C	6.461013	4.021178	3.964707
C	5.756950	4.236215	5.151251
H	6.196028	3.912102	6.094075
C	4.497443	4.841424	5.171006
C	4.055477	5.554112	1.438453
H	3.420712	4.797306	0.960706
H	4.852371	5.805955	0.732137
H	3.433863	6.439565	1.599849
C	7.797411	3.325240	3.969100
H	7.678168	2.238146	3.877103
H	8.345780	3.513504	4.897557
H	8.424143	3.653846	3.133852
C	3.794064	5.046124	6.486155
H	4.064399	6.007705	6.940218

H	4.061014	4.260237	7.199868
H	2.709122	5.056718	6.358627
C	1.516662	5.178072	3.410627
H	1.666005	4.860820	2.365459
H	0.667066	5.869201	3.406753
C	1.170874	3.931113	4.231097
H	0.830983	4.252617	5.234927
H	2.092362	3.352539	4.369815
N	0.190352	3.068948	3.581007
C	0.063536	1.807683	4.289729
H	1.035117	1.304873	4.331955
H	-0.304082	1.930895	5.328090
C	-1.103785	3.719899	3.458225
H	-1.028442	4.631294	2.861934
H	-1.538986	3.999946	4.435984
H	-1.807666	3.051583	2.950971
H	3.144616	11.721346	7.505693
H	-0.635130	1.151053	3.759860
C	-4.039043	7.629924	6.798188
H	-4.895615	7.085738	6.384720
H	-4.133624	7.625565	7.889549
H	-4.105940	8.666515	6.454367
C	2.838983	8.460705	3.610111
N	2.987773	9.405439	2.914291
C	4.064478	9.356401	0.327727
H	4.874457	9.109785	1.022461
H	3.447172	8.455628	0.237844
H	4.497299	9.569175	-0.652350
C	1.500970	11.567245	4.082405
H	0.698822	12.293722	4.228572
H	1.114918	10.587106	4.367946
H	2.302374	11.809474	4.789340

Int-2.log

SCF (wB97x) = -2209.00482923

E(SCF)+ZPE(0 K)= -2207.919238

H(298 K)= -2207.857000

G(298 K)= -2208.015327

Lowest Frequency = 14.3399cm-1

Al	0.902136	7.638180	5.613934
H	0.304426	6.353016	6.364715
N	-0.626114	8.629216	4.912325

C	-0.971750	9.863688	5.272368
C	-0.311551	10.589782	6.275891
H	-0.714035	11.572754	6.493170
C	0.720197	10.146888	7.124907
N	1.364797	8.999629	6.950534
C	-2.114383	10.563580	4.580764
H	-1.699219	11.229049	3.815108
H	-2.679535	11.178966	5.284128
H	-2.792280	9.865206	4.089391
C	1.021540	11.021756	8.316691
H	1.023103	12.077515	8.033828
H	1.968677	10.771169	8.793708
H	0.224668	10.885213	9.055962
C	-1.496262	7.837224	4.090736
C	-1.332769	7.802161	2.690683
C	-2.198910	6.998639	1.942131
H	-2.093887	6.970244	0.860938
C	-3.196753	6.249326	2.555428
H	-3.862367	5.634046	1.956551
C	-3.333476	6.282371	3.939227
H	-4.111821	5.691737	4.414308
C	-2.487432	7.062261	4.732446
C	-0.294590	8.656038	1.990570
H	0.300051	9.132232	2.772688
C	0.651019	7.831918	1.109737
H	1.194851	7.086157	1.691674
H	1.384950	8.489732	0.631398
H	0.106846	7.314879	0.312343
C	-0.949843	9.770447	1.162349
H	-1.492163	9.353252	0.306237
H	-0.185759	10.456548	0.781547
H	-1.662580	10.349240	1.754885
C	-2.686883	7.104996	6.238872
H	-1.838775	7.642571	6.672861
C	-2.695016	5.707162	6.864975
H	-2.776381	5.784178	7.954745
H	-1.767663	5.177496	6.630786
H	-3.539460	5.104140	6.513623
C	2.428960	8.606100	7.828613
C	2.146871	7.764653	8.922688
C	3.213410	7.340323	9.722788
H	3.014115	6.693812	10.573111
C	4.517797	7.733585	9.451304
H	5.332906	7.394524	10.084728
C	4.779244	8.559515	8.361781
H	5.802002	8.857818	8.151843
C	3.751868	9.002647	7.525530
C	0.731259	7.335672	9.270140
H	0.066554	7.699573	8.480100
C	0.278137	7.966675	10.594208
H	0.886873	7.605484	11.430696
H	-0.765788	7.710070	10.805183
H	0.364910	9.057104	10.569251
C	0.593140	5.809445	9.314120
H	0.913747	5.365395	8.368314
H	-0.451827	5.528627	9.484206
H	1.190313	5.374275	10.123083
C	4.065148	9.898788	6.338447
H	3.207882	9.836798	5.662885
C	5.305627	9.448614	5.558103
H	6.225758	9.615106	6.128661
H	5.390057	10.027813	4.632586
H	5.247611	8.389622	5.294430
C	4.220170	11.367230	6.759879
H	4.438217	11.991650	5.886383
H	5.046371	11.478657	7.471159
N	2.862588	8.308426	3.547302
C	2.931499	9.322195	2.969470
H	3.376947	7.350727	5.409178
B	2.648797	7.140624	4.462829
C	2.489924	10.593604	2.507194
C	1.735433	11.380708	3.400052
H	1.520599	11.015517	4.400486
C	1.238327	12.605548	2.974623
H	0.649559	13.207201	3.661048
C	1.480009	13.051514	1.672653
H	1.083312	14.007453	1.344176
C	2.231588	12.270103	0.792577
H	2.419024	12.617359	-0.218875
C	2.748640	11.045901	1.204346
H	3.335766	10.432833	0.528326
N	2.718245	5.768244	3.908003
C	3.958156	5.084066	3.976586
C	4.686519	4.822957	2.794691
C	5.911042	4.155120	2.873090
H	6.466362	3.964145	1.955512
C	6.449998	3.745040	4.093737
C	5.726980	4.027639	5.254162

H	6.129626	3.717548	6.217739
C	4.493423	4.684293	5.223479
C	4.169485	5.286414	1.458694
H	3.500887	4.545535	1.002460
H	4.991795	5.458121	0.757433
H	3.596256	6.211816	1.569224
C	7.755656	2.995398	4.154546
H	7.593282	1.911554	4.096027
H	8.289602	3.192838	5.089499
H	8.414971	3.269087	3.324606
C	3.774801	4.959263	6.518290
H	4.087963	5.915801	6.954673
H	3.984430	4.176720	7.254618
H	2.694611	5.024749	6.370521
C	1.573569	5.038585	3.398003
H	1.732055	4.662203	2.374745
H	0.738596	5.745151	3.344004
C	1.189334	3.847474	4.281464
H	0.854102	4.230258	5.265180
H	2.092636	3.250469	4.455706
N	0.185530	2.981454	3.673435
C	0.028268	1.758047	4.440299
H	0.986309	1.231964	4.502784
H	-0.331709	1.938877	5.472899
C	-1.091566	3.659691	3.524078
H	-0.995344	4.539797	2.885483
H	-1.514472	3.997003	4.489219
H	-1.815400	2.987416	3.051421
H	3.316790	11.757095	7.233092
H	-0.689821	1.096623	3.943398
C	-3.961474	7.884438	6.591206
H	-4.849836	7.388935	6.183405
H	-4.083004	7.954039	7.677755
H	-3.931158	8.900751	6.186107

Int-3.log

SCF (wB97x) = -2209.03839246
E(SCF)+ZPE(0 K)= -2207.950746
H(298 K)= -2207.889170
G(298 K)= -2208.045037
Lowest Frequency = 20.3666cm-1

Al	1.046527	8.065462	5.275307
H	0.890601	6.512854	5.540042
N	-0.639967	8.855140	4.873929
C	-1.063427	9.992733	5.445695
C	-0.447198	10.564443	6.563439
H	-0.906635	11.464562	6.953366
C	0.611267	10.033861	7.331608
N	1.379350	9.030599	6.914117
C	-2.256743	10.693605	4.858437
H	-1.940004	11.163526	3.919610
H	-2.640331	11.467992	5.523772
H	-3.059880	9.994869	4.615138
C	0.804780	10.618677	8.706526
H	0.578287	11.687071	8.709669
H	1.810996	10.459603	9.093784
H	0.100192	10.132095	9.389771
C	-1.575831	8.061186	4.121094
C	-1.648412	8.139430	2.719246
C	-2.572607	7.321013	2.058492
H	-2.638535	7.366401	0.975032
C	-3.405752	6.458706	2.758434
H	-4.119460	5.836399	2.225778
C	-3.320049	6.392713	4.145376
H	-3.974427	5.718139	4.689991
C	-2.405344	7.178003	4.849785
C	-0.774390	9.075506	1.909476
H	-0.183311	9.676449	2.606056
C	0.196821	8.281664	1.026625
H	0.906255	7.711350	1.632333
H	0.775708	8.957772	0.392232
H	-0.338597	7.580034	0.377623
C	-1.612814	10.041374	1.061930
H	-2.163684	9.514555	0.274819
H	-0.958671	10.776461	0.584216
H	-2.342566	10.581125	1.673567
C	-2.353405	7.098790	6.366438
H	-1.445733	7.613030	6.695740
C	-2.264696	5.658004	6.874789
H	-2.172697	5.653145	7.965713
H	-1.391569	5.150206	6.455934
H	-3.156751	5.075977	6.621219
C	2.448638	8.534481	7.743832
C	2.194549	7.491605	8.656185
C	3.251464	7.040216	9.454628
H	3.072929	6.241678	10.169271

C	4.519997	7.592252	9.348706
H	5.327634	7.230251	9.978749
C	4.760130	8.599002	8.418532
H	5.760548	9.008141	8.327337
C	3.743888	9.085562	7.591791
C	0.827583	6.846792	8.814725
H	0.148206	7.303729	8.088643
C	0.250408	7.096335	10.215029
H	0.846055	6.591719	10.983520
H	-0.772582	6.710304	10.280424
H	0.230854	8.161943	10.460775
C	0.884394	5.343629	8.513214
H	1.166221	5.167077	7.472778
H	-0.091446	4.880612	8.684639
H	1.610032	4.834211	9.155504
C	4.041043	10.171258	6.568267
H	3.418932	9.955304	5.699288
C	5.492325	10.167090	6.078763
H	6.180868	10.563032	6.834448
H	5.575675	10.803712	5.192967
H	5.821755	9.163553	5.798807
C	3.673859	11.576880	7.065865
H	3.978246	12.323314	6.324965
H	4.183862	11.803361	8.008979
N	2.554829	8.313288	4.124124
C	3.055609	9.152880	3.237023
H	4.656635	7.604322	4.799282
B	3.659424	7.314041	4.216960
C	2.282922	10.362360	2.924677
C	1.612244	11.102635	3.915425
H	1.651487	10.780543	4.950139
C	0.907703	12.259433	3.593921
H	0.397491	12.809386	4.380633
C	0.848688	12.697253	2.270567
H	0.290243	13.593285	2.014394
C	1.537283	11.990996	1.279330
H	1.512503	12.338734	0.249956
C	2.272012	10.856863	1.608687
H	2.834376	10.319776	0.849889
N	3.529401	5.992729	3.761840
C	4.612745	5.083260	3.982566
C	5.408125	4.652652	2.906086
C	6.443380	3.747603	3.156664
H	7.063943	3.418959	2.324727
C	6.713768	3.270586	4.439989
C	5.926511	3.738566	5.494347
H	6.131815	3.391666	6.505588
C	4.878600	4.639865	5.293221
C	5.186269	5.173800	1.510877
H	4.450708	4.569741	0.965727
H	6.116599	5.147293	0.936492
H	4.819776	6.204512	1.529700
C	7.809916	2.265482	4.679148
H	7.422704	1.240530	4.620740
H	8.257354	2.387489	5.670427
H	8.606336	2.355094	3.934088
C	4.080953	5.141896	6.466657
H	4.426093	6.129772	6.793294
H	4.165778	4.465412	7.321423
H	3.024463	5.257290	6.216943
C	2.353561	5.471910	3.069578
H	2.584938	5.228765	2.025659
H	1.613128	6.273061	3.048748
C	1.776632	4.227769	3.741015
H	1.494949	4.472889	4.782284
H	2.563768	3.467260	3.790048
N	0.653799	3.671531	2.997641
C	0.357780	2.320710	3.444770
H	1.238051	1.682770	3.317273
H	0.050922	2.276024	4.508558
C	-0.535168	4.505935	3.089344
H	-0.350819	5.507458	2.695140
H	-0.900183	4.619653	4.126110
H	-1.340577	4.068277	2.492474
H	2.600376	11.697134	7.221389
H	-0.454465	1.901241	2.842078
C	-3.546024	7.833806	6.993440
H	-4.491953	7.374727	6.684873
H	-3.490351	7.794519	8.086834
H	-3.570114	8.885639	6.694065

Int-4.log

SCF (wB97x) = -2533.42714054

E(SCF)+ZPE(0 K)= -2532.237260

H(298 K)= -2532.169043

G(298 K)= -2532.340063

Lowest Frequency = 11.0570cm-1

Al	1.072368	8.103415	5.042465
H	0.992158	6.559848	5.385871
N	-0.731738	8.756842	4.897234
C	-1.140539	9.879420	5.504463
C	-0.401621	10.535867	6.495391
H	-0.856114	11.424438	6.915914
C	0.787989	10.106277	7.112645
N	1.549233	9.122450	6.633947
C	-2.460973	10.484280	5.111216
H	-2.337846	10.949058	4.126178
H	-2.777774	11.251336	5.818952
H	-3.246028	9.730672	5.021212
C	1.147802	10.779588	8.412401
H	0.803481	11.815866	8.417563
H	2.217689	10.750805	8.619126
H	0.638538	10.257694	9.229588
C	-1.729685	7.914242	4.285899
C	-2.068539	8.038678	2.926888
C	-3.082619	7.219226	2.415803
H	-3.349268	7.302885	1.366010
C	-3.759808	6.317175	3.223039
H	-4.554419	5.701743	2.810353
C	-3.405061	6.195589	4.562376
H	-3.930696	5.481084	5.187929
C	-2.380019	6.968082	5.113289
C	-1.391391	9.029056	2.002663
H	-0.728198	9.656992	2.603651
C	-0.536114	8.293028	0.963097
H	0.312086	7.788218	1.433710
H	-0.133128	8.997177	0.230899
H	-1.128565	7.545632	0.423506
C	-2.403237	9.951150	1.309456
H	-3.020691	9.405344	0.587357
H	-1.871148	10.739709	0.769615
H	-3.077078	10.425588	2.029687
C	-2.015650	6.803507	6.578925
H	-1.001523	7.192864	6.708976
C	-1.993550	5.335216	7.010443
H	-1.663227	5.256553	8.049414
H	-1.307291	4.754152	6.386813
H	-2.984624	4.873083	6.955443
C	2.706605	8.704263	7.383041
C	2.560264	7.773718	8.429252
C	3.704053	7.384369	9.135990
H	3.609164	6.661944	9.941942
C	4.952863	7.902269	8.824877
H	5.829153	7.592174	9.387592
C	5.080318	8.815218	7.781974
H	6.061972	9.208256	7.539271
C	3.973598	9.227377	7.035852
C	1.220810	7.174592	8.822004
H	0.442766	7.643847	8.211917
C	0.885773	7.449059	10.295205
H	1.559714	6.904706	10.965505
H	-0.135362	7.121916	10.518476
H	0.966442	8.511990	10.541288
C	1.194466	5.667196	8.537564
H	1.241724	5.472818	7.464153
H	0.277769	5.217328	8.929316
H	2.041112	5.162459	9.014437
C	4.141787	10.210255	5.889103
H	3.427275	9.918457	5.117351
C	5.528205	10.151924	5.242604
H	6.293083	10.629806	5.866071
H	5.504782	10.679646	4.284359
H	5.832174	9.120084	5.050767
C	3.821528	11.654193	6.300339
H	3.992560	12.329369	5.455517
H	4.460966	11.973770	7.131232
N	2.348738	8.373360	3.608959
C	2.547260	9.260384	2.656012
C	5.847880	7.618850	2.564724
N	4.772136	7.827394	2.933513
H	3.943491	7.465291	4.927944
B	3.457187	7.346830	3.820857
C	1.675794	10.438860	2.586563
C	1.251781	11.156681	3.719746
H	1.544627	10.818643	4.706502
C	0.474359	12.306425	3.599376
H	0.154044	12.828754	4.497375
C	0.098512	12.770234	2.339388
H	-0.514671	13.662009	2.242445
C	0.548440	12.095883	1.198111
H	0.283997	12.466105	0.210707
C	1.351304	10.968961	1.321466
H	1.735916	10.462557	0.440417
C	7.178335	7.287234	2.193206

C	7.777604	7.845339	1.054009
H	7.226426	8.555246	0.446121
C	9.075101	7.471228	0.723295
H	9.549602	7.894923	-0.156237
C	9.764289	6.552569	1.518418
H	10.777070	6.264293	1.252007
C	9.161371	5.999429	2.649803
H	9.697458	5.279018	3.259663
C	7.864792	6.359747	2.996147
H	7.367549	5.919343	3.854433
N	3.191051	5.932862	3.444017
C	4.253525	5.015951	3.637225
C	4.973125	4.493959	2.533883
C	6.046878	3.629971	2.759895
H	6.599656	3.249270	1.902479
C	6.463958	3.282534	4.045663
C	5.752693	3.813850	5.122196
H	6.046127	3.546631	6.136480
C	4.650634	4.657646	4.947090
C	4.645434	4.878692	1.114536
H	3.885055	4.217771	0.680937
H	5.537674	4.810940	0.484764
H	4.258670	5.899144	1.056336
C	7.633578	2.356608	4.253842
H	7.342356	1.308701	4.110535
H	8.043746	2.446956	5.264203
H	8.438374	2.566207	3.540806
C	3.895650	5.139051	6.154471
H	4.338257	6.044764	6.584102
H	3.885611	4.373777	6.937162
H	2.868632	5.393588	5.888064
C	2.018903	5.498914	2.704686
H	2.207188	5.277000	1.644586
H	1.311393	6.330714	2.721386
C	1.395484	4.263515	3.354624
H	1.075410	4.533035	4.380192
H	2.173728	3.496739	3.450656
N	0.300122	3.692164	2.580417
C	-0.134379	2.439514	3.174052
H	0.705070	1.738069	3.221147
H	-0.533335	2.564652	4.200806
C	-0.819781	4.614796	2.464147
H	-0.534685	5.504184	1.899516
H	-1.206314	4.947010	3.444299
H	-1.639683	4.133883	1.921920
H	2.780520	11.776886	6.606097
H	-0.920012	1.987894	2.559002
C	-2.946587	7.624727	7.481222
H	-3.985078	7.291916	7.372006
H	-2.660262	7.509078	8.532655
H	-2.905346	8.690064	7.238846

Int-5.log

SCF (wB97x) = -2533.52035963
E(SCF)+ZPE(0 K)= -2532.327881
H(298 K)= -2532.260770
G(298 K)= -2532.428684
Lowest Frequency = 17.6773cm-1

Al	1.156194	7.838354	5.351058
H	0.791241	6.405094	5.872129
N	-0.391319	8.757481	4.721855
C	-0.674641	10.030155	5.025613
C	-0.038872	10.721964	6.062411
H	-0.383959	11.732030	6.246871
C	0.903946	10.211892	6.977589
N	1.552820	9.060612	6.794133
C	-1.702110	10.761905	4.207490
H	-1.211305	11.092061	3.283494
H	-2.085817	11.641249	4.726997
H	-2.533743	10.115673	3.921038
C	1.117838	11.006472	8.239349
H	0.948324	12.070161	8.060910
H	2.109425	10.860631	8.667410
H	0.387202	10.675031	8.985041
C	-1.365798	7.945022	4.042502
C	-1.287103	7.732481	2.655941
C	-2.218952	6.870202	2.065479
H	-2.167997	6.686876	0.995788
C	-3.209720	6.256627	2.820397
H	-3.924950	5.592137	2.343940
C	-3.283367	6.491752	4.190634
H	-4.061609	6.007759	4.772416
C	-2.364308	7.324892	4.831215
C	-0.253038	8.429811	1.794259
H	0.330928	9.089953	2.442466
C	0.710441	7.422343	1.160201

H	1.281107	6.889871	1.922711	N	4.943691	6.986422	3.870720
H	1.421537	7.932281	0.503801	H	3.675620	6.491156	5.712006
H	0.174622	6.680558	0.558488	B	3.628611	6.520613	4.476369
C	-0.916457	9.305949	0.723602	C	2.796687	9.821128	2.836182
H	-1.456472	8.699852	-0.012064	C	2.164198	10.771508	3.641198
H	-0.154652	9.884199	0.191603	H	2.108444	10.617972	4.711385
H	-1.630753	10.007430	1.165776	C	1.615427	11.919664	3.076629
C	-2.468268	7.582263	6.326548	H	1.122690	12.644944	3.717485
H	-1.478630	7.899080	6.671156	C	1.695053	12.126221	1.699858
C	-2.857239	6.341742	7.134586	H	1.259196	13.016426	1.255741
H	-2.783056	6.560800	8.204725	C	2.349829	11.192496	0.893500
H	-2.201773	5.496894	6.912156	H	2.425115	11.354977	-0.177546
H	-3.890251	6.033733	6.940569	C	2.912955	10.052501	1.457982
C	2.451182	8.589305	7.820378	H	3.431813	9.330851	0.835314
C	1.946361	7.804025	8.874796	C	5.766709	8.738943	2.409385
C	2.833709	7.397572	9.877285	C	5.950993	10.128069	2.410817
H	2.462846	6.793798	10.700941	H	5.306233	10.758798	3.015523
C	4.175659	7.749390	9.835307	C	6.966693	10.703168	1.649769
H	4.848492	7.431653	10.626946	H	7.107947	11.780353	1.665490
C	4.663029	8.490872	8.763472	C	7.799486	9.898906	0.871839
H	5.719455	8.734021	8.724594	H	8.586668	10.349456	0.273469
C	3.823577	8.911527	7.729212	C	7.621684	8.514002	0.866496
C	0.495456	7.360157	8.950415	H	8.270338	7.883958	0.264129
H	-0.032660	7.776670	8.087521	C	6.615045	7.935540	1.634351
C	-0.203738	7.880006	10.213137	H	6.467685	6.858881	1.650756
H	0.233945	7.444618	11.117881	N	3.216000	5.286178	3.703087
H	-1.265610	7.611803	10.197108	C	4.220857	4.461637	3.135559
H	-0.128728	8.967892	10.301419	C	4.364513	4.404293	1.730961
C	0.391190	5.831104	8.862419	C	5.344815	3.582624	1.170550
H	0.838716	5.462590	7.935840	H	5.448927	3.550501	0.086602
H	-0.656776	5.517500	8.884338	C	6.212297	2.825393	1.961030
H	0.900909	5.352006	9.705189	C	6.089176	2.931129	3.347010
C	4.388882	9.683573	6.546945	H	6.772580	2.371389	3.984301
H	3.770656	9.424355	5.685575	C	5.116520	3.735549	3.947571
C	5.825740	9.278658	6.205202	C	3.529855	5.289506	0.847512
H	6.538588	9.633302	6.957835	H	2.492133	4.947004	0.763704
H	6.119649	9.722134	5.249419	H	3.944148	5.340275	-0.163723
H	5.920142	8.194377	6.107949	H	3.490313	6.300230	1.266125
C	4.299479	11.204569	6.732919	C	7.237699	1.915141	1.336723
H	4.758631	11.714405	5.878949	H	6.826320	0.912113	1.165225
H	4.829107	11.516861	7.639817	H	8.116779	1.799508	1.978659
N	2.642716	7.755435	4.149202	H	7.575307	2.295715	0.367138
C	3.318139	8.567070	3.408107	C	5.078276	3.871537	5.444542
C	4.729553	8.066670	3.226301	H	5.551105	4.813114	5.748818

H	5.610418	3.048946	5.932007
H	4.053744	3.908127	5.821663
C	1.864672	4.778765	3.626300
H	1.679347	4.322599	2.643639
H	1.165046	5.617224	3.708765
C	1.539704	3.727128	4.688197
H	1.637807	4.187661	5.691808
H	2.298248	2.938603	4.614756
N	0.230521	3.108629	4.505849
C	0.065272	1.984394	5.410307
H	0.861039	1.251520	5.242666
H	0.093453	2.282414	6.477787
C	-0.854050	4.061614	4.674243
H	-0.806254	4.856471	3.926685
H	-0.839524	4.540958	5.670101
H	-1.816188	3.555084	4.548881
H	3.267149	11.554699	6.806260
H	-0.894777	1.491467	5.222201
C	-3.448881	8.726561	6.624724
H	-4.450529	8.476876	6.257143
H	-3.515739	8.899817	7.704349
H	-3.142491	9.663520	6.155053

Int-AlHBH-1.log

SCF (wB97x) = -2209.01489422

E(SCF)+ZPE(0 K)= -2207.928662

H(298 K)= -2207.866023

G(298 K)= -2208.024896

Lowest Frequency = 19.6183cm-1

Al	0.625546	7.741716	5.817197
H	0.119719	6.331863	6.418206
N	-1.014411	8.634122	5.263456
C	-1.488096	9.749350	5.820684
C	-0.877632	10.403490	6.899531
H	-1.375251	11.300271	7.251902
C	0.207531	9.957221	7.674804
N	0.964875	8.920780	7.337860
C	-2.794072	10.337557	5.340996
H	-2.685861	11.417892	5.210049
H	-3.572412	10.176471	6.093126
H	-3.130265	9.895461	4.402857
C	0.427671	10.671832	8.985932
H	0.351687	11.754219	8.853389
H	1.388211	10.428554	9.440441
H	-0.363104	10.375236	9.683315
C	-1.844604	7.828250	4.422954
C	-1.579767	7.770470	3.035680
C	-2.363867	6.920687	2.249172
H	-2.183243	6.859478	1.181343
C	-3.374461	6.145085	2.810097
H	-3.968078	5.488303	2.180255
C	-3.610742	6.202005	4.177669
H	-4.389234	5.581272	4.613131
C	-2.854287	7.033144	5.010159
C	-0.494052	8.636223	2.414894
H	0.352928	8.649239	3.113015
C	0.020481	8.100341	1.078370
H	0.302069	7.045672	1.143155
H	0.903371	8.668084	0.769689
H	-0.725466	8.206766	0.282248
C	-0.965930	10.087257	2.248315
H	-1.874862	10.129556	1.637102
H	-0.191828	10.683314	1.754004
H	-1.168143	10.557387	3.211256
C	-3.139725	7.042423	6.504500
H	-2.492454	7.789728	6.973075
C	-2.799071	5.691612	7.147021
H	-3.001946	5.721447	8.223727
H	-1.741395	5.459382	7.002451
H	-3.399354	4.883160	6.714643
C	2.043052	8.467152	8.164684
C	1.801089	7.470753	9.134002
C	2.885495	6.993421	9.878329
H	2.715584	6.232177	10.635073
C	4.170523	7.478761	9.671340
H	4.999688	7.097130	10.261155
C	4.393512	8.455103	8.705038
H	5.401448	8.827001	8.548609
C	3.347503	8.962961	7.929131
C	0.407506	6.931995	9.415364
H	-0.274324	7.354235	8.670305
C	-0.074746	7.369079	10.806044
H	0.546068	6.927269	11.593718
H	-1.107907	7.045472	10.973803
H	-0.032409	8.455648	10.923541
C	0.344357	5.406732	9.274283

H	0.658276	5.100937	8.272794
H	-0.681003	5.055416	9.430261
H	0.982328	4.905625	10.010761
C	3.613376	10.038138	6.888720
H	2.831943	9.957600	6.128738
C	4.967354	9.877657	6.188939
H	5.803169	10.144413	6.845959
H	5.013243	10.548412	5.324554
H	5.125799	8.853683	5.838272
C	3.522747	11.447826	7.491702
H	3.750155	12.198669	6.727433
H	4.239833	11.565922	8.312610
N	1.852248	10.851858	4.003854
C	2.571980	10.490012	3.170789
H	3.355038	8.133772	4.785634
B	2.359580	7.492742	4.607473
C	3.450464	10.086050	2.116003
C	3.149924	10.462938	0.796594
H	2.248954	11.032182	0.591470
C	4.015480	10.105215	-0.230749
H	3.784918	10.391723	-1.252242
C	5.178364	9.385453	0.052001
H	5.852520	9.110290	-0.753969
C	5.471939	9.008923	1.362171
H	6.357444	8.421731	1.580019
C	4.609506	9.349266	2.398731
H	4.811931	9.032129	3.414155
N	2.551022	6.387988	3.762267
C	3.869632	5.984887	3.369968
C	4.245208	5.989018	2.014203
C	5.542851	5.597674	1.672620
H	5.833633	5.617862	0.623752
C	6.476855	5.210868	2.633332
C	6.081677	5.226811	3.973178
H	6.795586	4.930027	4.739461
C	4.794432	5.602785	4.365627
C	3.306077	6.466325	0.942402
H	2.626358	5.675821	0.603583
H	3.862130	6.818991	0.070824
H	2.695149	7.292684	1.310957
C	7.860744	4.765034	2.240130
H	7.910407	3.672931	2.149397
H	8.605245	5.061894	2.985503
H	8.159756	5.185543	1.275134
C	4.438407	5.601726	5.830560
H	4.472461	6.608701	6.260734
H	5.135854	4.977451	6.396247
H	3.425004	5.231531	6.004289
C	1.465846	5.467207	3.415540
H	1.544538	5.153639	2.370211
H	0.528055	6.020039	3.510946
C	1.453309	4.218710	4.294792
H	1.356599	4.527550	5.352503
H	2.420009	3.712500	4.184773
N	0.399245	3.291089	3.904219
C	0.629720	1.971343	4.467420
H	1.596630	1.584149	4.130419
H	0.622971	1.967528	5.575149
C	-0.921072	3.780783	4.278852
H	-1.117744	4.761856	3.842281
H	-1.043659	3.872134	5.373087
H	-1.686730	3.095595	3.901259
H	2.526237	11.664267	7.877580
H	-0.148693	1.282047	4.123709
C	-4.596779	7.428144	6.796807
H	-5.290105	6.653787	6.450386
H	-4.750405	7.548002	7.874607
H	-4.876646	8.363688	6.303864

Int-CNXyl-2.log

SCF (wB97x) = -2287.64296757

E(SCF)+ZPE(0 K)= -2286.499345

H(298 K)= -2286.434705

G(298 K)= -2286.596631

Lowest Frequency = 14.9783cm-1

Al	0.970592	8.158850	5.337942
H	0.916309	6.581213	5.558745
N	-0.840191	8.778672	5.202736
C	-1.326308	9.792719	5.930834
C	-0.639172	10.370718	7.002547
H	-1.145141	11.182049	7.511592
C	0.556627	9.925781	7.601385
N	1.371393	9.031313	7.047144
C	-2.683912	10.346984	5.586335
H	-2.636609	10.824005	4.601845
H	-3.011712	11.085656	6.318556

H	-3.434628	9.556333	5.514885	H	0.581196	8.070511	10.813752
C	0.841559	10.472081	8.978223	C	0.805531	5.414691	8.568485
H	0.590733	11.534810	9.023220	H	0.919748	5.315380	7.486730
H	1.878122	10.332101	9.283153	H	-0.156957	4.981056	8.855583
H	0.201817	9.955798	9.701357	H	1.591288	4.826352	9.053726
C	-1.752940	8.016648	4.392983	C	4.036645	10.186251	6.638437
C	-1.946826	8.333603	3.036681	H	3.405562	10.012202	5.764257
C	-2.894600	7.602055	2.311355	C	5.477528	10.260305	6.126627
H	-3.057178	7.839467	1.263587	H	6.168570	10.612417	6.901415
C	-3.630922	6.587658	2.906640	H	5.528860	10.971038	5.296254
H	-4.374384	6.041633	2.332491	H	5.829998	9.292957	5.760230
C	-3.396037	6.257339	4.237559	C	3.638766	11.542875	7.238308
H	-3.956284	5.444272	4.688194	H	3.909180	12.350180	6.549688
C	-2.444518	6.941283	4.998223	H	4.160396	11.715244	8.186401
C	-1.145495	9.408551	2.331460	H	4.578113	7.660754	5.119813
H	-0.502930	9.897240	3.069391	B	3.644156	7.420601	4.417779
C	-0.235367	8.774803	1.272384	C	2.399829	10.347496	2.488047
H	0.534713	8.153548	1.740364	C	1.662935	11.421586	3.032094
H	0.272192	9.549111	0.694484	C	1.205029	12.426831	2.167973
H	-0.808137	8.146877	0.581373	H	0.638231	13.252874	2.593321
C	-2.029282	10.489489	1.697131	C	1.456607	12.394622	0.803590
H	-2.616233	10.089799	0.862706	H	1.086269	13.186167	0.158055
H	-1.398412	11.295613	1.309601	C	2.204134	11.341826	0.273805
H	-2.732606	10.918074	2.418167	H	2.415133	11.306029	-0.792306
C	-2.174513	6.520368	6.434323	C	2.687159	10.327857	1.094434
H	-1.178270	6.888199	6.698383	N	3.569484	6.143483	3.855380
C	-2.144469	4.996325	6.585240	C	4.621691	5.196953	4.080343
H	-1.868780	4.724757	7.607185	C	5.534113	4.904036	3.052034
H	-1.412643	4.550175	5.904594	C	6.542734	3.967929	3.300138
H	-3.120275	4.541406	6.386430	H	7.254492	3.743496	2.507574
C	2.480941	8.512605	7.806199	C	6.671320	3.329426	4.533968
C	2.251821	7.470313	8.728254	C	5.768060	3.664902	5.545827
C	3.342031	6.962049	9.442412	H	5.863203	3.192412	6.521844
H	3.181659	6.155192	10.152053	C	4.743484	4.591989	5.345515
C	4.621414	7.465852	9.256800	C	5.467393	5.598661	1.717149
H	5.457302	7.057146	9.817594	H	4.833318	5.052561	1.008132
C	4.831017	8.495032	8.344535	H	6.463074	5.671030	1.270349
H	5.834884	8.881042	8.205537	H	5.058319	6.608316	1.815406
C	3.778885	9.039253	7.602569	C	7.741312	2.294860	4.765486
C	0.873654	6.886915	8.991640	H	7.360498	1.285271	4.566872
H	0.143502	7.437810	8.390697	H	8.096464	2.309552	5.800631
C	0.472841	7.039092	10.466484	H	8.602237	2.453253	4.109043
H	1.091452	6.407983	11.113619	C	3.819198	4.961981	6.472043
H	-0.569816	6.735820	10.609293	H	4.071540	5.944861	6.885090

H	3.878446	4.236149	7.287470
H	2.781951	5.031612	6.138268
C	2.473094	5.711652	2.985932
H	2.841749	5.485940	1.978649
H	1.791407	6.559151	2.889332
C	1.738507	4.486321	3.524110
H	1.308842	4.732392	4.512431
H	2.469141	3.682876	3.673618
N	0.715573	4.003010	2.603528
C	0.216677	2.709365	3.040644
H	1.041038	1.991441	3.098683
H	-0.274140	2.749889	4.033278
C	-0.386142	4.947013	2.460409
H	-0.045860	5.883016	2.013732
H	-0.875167	5.189453	3.420695
H	-1.143368	4.527211	1.791903
H	2.565361	11.616171	7.418647
H	-0.513450	2.330040	2.318391
C	-3.174503	7.146626	7.415066
H	-4.200793	6.854168	7.165642
H	-2.964949	6.812952	8.437545
H	-3.119847	8.238477	7.405273
C	3.464776	9.178538	0.517567
H	4.412812	9.046919	1.049048
H	2.916805	8.235696	0.632304
H	3.663492	9.330516	-0.547237
C	1.359351	11.582718	4.495905
H	1.796169	10.801752	5.112309
H	1.736600	12.542430	4.866282
H	0.280761	11.572885	4.677785
N	3.008548	9.303137	3.191669
C	2.542099	8.515822	4.101533

Int-CNXyl-3.log

SCF (wB97x) = -2287.62901332
E(SCF)+ZPE(0 K)= -2286.485323
H(298 K)= -2286.420957
G(298 K)= -2286.581512
Lowest Frequency = 22.9235cm-1

Al	0.809609	8.096975	5.536181
H	0.652953	6.528332	5.673530
N	-0.948495	8.851154	5.321218
C	-1.403240	9.851308	6.081038
C	-0.677950	10.400362	7.149133
H	-1.163564	11.214960	7.673855
C	0.481469	9.886220	7.758635
N	1.246821	8.939488	7.213645
C	-2.796497	10.387790	5.870498
H	-3.213551	10.096407	4.906608
H	-2.792622	11.478078	5.951192
H	-3.458875	10.005383	6.653355
C	0.789470	10.393406	9.145621
H	0.414956	11.410166	9.278324
H	1.853856	10.365986	9.377930
H	0.280931	9.752147	9.873547
C	-1.858150	8.099910	4.499485
C	-1.832362	8.260530	3.097792
C	-2.715870	7.498022	2.329171
H	-2.717833	7.611121	1.250216
C	-3.584831	6.587034	2.919689
H	-4.259308	5.999813	2.302803
C	-3.575062	6.418710	4.298326
H	-4.238206	5.688245	4.753431
C	-2.715973	7.162506	5.113005
C	-0.906954	9.268146	2.441197
H	-0.010610	9.341064	3.065283
C	-0.438004	8.864655	1.042436
H	0.021413	7.871213	1.041270
H	0.307938	9.580830	0.688691
H	-1.258388	8.861339	0.316746
C	-1.559769	10.656394	2.402290
H	-2.498299	10.621385	1.837745
H	-0.891021	11.374817	1.919282
H	-1.783265	11.025739	3.406323
C	-2.703110	6.894849	6.608776
H	-2.020251	7.606696	7.083326
C	-2.162383	5.487396	6.891375
H	-2.107403	5.310127	7.970918
H	-1.162240	5.364647	6.467838
H	-2.814511	4.721189	6.458211
C	2.353033	8.407441	7.967644
C	2.147798	7.291569	8.801375
C	3.219955	6.842759	9.581592
H	3.076585	5.989544	10.238882
C	4.458464	7.465222	9.529642
H	5.275935	7.109260	10.150376

C	4.660076	8.527408	8.653509
H	5.644101	8.978853	8.587134
C	3.628282	9.007037	7.843059
C	0.828909	6.538313	8.863958
H	0.132792	7.004723	8.159259
C	0.192461	6.605626	10.257875
H	0.819938	6.109883	11.006527
H	-0.781643	6.104546	10.256074
H	0.041857	7.638953	10.584270
C	1.027537	5.081677	8.422440
H	1.399068	5.035756	7.395458
H	0.083028	4.532283	8.469999
H	1.744381	4.564002	9.068327
C	3.894721	10.113358	6.834352
H	3.290870	9.866560	5.955056
C	5.351866	10.163383	6.365295
H	6.012268	10.584538	7.132252
H	5.427902	10.804989	5.481826
H	5.721169	9.172505	6.090475
C	3.467549	11.504443	7.325556
H	3.779686	12.268178	6.605156
H	3.938873	11.735998	8.287211
H	4.686888	8.042096	4.460659
B	3.663982	7.724719	3.928040
C	2.548050	10.027998	2.370734
C	1.900936	11.210360	2.790995
C	1.692969	12.213003	1.837396
H	1.201827	13.131789	2.148882
C	2.095461	12.058890	0.515072
H	1.913125	12.850529	-0.206003
C	2.749939	10.892245	0.125926
H	3.079285	10.769004	-0.902504
C	3.001239	9.873355	1.043410
N	3.672564	6.398088	3.281413
C	4.719603	5.495445	3.610088
C	5.499194	4.895037	2.596492
C	6.523161	4.010964	2.946801
H	7.122772	3.565690	2.153892
C	6.810681	3.697109	4.275031
C	6.027992	4.292679	5.264687
H	6.221360	4.052213	6.309297
C	4.983386	5.170921	4.963423
C	5.261526	5.200337	1.142609
H	4.493893	4.544714	0.712467

H	6.175093	5.057596	0.557763
H	4.919046	6.228557	1.014217
C	7.901456	2.719229	4.626676
H	7.513766	1.693709	4.676652
H	8.345309	2.945682	5.601282
H	8.702638	2.726338	3.880811
C	4.156174	5.726223	6.094076
H	4.515203	6.704732	6.429209
H	4.179295	5.058451	6.959983
H	3.114155	5.867630	5.799458
C	2.481331	5.827705	2.665651
H	2.687436	5.443102	1.656330
H	1.758497	6.640825	2.540707
C	1.857958	4.690861	3.481015
H	1.595344	5.068133	4.484401
H	2.622012	3.916627	3.616290
N	0.705151	4.074655	2.830289
C	0.327464	2.853684	3.522660
H	1.169882	2.154588	3.532990
H	0.016546	3.031993	4.571274
C	-0.432488	4.979943	2.754087
H	-0.189497	5.860644	2.157037
H	-0.777139	5.331197	3.743894
H	-1.271562	4.479224	2.261868
H	2.385777	11.592452	7.442502
H	-0.506546	2.372533	3.000445
C	-4.086917	7.082427	7.244196
H	-4.795750	6.328424	6.885508
H	-4.022194	6.980995	8.332649
H	-4.509570	8.065295	7.016064
C	3.716193	8.619942	0.631272
H	4.573800	8.430228	1.282835
H	3.065453	7.744769	0.721539
H	4.062572	8.686604	-0.403075
C	1.443153	11.442181	4.204462
H	0.497450	10.936345	4.418154
H	2.166532	11.075569	4.933796
H	1.290306	12.509144	4.386211
N	2.860867	8.990239	3.268290
C	2.305369	8.452879	4.302054

Int-CO2-1.log

SCF (wB97x) = -2073.16725443

E(SCF)+ZPE(0 K)= -2072.169258
H(298 K)= -2072.110364
G(298 K)= -2072.263650
Lowest Frequency = 5.6186cm-1

Al	0.554768	7.622030	6.018711
H	-0.566744	6.591749	6.924447
N	-0.631218	8.692628	4.986191
C	-1.148825	9.839718	5.429045
C	-0.773457	10.424043	6.651679
H	-1.335809	11.300053	6.951198
C	0.279353	10.043886	7.487720
N	1.054110	8.975308	7.240885
C	-2.194475	10.552223	4.616219
H	-2.103814	11.634276	4.729318
H	-3.185941	10.260714	4.979517
H	-2.136826	10.290457	3.558329
C	0.549785	10.856341	8.723135
H	0.017370	11.807917	8.706261
H	1.617102	11.039637	8.863965
H	0.208243	10.281397	9.592084
C	-0.950629	8.138489	3.700678
C	0.014857	8.242733	2.671750
C	-0.257274	7.619152	1.452173
H	0.463781	7.684903	0.644407
C	-1.435468	6.903298	1.255611
H	-1.623465	6.420176	0.300848
C	-2.365321	6.803873	2.282048
H	-3.278720	6.236710	2.125702
C	-2.145603	7.414532	3.522301
C	1.290744	9.045388	2.872053
H	1.584779	8.941583	3.923395
C	2.472224	8.544121	2.040285
H	2.659672	7.480951	2.215047
H	3.376754	9.091976	2.316770
H	2.316057	8.698076	0.967212
C	1.036655	10.539354	2.629944
H	0.718768	10.713521	1.596089
H	1.949277	11.117750	2.809858
H	0.258537	10.926290	3.293247
C	-3.170503	7.252126	4.630434
H	-2.848774	7.842133	5.493031
C	-3.234960	5.792497	5.094517
H	-3.917885	5.686854	5.942999
H	-2.253946	5.440383	5.419192
H	-3.580054	5.132626	4.290657
C	2.341780	8.850669	7.864789
C	2.500006	8.057221	9.014625
C	3.783051	7.924969	9.555817
H	3.926208	7.309838	10.439837
C	4.873773	8.565847	8.980122
H	5.862215	8.458770	9.418437
C	4.699912	9.340273	7.836414
H	5.558882	9.833273	7.390667
C	3.441126	9.489023	7.248666
C	1.330485	7.314150	9.630338
H	0.416218	7.672371	9.146028
C	1.181660	7.584537	11.131323
H	2.030004	7.189336	11.700623
H	0.275464	7.102237	11.510985
H	1.110965	8.657286	11.337867
C	1.462523	5.811387	9.346899
H	1.554723	5.622990	8.271982
H	0.584173	5.266468	9.701992
H	2.356300	5.402962	9.832029
C	3.273484	10.335105	5.996461
H	2.303824	10.082856	5.554736
C	4.348062	10.048865	4.942892
H	5.342378	10.363210	5.277055
H	4.127142	10.604874	4.025922
H	4.390616	8.984252	4.697570
C	3.240478	11.832283	6.335684
H	3.119166	12.428024	5.424464
H	4.173575	12.139259	6.821305
H	3.151182	7.121024	4.941804
B	2.102193	6.554565	5.072722
N	2.042996	5.258130	4.572726
C	3.216189	4.656801	4.001577
C	3.247282	4.370277	2.624715
C	4.402767	3.795101	2.089756
H	4.433582	3.581217	1.022856
C	5.514880	3.497679	2.878148
C	5.459349	3.811696	4.237742
H	6.322537	3.601317	4.866290
C	4.331612	4.394301	4.821019
C	2.086005	4.683575	1.716160
H	1.455977	3.800277	1.554476
H	2.444845	5.005881	0.734113

H	1.447144	5.473118	2.119074
C	6.731600	2.836965	2.285855
H	6.847885	3.085498	1.226667
H	6.657537	1.744927	2.358762
H	7.645483	3.136449	2.807832
C	4.358240	4.764741	6.281841
H	4.598966	5.825805	6.414822
H	5.115194	4.182384	6.814433
H	3.394291	4.603262	6.770405
C	0.869326	4.381956	4.668946
H	0.792471	3.763575	3.769762
H	-0.020598	5.016928	4.707892
C	0.963500	3.464938	5.887258
H	0.805028	4.075239	6.798171
H	1.983876	3.066228	5.928808
N	0.055878	2.329404	5.826471
C	0.291754	1.437495	6.951731
H	1.334209	1.102062	6.945762
H	0.086797	1.912401	7.929624
C	-1.342844	2.737118	5.772751
H	-1.545612	3.271812	4.840622
H	-1.633141	3.383811	6.618595
H	-1.978045	1.845934	5.778321
H	2.414210	12.077307	7.007880
H	-0.347817	0.553719	6.861544
C	-4.558595	7.746901	4.202971
H	-4.983222	7.115564	3.414805
H	-5.246577	7.719600	5.053992
H	-4.524764	8.771800	3.821373
O	-2.086121	7.605767	7.944689
O	-1.613897	5.378123	8.192247
C	-1.559783	6.522841	7.809430

PhCN.log

SCF (wB97x) = -324.394838003

E(SCF)+ZPE(0 K)= -324.295264

H(298 K)= -324.288203

G(298 K)= -324.325558

Lowest Frequency = 144.0032cm-1

C	-1.582604	0.571119	0.000055
C	-0.191844	0.563037	0.000608
C	0.503882	1.781734	0.000102
C	-0.191742	3.000498	-0.000951
C	-1.582496	2.992533	-0.001497
C	-2.276987	1.781853	-0.000996
H	-2.125313	-0.369072	0.000443
H	0.358474	-0.372149	0.001421
H	0.358665	3.935632	-0.001331
H	-2.125135	3.932766	-0.002314
H	-3.363031	1.781905	-0.001426
C	1.935590	1.781686	0.000663
N	3.094851	1.781648	0.001116

TS1.log

SCF (wB97x) = -2073.16597428

E(SCF)+ZPE(0 K)= -2072.166819

H(298 K)= -2072.108814

G(298 K)= -2072.258657

Lowest Frequency = -155.9523cm-1

Al	0.515124	7.627647	6.022269
H	-0.466389	6.260701	6.862521
N	-0.660283	8.686939	4.990164
C	-1.157307	9.842830	5.438444
C	-0.761237	10.422818	6.655974
H	-1.308839	11.307382	6.958244
C	0.295212	10.034966	7.484756
N	1.058207	8.959304	7.231915
C	-2.203003	10.565566	4.635709
H	-2.115506	11.646059	4.763936
H	-3.193874	10.265194	4.993417
H	-2.144159	10.318862	3.574150
C	0.579024	10.842676	8.719371
H	0.083200	11.813687	8.692084
H	1.650191	10.985024	8.876645
H	0.200355	10.283214	9.583225
C	-0.983965	8.146327	3.698387
C	-0.017816	8.253027	2.670253
C	-0.295460	7.643890	1.444459
H	0.426453	7.710967	0.637588
C	-1.481131	6.943090	1.239756
H	-1.673994	6.472363	0.279772
C	-2.412304	6.842763	2.264816
H	-3.331620	6.287046	2.102371
C	-2.185831	7.436174	3.512233

C	1.264294	9.044535	2.874704	H	3.096700	7.143965	4.954490
H	1.557206	8.934316	3.925421	B	2.058611	6.556560	5.081104
C	2.442292	8.537551	2.041545	N	2.025609	5.258788	4.585346
H	2.623440	7.473110	2.214629	C	3.215829	4.679134	4.027830
H	3.350249	9.079283	2.318289	C	3.266301	4.388339	2.652753
H	2.286749	8.694120	0.968809	C	4.439599	3.836382	2.131818
C	1.021816	10.541378	2.638631	H	4.485939	3.618655	1.066287
H	0.706485	10.722265	1.605157	C	5.550033	3.567313	2.932535
H	1.938535	11.112073	2.822221	C	5.474436	3.885897	4.290370
H	0.245793	10.931347	3.302595	H	6.336028	3.697469	4.927971
C	-3.209654	7.265695	4.619992	C	4.328417	4.445384	4.860036
H	-2.876538	7.834432	5.492598	C	2.107478	4.673334	1.731462
C	-3.288629	5.797053	5.053696	H	1.494482	3.777732	1.572082
H	-3.956263	5.682239	5.912619	H	2.469361	4.993228	0.749802
H	-2.307874	5.421992	5.352513	H	1.451221	5.455238	2.121802
H	-3.657284	5.160642	4.241357	C	6.787287	2.931516	2.355721
C	2.351824	8.814919	7.841421	H	6.900017	3.164064	1.292582
C	2.511078	8.017764	8.988515	H	6.744651	1.839329	2.448603
C	3.799446	7.858235	9.509699	H	7.690333	3.266718	2.874914
H	3.942870	7.239132	10.390891	C	4.329561	4.821102	6.319956
C	4.894087	8.477618	8.918748	H	4.520684	5.892203	6.453814
H	5.886838	8.349078	9.341254	H	5.108346	4.274455	6.858768
C	4.718312	9.259334	7.780258	H	3.370822	4.616439	6.802752
H	5.580227	9.736343	7.323347	C	0.866379	4.363490	4.674455
C	3.454238	9.434539	7.211486	H	0.809179	3.738091	3.778742
C	1.335986	7.303554	9.626859	H	-0.034357	4.983566	4.699024
H	0.421824	7.668042	9.148054	C	0.963033	3.456992	5.899835
C	1.210242	7.600848	11.125058	H	0.812293	4.076612	6.806547
H	2.060398	7.203778	11.690478	H	1.982633	3.055873	5.941120
H	0.302033	7.138205	11.523850	N	0.049421	2.326608	5.855102
H	1.157028	8.677883	11.313791	C	0.284818	1.446627	6.990081
C	1.439264	5.793900	9.367947	H	1.325381	1.105355	6.983998
H	1.532218	5.585881	8.296576	H	0.086156	1.934461	7.962817
H	0.549471	5.272322	9.730351	C	-1.347201	2.744047	5.803734
H	2.322922	5.373770	9.861522	H	-1.553908	3.258565	4.861045
C	3.284400	10.288756	5.965441	H	-1.625669	3.411248	6.637752
H	2.316549	10.034235	5.521072	H	-1.988910	1.858163	5.834187
C	4.361547	10.018605	4.910569	H	2.413425	12.018697	6.988332
H	5.352372	10.343290	5.245171	H	-0.359999	0.565529	6.912862
H	4.134130	10.576320	3.996333	C	-4.593625	7.782587	4.205701
H	4.416435	8.955579	4.660527	H	-5.025496	7.172301	3.404956
C	3.242168	11.783130	6.316064	H	-5.280464	7.743388	5.057180
H	3.119469	12.384993	5.409078	H	-4.551398	8.815035	3.845912
H	4.172767	12.090914	6.805930	O	-1.719358	7.644173	7.722421

O	-1.736280	5.406602	8.173157
C	-1.398516	6.456957	7.662638

TS2.log

SCF (wB97x) = -2287.61119460
E(SCF)+ZPE(0 K)= -2286.469615
H(298 K)= -2286.405019
G(298 K)= -2286.565522
Lowest Frequency = -290.9019cm-1

Al	1.098212	7.832154	5.540288
H	0.871853	6.317653	5.957187
N	-0.685806	8.543710	5.281757
C	-1.189317	9.582331	5.959558
C	-0.501356	10.251882	6.977792
H	-1.015103	11.091391	7.430497
C	0.719148	9.891656	7.575289
N	1.513254	8.931322	7.106711
C	-2.583159	10.055632	5.639633
H	-2.634431	10.392786	4.600789
H	-2.882830	10.876018	6.292393
H	-3.306304	9.241546	5.736993
C	1.065825	10.622409	8.848339
H	0.947507	11.699983	8.702993
H	2.078016	10.412822	9.192264
H	0.367263	10.324456	9.636565
C	-1.588012	7.768870	4.470252
C	-1.781626	8.091212	3.112892
C	-2.736196	7.371113	2.386470
H	-2.903417	7.618890	1.341843
C	-3.470802	6.349720	2.973553
H	-4.214488	5.807944	2.395908
C	-3.232449	6.010820	4.300582
H	-3.792598	5.196764	4.752083
C	-2.287535	6.696839	5.069052
C	-0.973490	9.161404	2.406624
H	-0.298602	9.617268	3.137290
C	-0.116139	8.523405	1.307788
H	0.614608	7.830315	1.734365
H	0.418234	9.296005	0.753438
H	-0.733044	7.964515	0.597075
C	-1.841934	10.277273	1.812499
H	-2.480286	9.899383	1.006123
H	-1.199942	11.060968	1.398939
H	-2.494735	10.735105	2.561969
C	-2.057226	6.263067	6.507470
H	-1.215002	6.836572	6.904807
C	-1.666425	4.781296	6.565039
H	-1.466343	4.477419	7.596229
H	-0.763380	4.602832	5.974254
H	-2.462844	4.136394	6.178959
C	2.625420	8.469374	7.892372
C	2.392515	7.529713	8.917424
C	3.496211	7.024886	9.613455
H	3.337234	6.294587	10.402116
C	4.787998	7.435071	9.310288
H	5.632815	7.026064	9.857374
C	4.997845	8.374119	8.305084
H	6.009590	8.693823	8.076922
C	3.930918	8.907409	7.578068
C	0.998031	7.063935	9.303142
H	0.273897	7.575217	8.661031
C	0.676339	7.434226	10.758400
H	1.307454	6.874510	11.457248
H	-0.367123	7.196262	10.991162
H	0.837340	8.498918	10.950313
C	0.821008	5.558660	9.076487
H	0.950427	5.308753	8.021469
H	-0.182415	5.246878	9.385454
H	1.543675	4.979145	9.660909
C	4.177982	9.957250	6.509088
H	3.345824	9.900339	5.802966
C	5.468607	9.737017	5.714068
H	6.358772	9.969836	6.309137
H	5.476184	10.397271	4.841186
H	5.549431	8.706871	5.357760
C	4.167623	11.367371	7.117280
H	4.392610	12.116923	6.350400
H	4.925998	11.452855	7.903569
H	3.631225	7.492685	5.484176
B	3.185432	7.304175	4.384281
C	2.246161	10.578955	2.370526
C	1.558301	11.584788	3.079779
C	1.021230	12.655090	2.359069
H	0.481562	13.427701	2.901712
C	1.153112	12.742903	0.976524
H	0.717414	13.579212	0.437612

C	1.857468	11.754405	0.290659
H	1.977399	11.820085	-0.787804
C	2.427934	10.679089	0.968440
N	3.220536	5.949308	3.861918
C	4.336563	5.153833	4.256148
C	5.440878	5.045818	3.380593
C	6.535062	4.263712	3.754626
H	7.380785	4.182429	3.073410
C	6.586664	3.610350	4.987907
C	5.507876	3.772529	5.857367
H	5.534765	3.289462	6.832793
C	4.383418	4.531993	5.518928
C	5.466309	5.800654	2.076975
H	4.899007	5.288824	1.290216
H	6.491824	5.917898	1.715891
H	5.020985	6.794088	2.194962
C	7.762910	2.745493	5.358650
H	7.623729	1.716767	5.003048
H	7.901488	2.698654	6.443203
H	8.691203	3.118560	4.914237
C	3.272905	4.705136	6.512118
H	3.343735	5.676088	7.016483
H	3.305158	3.930530	7.283670
H	2.295310	4.690791	6.026474
C	2.435861	5.498262	2.721480
H	2.945415	4.662953	2.230524
H	2.347047	6.302883	1.978473
C	1.057629	4.989452	3.165608
H	0.413773	5.837137	3.475479
H	1.216738	4.381254	4.061234
N	0.394573	4.154733	2.171929
C	-0.728295	3.446687	2.768709
H	-0.375701	2.824507	3.597597
H	-1.508099	4.128101	3.155534
C	-0.045812	4.911880	1.010720
H	0.798585	5.428527	0.546338
H	-0.813755	5.664009	1.264606
H	-0.466396	4.227710	0.266119
H	3.197625	11.614787	7.554779
H	-1.186505	2.788625	2.022472
C	-3.275115	6.554656	7.393170
H	-4.160351	6.009892	7.046686
H	-3.076114	6.248467	8.426061
H	-3.519254	7.621523	7.400250

C	3.204816	9.616651	0.244717
H	4.200708	9.499629	0.686292
H	2.715690	8.638601	0.325192
H	3.311868	9.858406	-0.815933
C	1.437137	11.546817	4.573480
H	1.286153	10.534481	4.943328
H	2.343426	11.932801	5.053191
H	0.593652	12.147680	4.919754
N	2.888470	9.503651	2.961432
C	2.744269	8.524698	3.696315

TS3.log

SCF (wB97x) = -2208.99588523

E(SCF)+ZPE(0 K)= -2207.910532

H(298 K)= -2207.848848

G(298 K)= -2208.004634

Lowest Frequency = -244.5933cm-1

Al	1.155583	7.801180	5.459128
H	0.854369	6.310195	5.908051
N	-0.557559	8.584407	5.034545
C	-1.005035	9.723683	5.574105
C	-0.339922	10.408041	6.600086
H	-0.817684	11.313427	6.954447
C	0.803364	10.004118	7.311867
N	1.563733	8.970426	6.951691
C	-2.281513	10.329676	5.058164
H	-2.063258	10.821944	4.103528
H	-2.675905	11.077086	5.747629
H	-3.045930	9.573854	4.868087
C	1.106449	10.766834	8.575599
H	0.924055	11.834563	8.431960
H	2.128007	10.615681	8.923373
H	0.427664	10.422450	9.363106
C	-1.471013	7.766680	4.277045
C	-1.628230	7.937995	2.888607
C	-2.544460	7.120345	2.218164
H	-2.674861	7.241608	1.146497
C	-3.290085	6.164039	2.893921
H	-4.000178	5.542741	2.355434
C	-3.119098	6.003121	4.264400
H	-3.708447	5.258357	4.790924
C	-2.210081	6.787392	4.979289

C	-0.842255	8.964285	2.099158
H	-0.266076	9.567607	2.805050
C	0.148242	8.263944	1.162461
H	0.840618	7.636083	1.727829
H	0.734118	8.998189	0.603946
H	-0.374768	7.626827	0.441727
C	-1.753253	9.911637	1.307904
H	-2.281759	9.386065	0.504891
H	-1.153582	10.705343	0.853695
H	-2.507228	10.378600	1.949022
C	-2.092946	6.606636	6.483483
H	-1.193108	7.130771	6.818176
C	-1.926680	5.135556	6.873267
H	-1.787186	5.045541	7.954129
H	-1.051987	4.703679	6.378184
H	-2.804457	4.537096	6.608119
C	2.668469	8.558287	7.777494
C	2.437005	7.680810	8.854167
C	3.540819	7.241469	9.594140
H	3.384949	6.558950	10.425073
C	4.827936	7.660042	9.284154
H	5.672104	7.305256	9.868828
C	5.035681	8.534117	8.220887
H	6.044981	8.854101	7.982967
C	3.970434	8.996056	7.445384
C	1.046237	7.212103	9.248456
H	0.321894	7.679484	8.573215
C	0.701323	7.648652	10.679929
H	1.329966	7.131900	11.413112
H	-0.341951	7.408660	10.911161
H	0.846814	8.723445	10.822315
C	0.901073	5.693355	9.097077
H	1.014267	5.393978	8.053035
H	-0.085705	5.369756	9.443218
H	1.651571	5.161646	9.691524
C	4.209706	9.948369	6.287388
H	3.411218	9.770486	5.565393
C	5.531161	9.705254	5.553912
H	6.393899	10.037987	6.141977
H	5.533919	10.268760	4.615974
H	5.664176	8.647464	5.310983
C	4.105014	11.416361	6.723008
H	4.293305	12.075685	5.869249
H	4.840915	11.644326	7.502274
N	2.874148	8.364760	3.573828
C	3.005922	9.392735	2.897531
H	3.745347	7.394464	5.350182
B	3.207251	7.198671	4.292858
C	2.120762	10.538355	2.781714
C	1.515255	11.096050	3.920875
H	1.675480	10.642058	4.891354
C	0.706389	12.223386	3.813586
H	0.240441	12.626434	4.708710
C	0.483557	12.812584	2.569320
H	-0.153119	13.688475	2.483913
C	1.104804	12.282046	1.433897
H	0.948686	12.745543	0.463620
C	1.937268	11.174197	1.540718
H	2.444810	10.777177	0.666280
N	3.246270	5.846184	3.781761
C	4.331286	5.027910	4.220541
C	5.436892	4.848752	3.357872
C	6.500415	4.043621	3.768979
H	7.346926	3.908539	3.097440
C	6.521692	3.433614	5.025088
C	5.444210	3.665396	5.879362
H	5.447937	3.218205	6.872067
C	4.349200	4.451373	5.505202
C	5.499722	5.549762	2.026010
H	4.928991	5.021284	1.252836
H	6.532674	5.623477	1.674949
H	5.081078	6.558844	2.096243
C	7.664776	2.542407	5.434762
H	7.500942	1.511144	5.097594
H	7.783198	2.514509	6.522291
H	8.611292	2.878411	4.999255
C	3.243725	4.696502	6.489924
H	3.358886	5.671847	6.978497
H	3.236566	3.935220	7.275294
H	2.267872	4.716481	6.001732
C	2.444316	5.357603	2.669150
H	2.958041	4.520136	2.186918
H	2.335124	6.145456	1.914375
C	1.080190	4.838416	3.142443
H	0.412959	5.685574	3.401354
H	1.250029	4.283510	4.070323
N	0.448192	3.931164	2.193233
C	-0.682912	3.252381	2.806984

H	-0.345931	2.694868	3.686964
H	-1.481566	3.947919	3.123212
C	0.039585	4.598159	0.967109
H	0.897640	5.075209	0.485790
H	-0.730777	5.369508	1.142448
H	-0.364962	3.859318	0.267235
H	3.113630	11.657601	7.113016
H	-1.111378	2.536534	2.097115
C	-3.289365	7.247401	7.199970
H	-4.228941	6.772731	6.895563
H	-3.190476	7.138995	8.285662
H	-3.363482	8.315275	6.971722

TS4.log

SCF (wB97x) = -2533.42326562

E(SCF)+ZPE(0 K)= -2532.234291

H(298 K)= -2532.166703

G(298 K)= -2532.335717

Lowest Frequency = -45.5917cm-1

Al	1.008149	8.022638	5.165767
H	0.568683	6.547960	5.541372
N	-0.660693	8.917506	4.814226
C	-0.976781	10.120779	5.302313
C	-0.242502	10.747058	6.317168
H	-0.625325	11.698372	6.664994
C	0.836060	10.214462	7.049788
N	1.532414	9.140618	6.670435
C	-2.188885	10.829139	4.762672
H	-1.984378	11.108595	3.722764
H	-2.416808	11.731902	5.330771
H	-3.066036	10.177858	4.754376
C	1.156604	10.883848	8.362803
H	0.721475	11.883112	8.411237
H	2.228199	10.945170	8.554780
H	0.722872	10.282768	9.169710
C	-1.711688	8.114795	4.247686
C	-1.927330	8.049117	2.860604
C	-2.972251	7.244127	2.390282
H	-3.148530	7.179676	1.320128
C	-3.785923	6.531773	3.261008
H	-4.595383	5.918578	2.874589
C	-3.553019	6.600276	4.630849
H	-4.186156	6.036919	5.310845
C	-2.513446	7.375510	5.148014
C	-1.073269	8.812478	1.869052
H	-0.386670	9.450231	2.432756
C	-0.234859	7.843014	1.025694
H	0.497706	7.315067	1.642226
H	0.316302	8.386843	0.254014
H	-0.868460	7.097546	0.532100
C	-1.918507	9.724078	0.970233
H	-2.564408	9.146702	0.299168
H	-1.261827	10.346482	0.355410
H	-2.560249	10.386230	1.560359
C	-2.288513	7.421159	6.650418
H	-1.344502	7.942861	6.830700
C	-2.148332	6.019127	7.249978
H	-1.913366	6.087804	8.317507
H	-1.341041	5.471481	6.756175
H	-3.071215	5.437324	7.153937
C	2.648826	8.735527	7.486210
C	2.458245	7.777903	8.497740
C	3.544207	7.453390	9.319191
H	3.412324	6.716761	10.107039
C	4.781799	8.054506	9.142269
H	5.611234	7.803668	9.797940
C	4.964948	8.963221	8.102919
H	5.946734	9.400325	7.954407
C	3.920386	9.308378	7.241904
C	1.141107	7.048547	8.694463
H	0.417353	7.454177	7.981677
C	0.567143	7.249300	10.101941
H	1.219946	6.816619	10.867808
H	-0.409429	6.760954	10.188032
H	0.437209	8.310223	10.337719
C	1.315680	5.557610	8.372872
H	1.671986	5.426474	7.347592
H	0.363989	5.027600	8.475932
H	2.037410	5.089930	9.051614
C	4.171378	10.231524	6.055523
H	3.600568	9.812693	5.221728
C	5.639864	10.262733	5.617856
H	6.257654	10.854287	6.303501
H	5.712393	10.730145	4.631013
H	6.072052	9.261521	5.555694
C	3.702872	11.678620	6.274287

H	3.989089	12.293406	5.414650
H	4.169042	12.105687	7.169596
N	2.439677	7.984467	3.895821
C	2.902034	8.692409	2.893842
C	5.735735	7.412646	3.670516
N	4.777264	7.169119	4.258651
H	3.071143	6.718287	5.647726
B	3.273314	6.780882	4.439033
C	2.160005	9.906726	2.523987
C	1.639480	10.810132	3.469199
H	1.752852	10.603044	4.526855
C	0.984848	11.974278	3.074504
H	0.590187	12.646393	3.832679
C	0.822057	12.261498	1.719409
H	0.301863	13.162877	1.407088
C	1.359124	11.390777	0.764744
H	1.253070	11.615623	-0.293652
C	2.046411	10.249841	1.163312
H	2.494259	9.586867	0.427494
C	6.964336	7.592439	2.991371
C	7.280735	8.831182	2.413216
H	6.578914	9.654232	2.490192
C	8.486848	8.962892	1.736226
H	8.747626	9.913154	1.281420
C	9.355801	7.873604	1.634844
H	10.295255	7.984713	1.101175
C	9.026584	6.641742	2.204993
H	9.702837	5.797913	2.111861
C	7.827405	6.487633	2.889281
H	7.537870	5.534823	3.321711
N	3.127042	5.506770	3.639036
C	4.231142	4.674038	3.364620
C	4.744372	4.601813	2.046716
C	5.863667	3.806562	1.790826
H	6.257896	3.766706	0.776382
C	6.520444	3.102131	2.803597
C	6.030727	3.223641	4.106371
H	6.535259	2.696437	4.914863
C	4.899158	3.990113	4.404346
C	4.149968	5.443694	0.950617
H	3.298616	4.949502	0.466492
H	4.892576	5.648273	0.173353
H	3.784798	6.394404	1.358972
C	7.715907	2.238597	2.495431

H	7.408287	1.236526	2.171476
H	8.359475	2.113266	3.371796
H	8.320521	2.665250	1.688005
C	4.415029	4.105097	5.824202
H	4.702232	5.065523	6.268839
H	4.827256	3.307359	6.449150
H	3.323676	4.068442	5.869004
C	1.835962	5.083905	3.139046
H	1.900367	4.727521	2.101988
H	1.173646	5.952995	3.129180
C	1.224975	3.963339	3.984971
H	1.064813	4.342798	5.013567
H	1.963348	3.153887	4.045374
N	0.000834	3.410927	3.417048
C	-0.381290	2.191076	4.106051
H	0.426847	1.455257	4.040010
H	-0.609860	2.354705	5.178513
C	-1.095495	4.367437	3.436931
H	-0.849771	5.263345	2.863538
H	-1.364493	4.692452	4.457767
H	-1.983380	3.921451	2.978059
H	2.620191	11.764132	6.377323
H	-1.271125	1.759976	3.634421
C	-3.392110	8.222806	7.352493
H	-4.373288	7.760451	7.196289
H	-3.207389	8.267835	8.431479
H	-3.440309	9.248708	6.974664

TS5.log

SCF (wB97x) = -2533.51038504

E(SCF)+ZPE(0 K)= -2532.320189

H(298 K)= -2532.253264

G(298 K)= -2532.420416

Lowest Frequency = -1009.0136cm-1

Al	1.191933	8.000341	5.139046
H	0.987623	6.480701	5.493039
N	-0.497607	8.801548	4.744839
C	-0.943849	9.925617	5.317572
C	-0.322549	10.519802	6.422998
H	-0.792710	11.415130	6.811021
C	0.779653	10.037372	7.153960
N	1.555509	9.034280	6.736030

C	-2.156141	10.603529	4.741921	C	0.328867	7.308793	10.157706
H	-1.839871	11.130119	3.832973	H	0.898506	6.805844	10.946596
H	-2.580284	11.332287	5.433810	H	-0.706592	6.957858	10.222218
H	-2.926949	9.888023	4.450479	H	0.340709	8.380197	10.376802
C	1.035928	10.689879	8.487916	C	0.931470	5.484017	8.528223
H	0.763426	11.746943	8.455580	H	1.206705	5.258097	7.495970
H	2.071696	10.591011	8.812356	H	-0.054208	5.051102	8.720951
H	0.404783	10.210676	9.243378	H	1.648524	4.986670	9.189444
C	-1.409549	7.995945	3.975032	C	4.278397	9.999686	6.307380
C	-1.413050	8.041242	2.569477	H	3.614008	9.774548	5.471064
C	-2.313736	7.219627	1.882141	C	5.710963	9.822951	5.794612
H	-2.327155	7.241757	0.795968	H	6.452125	10.186615	6.514974
C	-3.195476	6.389097	2.562296	H	5.849348	10.391338	4.871423
H	-3.893056	5.764958	2.010996	H	5.933217	8.773962	5.576048
C	-3.174812	6.352822	3.952682	C	4.040830	11.461635	6.708426
H	-3.861831	5.697562	4.480846	H	4.319414	12.128403	5.885505
C	-2.279015	7.136499	4.683736	H	4.646648	11.725388	7.582535
C	-0.497138	8.965521	1.795022	N	2.543529	8.257845	3.822656
H	0.066489	9.555802	2.521706	C	3.017428	9.313204	3.129765
C	0.505667	8.172105	0.948451	C	4.311559	9.037279	2.606010
H	1.143421	7.544455	1.575367	N	4.661586	7.759346	2.823245
H	1.153347	8.852349	0.387511	H	4.635864	7.545430	4.348414
H	-0.007582	7.526497	0.227507	B	3.572039	7.173845	3.649157
C	-1.291932	9.947063	0.924628	C	2.295238	10.585417	2.941377
H	-1.829194	9.429373	0.122316	C	1.674799	11.253613	3.998116
H	-0.613009	10.671507	0.465035	H	1.762012	10.861882	5.001691
H	-2.028277	10.499371	1.516798	C	0.954937	12.425812	3.777553
C	-2.287720	7.070662	6.202506	H	0.471074	12.916876	4.616814
H	-1.426116	7.636426	6.568380	C	0.857529	12.954241	2.492178
C	-2.133117	5.637467	6.718485	H	0.293079	13.865381	2.315766
H	-2.124079	5.633439	7.813338	C	1.500152	12.310482	1.431603
H	-1.195048	5.199169	6.367734	H	1.438369	12.720184	0.427550
H	-2.957293	4.993155	6.395035	C	2.215497	11.139314	1.653037
C	2.602668	8.550485	7.601159	H	2.714796	10.638512	0.830051
C	2.296005	7.597747	8.593220	C	5.270301	10.000757	2.023542
C	3.323342	7.180494	9.446587	C	5.311304	11.352576	2.401839
H	3.105421	6.449406	10.220139	H	4.606460	11.726588	3.137613
C	4.611944	7.679556	9.321942	C	6.253162	12.219273	1.852598
H	5.394359	7.347399	9.998421	H	6.269379	13.260492	2.163738
C	4.905063	8.588395	8.310688	C	7.177953	11.753049	0.918539
H	5.921738	8.950236	8.201400	H	7.912560	12.429573	0.490423
C	3.920617	9.030443	7.422677	C	7.156502	10.408635	0.544263
C	0.909393	6.999369	8.770664	H	7.876217	10.033383	-0.178484
H	0.244874	7.443994	8.023254	C	6.213808	9.542673	1.090868

H	6.198143	8.494212	0.808524
N	3.271555	5.749548	3.781109
C	4.379104	4.887128	4.023948
C	5.075297	4.248089	2.978135
C	6.145560	3.404790	3.291644
H	6.681483	2.916466	2.478877
C	6.563254	3.191509	4.605855
C	5.880256	3.859138	5.624694
H	6.191525	3.715755	6.658399
C	4.796916	4.698503	5.358137
C	4.715357	4.485261	1.536044
H	3.885453	3.844979	1.212165
H	5.567919	4.269397	0.885478
H	4.418175	5.526055	1.380207
C	7.699442	2.252599	4.917694
H	7.332968	1.233855	5.097460
H	8.243423	2.564359	5.814948
H	8.414212	2.198891	4.090513
C	4.088764	5.405247	6.476706
H	4.406173	6.451899	6.554246
H	4.284813	4.934053	7.443254
H	3.012594	5.430927	6.297556
C	2.082367	5.185574	3.158894
H	2.229747	4.891249	2.108454
H	1.318224	5.968303	3.154403
C	1.566757	3.970940	3.924956
H	1.280688	4.286999	4.947731
H	2.393401	3.258991	4.029567
N	0.475570	3.293403	3.237414
C	0.207991	1.999435	3.840263
H	1.110488	1.380553	3.812577
H	-0.124378	2.075067	4.894950
C	-0.734560	4.097845	3.201552
H	-0.562081	5.052256	2.699470
H	-1.130772	4.320155	4.208765
H	-1.513389	3.571578	2.640477
H	2.994044	11.659211	6.951137
H	-0.575379	1.481833	3.276363
C	-3.548425	7.732750	6.774845
H	-4.451461	7.210796	6.439240
H	-3.531630	7.708166	7.869853
H	-3.629907	8.777612	6.461205

TS-AlHBH-1.log

SCF (wB97x) = -2208.99322233

E(SCF)+ZPE(0 K)= -2207.907953

H(298 K)= -2207.846140

G(298 K)= -2208.004812

Lowest Frequency = -278.9989cm-1

Al	0.514704	7.596569	5.942362
H	-0.049319	6.281354	6.689684
N	-1.109884	8.535805	5.359658
C	-1.562995	9.671724	5.887994
C	-0.921631	10.363706	6.926145
H	-1.413323	11.268815	7.265270
C	0.202319	9.960372	7.670682
N	0.942271	8.904776	7.360493
C	-2.878718	10.249050	5.420337
H	-2.830144	11.340691	5.406185
H	-3.670642	9.965064	6.120497
H	-3.163973	9.888190	4.431531
C	0.493115	10.760218	8.918220
H	0.367958	11.830094	8.732534
H	1.492349	10.574373	9.312319
H	-0.231530	10.477841	9.689677
C	-1.936983	7.776744	4.472331
C	-1.661615	7.799016	3.085281
C	-2.472258	7.039547	2.237141
H	-2.285764	7.047140	1.168258
C	-3.512298	6.262218	2.739851
H	-4.126832	5.673872	2.064073
C	-3.747144	6.224562	4.108094
H	-4.545766	5.598407	4.496865
C	-2.971042	6.973051	4.999812
C	-0.522457	8.643881	2.538460
H	0.286707	8.589784	3.273895
C	0.035413	8.128289	1.210792
H	0.268313	7.060758	1.256555
H	0.956958	8.665284	0.962804
H	-0.662409	8.290402	0.381147
C	-0.917219	10.120664	2.401430
H	-1.778001	10.233961	1.732370
H	-0.084373	10.696671	1.981498
H	-1.170719	10.564206	3.365974
C	-3.268913	6.894038	6.489550
H	-2.605191	7.593714	7.006633

C	-2.971380	5.495734	7.047495
H	-3.189429	5.461423	8.121096
H	-1.917794	5.244026	6.904769
H	-3.586859	4.733580	6.556097
C	2.079211	8.533894	8.149070
C	1.918250	7.618894	9.209729
C	3.056145	7.215452	9.917432
H	2.948040	6.513623	10.740190
C	4.315828	7.699967	9.590350
H	5.188215	7.377485	10.152470
C	4.458314	8.599575	8.537460
H	5.447451	8.970267	8.286887
C	3.356523	9.026747	7.792077
C	0.555469	7.085976	9.620962
H	-0.182537	7.462701	8.905884
C	0.168642	7.591550	11.018272
H	0.848036	7.196202	11.781921
H	-0.846274	7.269652	11.276391
H	0.207357	8.683243	11.076636
C	0.501042	5.554580	9.563487
H	0.763379	5.197726	8.564379
H	-0.510692	5.203064	9.794191
H	1.184716	5.099892	10.289288
C	3.539979	10.013502	6.649067
H	2.698060	9.870353	5.964166
C	4.830950	9.781039	5.855232
H	5.721113	10.046513	6.436492
H	4.833326	10.412336	4.960453
H	4.919743	8.739001	5.536152
C	3.497949	11.465396	7.149081
H	3.656194	12.160160	6.316454
H	4.285941	11.641093	7.890566
N	2.648529	8.732299	3.659471
C	3.213365	9.229341	2.780068
H	3.178876	7.553102	5.492165
B	2.254570	7.291024	4.768484
C	3.965926	9.781670	1.705497
C	3.456809	10.853176	0.956360
H	2.485320	11.270027	1.201549
C	4.211019	11.364348	-0.094020
H	3.825576	12.192785	-0.680017
C	5.457711	10.812302	-0.394534
H	6.040766	11.215017	-1.217545
C	5.958790	9.744642	0.353347
H	6.926642	9.315988	0.112602
C	5.220142	9.221951	1.408255
H	5.582482	8.377930	1.987552
N	2.473171	6.128358	3.920833
C	3.805335	5.743757	3.613369
C	4.294981	5.842086	2.291535
C	5.634846	5.537448	2.029724
H	6.001550	5.629075	1.007889
C	6.512287	5.132850	3.035831
C	6.007514	5.027733	4.333834
H	6.669832	4.699735	5.133892
C	4.676670	5.318034	4.644677
C	3.412268	6.281806	1.153471
H	2.941198	5.425238	0.655542
H	3.988866	6.820615	0.395549
H	2.610088	6.931831	1.505758
C	7.944800	4.782019	2.728886
H	8.063239	3.701894	2.576422
H	8.612781	5.067584	3.547838
H	8.294149	5.278598	1.818159
C	4.212615	5.177159	6.070829
H	4.365921	6.103929	6.636218
H	4.765021	4.379703	6.578274
H	3.144005	4.957924	6.124441
C	1.384907	5.307058	3.416264
H	1.567831	4.964982	2.390089
H	0.485493	5.929692	3.390498
C	1.159236	4.074797	4.294777
H	0.938333	4.414181	5.325597
H	2.101488	3.513673	4.331179
N	0.119586	3.188707	3.783503
C	0.140941	1.913809	4.480422
H	1.120274	1.439188	4.360780
H	-0.063487	2.011499	5.565019
C	-1.200049	3.796975	3.876421
H	-1.246871	4.724497	3.303285
H	-1.488286	4.034161	4.916496
H	-1.949859	3.116625	3.459603
H	2.540600	11.712444	7.611145
H	-0.613809	1.244338	4.053779
C	-4.719672	7.293093	6.797419
H	-5.426495	6.554392	6.403642
H	-4.876893	7.353053	7.879646
H	-4.980559	8.260762	6.358969

TS-CNXyl-2.log

SCF (wB97x) = -2287.62893993

E(SCF)+ZPE(0 K)= -2286.485680

H(298 K)= -2286.421966

G(298 K)= -2286.580816

Lowest Frequency = -71.9643cm-1

Al	0.812248	8.104838	5.515604
H	0.665313	6.534926	5.654905
N	-0.949176	8.852012	5.311834
C	-1.403738	9.850785	6.073827
C	-0.677800	10.397861	7.142044
H	-1.163077	11.210825	7.669646
C	0.483584	9.883420	7.748335
N	1.250770	8.941536	7.198304
C	-2.797398	10.386182	5.864039
H	-3.207314	10.108743	4.892898
H	-2.798080	11.474913	5.962541
H	-3.463610	9.988146	6.635815
C	0.790158	10.386864	9.137187
H	0.434081	11.411074	9.264374
H	1.851742	10.339862	9.378558
H	0.261802	9.758105	9.861852
C	-1.859079	8.100270	4.491598
C	-1.829123	8.255481	3.089528
C	-2.711683	7.491358	2.321217
H	-2.710541	7.600560	1.241837
C	-3.584131	6.584447	2.912729
H	-4.257975	5.996060	2.296246
C	-3.578964	6.421850	4.292158
H	-4.244611	5.694176	4.748114
C	-2.720706	7.167004	5.106303
C	-0.901706	9.260215	2.431328
H	-0.010083	9.343638	3.060968
C	-0.421073	8.843633	1.040441
H	0.041792	7.851772	1.053922
H	0.324806	9.558607	0.684531
H	-1.236010	8.829189	0.308794
C	-1.559478	10.645487	2.374912
H	-2.492775	10.602439	1.802252
H	-0.888710	11.362627	1.892870
H	-1.793568	11.021388	3.374263
C	-2.709900	6.902679	6.602672
H	-2.033193	7.620039	7.077734
C	-2.159152	5.499592	6.887711
H	-2.105459	5.323115	7.967450
H	-1.157112	5.384250	6.466580
H	-2.804361	4.728472	6.452848
C	2.358050	8.406544	7.948829
C	2.153461	7.287169	8.778367
C	3.226455	6.834290	9.555043
H	3.083330	5.978769	10.209348
C	4.465402	7.455852	9.503848
H	5.283672	7.096420	10.121539
C	4.666044	8.522558	8.633021
H	5.649770	8.974918	8.568553
C	3.633216	9.007278	7.826782
C	0.832851	6.537029	8.843955
H	0.136576	7.004400	8.140086
C	0.200270	6.608382	10.239534
H	0.827580	6.110148	10.986684
H	-0.776179	6.111880	10.240727
H	0.055724	7.642584	10.565677
C	1.025416	5.078900	8.404897
H	1.388738	5.029526	7.375213
H	0.080265	4.531442	8.461124
H	1.746128	4.561226	9.046412
C	3.897938	10.124329	6.829562
H	3.294800	9.886627	5.947685
C	5.355094	10.183421	6.361471
H	6.014040	10.598929	7.132744
H	5.429185	10.834244	5.484629
H	5.728004	9.196532	6.077201
C	3.468305	11.509138	7.336317
H	3.778854	12.281145	6.624132
H	3.939122	11.730992	8.300493
H	4.669080	8.004270	4.535818
B	3.653589	7.685721	3.993262
C	2.542369	10.067383	2.387119
C	1.890622	11.244730	2.813680
C	1.674709	12.250693	1.864904
H	1.180944	13.166193	2.182268
C	2.069686	12.103035	0.539576
H	1.881049	12.897245	-0.177084
C	2.722348	10.938132	0.140970
H	3.042523	10.818011	-0.890791

C	2.982526	9.918078	1.054167
N	3.657271	6.372965	3.345315
C	4.718323	5.473150	3.640356
C	5.493365	4.903368	2.607069
C	6.526015	4.018933	2.930351
H	7.123206	3.596023	2.123610
C	6.824293	3.676834	4.249220
C	6.045865	4.245007	5.258380
H	6.250038	3.983722	6.295847
C	4.994263	5.123649	4.984299
C	5.242300	5.240875	1.162619
H	4.484097	4.582462	0.720285
H	6.155099	5.128635	0.570003
H	4.882119	6.265992	1.061365
C	7.923665	2.698391	4.571305
H	7.538848	1.671701	4.618322
H	8.384352	2.916350	5.540024
H	8.711103	2.714875	3.811207
C	4.176762	5.657420	6.132401
H	4.551853	6.620157	6.495173
H	4.191815	4.964038	6.978161
H	3.136049	5.823179	5.845260
C	2.465208	5.808214	2.724403
H	2.670844	5.443405	1.708106
H	1.740016	6.621313	2.616731
C	1.851238	4.655110	3.522647
H	1.589677	5.015127	4.532822
H	2.619215	3.882187	3.642736
N	0.699961	4.045309	2.863609
C	0.324357	2.814936	3.540207
H	1.168571	2.117909	3.543148
H	0.011348	2.979399	4.590463
C	-0.439880	4.948946	2.795783
H	-0.199729	5.835660	2.206499
H	-0.785347	5.290767	3.788390
H	-1.277422	4.450241	2.299019
H	2.386460	11.593960	7.454138
H	-0.507500	2.338143	3.010697
C	-4.096162	7.080514	7.235399
H	-4.798212	6.319304	6.878614
H	-4.032021	6.983189	8.324257
H	-4.527019	8.058993	7.003558
C	3.690382	8.661984	0.637559
H	4.555714	8.475381	1.280318
H	3.038919	7.788319	0.741947
H	4.023334	8.721716	-0.401718
C	1.428797	11.463761	4.227638
H	0.476302	10.964481	4.427228
H	2.142857	11.079646	4.956836
H	1.284827	12.529583	4.423356
N	2.879596	9.025814	3.270145
C	2.314386	8.464241	4.285207

TS-CO2-1.log

SCF (wB97x) = -2073.16712213

E(SCF)+ZPE(0 K)= -2072.170398

H(298 K)= -2072.111978

G(298 K)= -2072.263909

Lowest Frequency = -123.4975cm-1

Al	0.498990	7.580488	6.029387
H	-0.619080	6.620664	6.927673
N	-0.636664	8.695602	4.971455
C	-1.126037	9.856307	5.408737
C	-0.734885	10.439236	6.627226
H	-1.273422	11.331895	6.921497
C	0.311619	10.039332	7.464666
N	1.053196	8.947801	7.229370
C	-2.160408	10.583556	4.593460
H	-2.079585	11.663302	4.731606
H	-3.157072	10.276413	4.929076
H	-2.081475	10.345246	3.531186
C	0.600218	10.857831	8.693075
H	0.152165	11.850566	8.633319
H	1.672349	10.953039	8.876714
H	0.171838	10.336810	9.557650
C	-0.963131	8.151153	3.684469
C	-0.002147	8.254794	2.651421
C	-0.284737	7.643817	1.427815
H	0.433885	7.710812	0.617750
C	-1.469103	6.938428	1.230726
H	-1.665823	6.465113	0.272767
C	-2.393565	6.835704	2.261950
H	-3.311052	6.274898	2.106147
C	-2.162689	7.434889	3.505688
C	1.284354	9.037618	2.856132
H	1.561006	8.937182	3.911412

C	2.465419	8.506272	2.042444	N	2.044155	5.270539	4.541695
H	2.630588	7.441546	2.228708	C	3.228602	4.711923	3.953628
H	3.377527	9.038001	2.325904	C	3.248320	4.418000	2.578069
H	2.325351	8.653889	0.966159	C	4.417819	3.888488	2.026327
C	1.062983	10.533974	2.598156	H	4.439630	3.669511	0.960218
H	0.759245	10.706694	1.559702	C	5.554895	3.643595	2.796929
H	1.986022	11.094415	2.782818	C	5.509411	3.963405	4.155728
H	0.286991	10.942709	3.250840	H	6.391473	3.793439	4.770250
C	-3.179247	7.266585	4.620556	C	4.368182	4.501474	4.755217
H	-2.854306	7.861526	5.478546	C	2.059448	4.677831	1.688288
C	-3.230653	5.805832	5.084810	H	1.456449	3.771612	1.552041
H	-3.916441	5.691808	5.930307	H	2.388002	4.997024	0.694742
H	-2.246292	5.463293	5.411418	H	1.402560	5.452085	2.092415
H	-3.567699	5.143921	4.279260	C	6.788716	3.031827	2.187490
C	2.329519	8.770879	7.861862	H	6.870044	3.268472	1.122343
C	2.442926	7.992797	9.027544	H	6.769090	1.938800	2.278651
C	3.717022	7.792063	9.569596	H	7.698350	3.382948	2.684216
H	3.823971	7.188424	10.466691	C	4.403198	4.881384	6.213675
C	4.843811	8.349422	8.977833	H	4.603019	5.952076	6.338276
H	5.825420	8.187625	9.414752	H	5.191130	4.332581	6.737054
C	4.714645	9.111502	7.819565	H	3.453677	4.684981	6.717969
H	5.601954	9.538612	7.362224	C	0.903697	4.356460	4.663305
C	3.465671	9.329649	7.232964	H	0.829790	3.726771	3.771644
C	1.232379	7.342702	9.669366	H	-0.005049	4.962725	4.714326
H	0.341905	7.721387	9.157279	C	1.048733	3.458168	5.889643
C	1.091799	7.703763	11.152449	H	0.939078	4.087850	6.795462
H	1.917519	7.297930	11.746944	H	2.068119	3.054132	5.892979
H	0.160993	7.290735	11.554103	N	0.129047	2.331900	5.895591
H	1.076951	8.788450	11.299039	C	0.408417	1.460795	7.026901
C	1.284175	5.821895	9.470833	H	1.446391	1.115107	6.980343
H	1.360954	5.569327	8.408378	H	0.253495	1.958201	8.003178
H	0.380026	5.344854	9.858251	C	-1.266414	2.753699	5.897087
H	2.154870	5.392891	9.979375	H	-1.510966	3.260499	4.959617
C	3.339065	10.165524	5.969698	H	-1.510018	3.430490	6.734638
H	2.392434	9.891016	5.492807	H	-1.909763	1.871070	5.964173
C	4.459477	9.902374	4.959652	H	2.398422	11.900807	6.922232
H	5.429320	10.260443	5.321302	H	-0.242627	0.581849	6.985560
H	4.249442	10.437192	4.027781	C	-4.573437	7.754012	4.205385
H	4.549890	8.837015	4.730846	H	-5.001961	7.121881	3.420007
C	3.262540	11.664131	6.297171	H	-5.254483	7.722772	5.061918
H	3.179352	12.251682	5.376355	H	-4.546931	8.779692	3.825054
H	4.164224	11.988847	6.828560	O	-2.165161	7.715736	7.955474
H	3.090835	7.167590	4.896585	O	-1.778780	5.470092	8.254010
B	2.063570	6.567386	5.047390	C	-1.714273	6.605842	7.880075

7) References

[S1] Cui C., Roesky H. W., Schmidt H., Noltemeyer M., Hao H., Cimpoesu F. *Angew. Chem. Int. Ed.*, **2000**, *39*, 4275-4276.

[S2] Phillips. N.A., O'Hanlon J., Hooper T. N., White A. J. P., Crimmin M. R. *Org. Lett.*, **2019**, *21*, 7289-7293.

[S3] Dehmel M., Köhler A., Görlsa H., Kretschmer R. *Dalton Trans.*, **2021**, *50*, 8434–8445.

[S4] Dolomanov O.V.; Bourhis L. J.; Gildea R. J.; Howard J. A. K.; Puschmann H. *J. Appl. Cryst.*, **2009**, *42*, 339-341.

[S5] SHELXTL v5.1, Bruker AXS, Madison, WI, 1998.

[S6] SHELX-2013, Sheldrick G. M. *Acta Cryst.*, **2015**, *C71*, 3-8.

[S7] Spek A. L. (2003, 2009) PLATON, A Multipurpose Crystallographic Tool, Utrecht University, Utrecht, The Netherlands. See also Spek A. L. *Acta. Cryst.*, 2015, **C71**, 9-18.

[S8] Frisch, M. J.; Trucks, G. W.; Schlegel, H. B.; Scuseria, G. E.; Robb, M. A.; Cheeseman, J. R.; Scalmani, G.; Barone, V.; Mennucci, B.; Petersson, G. A.; Nakatsuji, H.; Caricato, M.; Li, X.; Hratchian, H. P.; Izmaylov, A. F.; Bloino, J.; Zheng, G.; Sonnenberg, J. L.; Hada, M.; Ehara, M.; Toyota, K.; Fukuda, R.; Hasegawa, J.; Ishida, M.; Nakajima, T.; Honda, Y.; Kitao, O.; Nakai, H.; Vreven, T.; Montgomery, J. A., Jr.; Peralta, J. E.; Ogliaro, F.; Bearpark, M.; Heyd, J. J.; Brothers, E.; Kudin, K. N.; Staroverov, V. N.; Kobayashi, R.; Normand, J.; Raghavachari, K.; Rendell, A.; Burant, J. C.; Iyengar, S. S.; Tomasi, J.; Cossi, M.; Rega, N.; Millam, J. M.; Klene, M.; Knox, J. E.; Cross, J. B.; Bakken, V.; Adamo, C.; Jaramillo, J.; Gomperts, R.; Stratmann, R. E.; Yazyev, O.; Austin, A. J.; Cammi, R.; Pomelli, C.; Ochterski, J. W.; Martin, R. L.; Morokuma, K.; Zakrzewski, V. G.; Voth, G. A.; Salvador, P.; Dannenberg, J. J.; Dapprich, S.; Daniels, A. D.; Farkas, Ö.; Foresman, J. B.; Ortiz, J. V.; Cioslowski, J.; Fox, D. J. Gaussian 09, Revision D.01; Gaussian, Inc., Wallingford, CT, 2009.

[S9] Glendening E. D.; Landis C. R.; Weinhold. F.; *J. Comput. Chem.*, **2013**, *34*, 1429–1437.

[S10] Keith T. A.; AIMALL (Version 19.10.12). TK Gristmill Software, Overland Park KS, USA., 2019.

[S11] Cortés-Guzmán F.; Bader R. F. W. *Coord. Chem. Rev.*, **2005**, *249*, 633–662.

coordinates.xyz
This file cannot be rendered in this PDF. Please download the source file.
